# Intraspecific and heteroplasmic variations, gene losses and inversions in the chloroplast genome of *Astragalus membranaceus*

**DOI:** 10.1038/srep21669

**Published:** 2016-02-22

**Authors:** Wanjun Lei, Dapeng Ni, Yujun Wang, Junjie Shao, Xincun Wang, Dan Yang, Jinsheng Wang, Haimei Chen, Chang Liu

**Affiliations:** 1 College of Life Science, Shanxi Agricultural University, Shanxi, P.R. China; 2 Institute of Medicinal Plant Development, Chinese Academy of Medical Sciences, Peking Union Medical College, Beijing, P.R. China; 3 Research Center of Medicinal Plants, Shandong Academy of Agricultural Sciences, Shandong, P.R. China

## Abstract

*Astragalus membranaceus* is an important medicinal plant in Asia. Several of its varieties have been used interchangeably as raw materials for commercial production. High resolution genetic markers are in urgent need to distinguish these varieties. Here, we sequenced and analyzed the chloroplast genome of *A. membranaceus* (Fisch.) Bunge var. mongholicus (Bunge) P.K. Hsiao using the next generation DNA sequencing technology. The genome was assembled using Abyss and then subjected to gene prediction using CPGAVAS and repeat analysis using MISA, Tandem Repeats Finder, and REPuter. Finally, the genome was subjected phylogenetic and comparative genomic analyses. The complete genome is 123,582 bp long, containing only one copy of the inverted repeat. Gene prediction revealed 110 genes encoding 76 proteins, 30 tRNAs, and four rRNAs. Five intra-specific hypermutation loci were identified, three of which are heteroplasmic. Furthermore, three gene losses and two large inversions were identified. Comparative genomic analyses demonstrated the dynamic nature of the Papilionoideae chloroplast genomes, which showed occurrence of numerous hypermutation loci, frequent gene losses, and fragment inversions. Results obtained herein elucidate the complex evolutionary history of chloroplast genomes and have laid the foundation for the identification of genetic markers to distinguish *A. membranaceus* varieties.

Astragali Radix (AR), also known as Huangqi, is one of the most popular herbal medicines worldwide. As indicated in the Chinese pharmacopeia, AR is composed of dried roots of two *Astragalus membranaceus* varieties, namely, *A. membranaceus* (Fisch.) Bunge var. membranaceus and *A. membranaceus* (Fisch.) Bunge var. mongholicus (Bunge) P. K. Hsiao^1^. More than 100 compounds, including flavonoids, saponins, polysaccharides, and amino acids have been identified in AR. In addition, various biological activities of these compounds have been reported^2^,^3^,^4^. Traditionally, AR is used to treat weakness, wounds, anemia, fever, multiple allergies, chronic fatigue, loss of appetite, and uterine bleeding and prolapse^5^. Meanwhile, calycosin is the major bioactive isoflavonoid isolated from AR, and its potential pharmaceutical properties in the treatment of tumors, inflammation, stroke, and cardiovascular diseases have recently gained increasing attention^6^.

With the growing demand for AR, the raw materials for AR production are rapidly diminishing in China. Meanwhile, the cultivated *Astragalus* has become an important source of commercial AR in China^7^. *A. membranaceus* (Fisch.) Bunge var. mongholicus (Bunge) P. K. Hsiao is the most widely cultivated variety, although several other varieties have also been used as the raw material for commercial AR production. The inherent differences among these varieties might cause drug efficacy and safety issues. Unfortunately, the lack of molecular markers distinguishing the various varieties of *A. membranaceus* has hindered genetic diversity studies on *A. membranaceus,* and at least partly contributed to the gradual loss of some varieties. Thus, identification of molecular markers in AR is important not only in the screening of high-quality varieties of *Astragalus* but also in the conservation of wild *Astragalus*.

Previous studies suggested that chloroplast genome sequences, which have increasing phylogenetic resolution at lower taxonomic levels, are effective tools in plant phylogenetic and genetic population analyses^8^. The typical chloroplast genome in angiosperms has a conserved quadripartite structure, with two copies of an inverted repeat (IR) separating the large single copy (LSC) and small single copy (SSC) regions^9^. These genomes usually encode 120–130 genes with sizes in the range of 120–170 kb. The gene content and gene order are generally conserved, although a number of variations at the genome and gene levels among the chloroplast genomes in legumes have been reported. These variations include the loss of one copy of the IR^10^, the occurrence of inversions of 50 kb^11^ and 78 kb long^12^, the loss of the *infA*^13^, *rpl22*, and *rps16 *genes^14^, and the loss of introns, such as those in the *rpl2*, *clpP*, and *rps12 *genes^14^,^15^.

Here, we sequenced and annotated the complete chloroplast genome of *A. membranaceus* (Fisch.) Bunge var. mongholicus (Bunge) P. K. Hsiao as a first step to identify genetic markers that can distinguish the varieties of *A. membranaceus*. The genomes are highly conserved in terms of the genic and genomic structures compared with those from other Papilionoideae species. Comparative genomic analyses showed that this genome belongs to the inverted-repeat-lacking clade (IRLC). In addition, two inversions and numerous gene losses have been identified. However, these inversions and/or gene loss events are probably associated with Papillonidae as a whole, as we did not find any such events that can distinguish *A. membranaceus* from other Papillonidae species. Most importantly, we have identified five intraspecific hypermutation regions and 262 simple sequence repeat (SSR) loci. Three of the hypermutation loci are heteroplasmic. These hypermutation regions could be used as effective markers to study the genetic diversities among *A. membranaceus* varieties.

## Results

### General features of the *A. membranaceus* chloroplast genome

**Figure 1:**
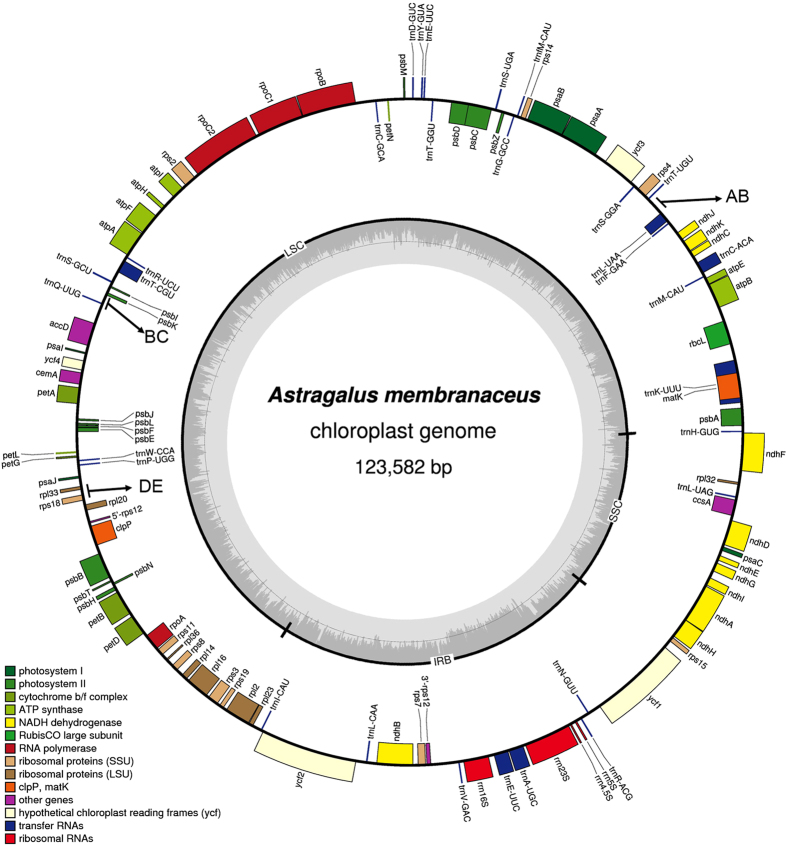
Schematic representation of the *A. membranaceus* chloroplast genome. The predicted genes are shown and colors represent functional classifications, which are shown at the left bottom. The genes drawn outside the circle are transcribed clockwise, whereas those drawn inside the circle are transcribed counter-clockwise. The inner circle shows the GC content. The large single copy (LSC), small single copy (SSC) and inverted repeat (IR) regions are shown in the inner circle. The three hypermutation regions (AB, BC and DE) are indicted with arrows.

Unless specified, *A. membranaceus* refers to *A. membranaceus* (Fisch) Bunge var. Mongholicus (Bunge) P. K. Hsiao in this paper for simplicity. The chloroplast genome was completely sequenced by a combination of de novo assembly and gap filling, as described below. All raw sequence reads were mapped up to the final assembly, and a total of 6,023,406 out of 15,000,362 (40.2%) pair-end reads were successfully mapped. The remaining unmapped reads possibly represent contaminant mitochondrial or nuclear DNAs (data not shown). In addition, the average coverage is approximately 9800. The complete chloroplast genome sequence is 123,582 bp long with only one copy of the IR region. Moreover, a total of 110 genes were identified, including 76 protein-coding genes, 30 transfer RNA (tRNA) genes, and four ribosome RNA (rRNA) genes (Table 1). The general structure and locations of the 110 genes in the chloroplast genome are depicted in Fig. 1. The LSC (bases 1–80986), SSC (bases 109810–123582) and IR region (80987–109809) regions are shown. The IR region is defined by the stop codons of genes *rps19* and *ycf1*. Meanwhile, the genes *rps16* and *rpl22*, which are found in most angiosperm plastid genomes including representatives of the early-branching lineages^16^,^17^,^18^, are absent in *A. membranaceus*. In addition, a total of 17 genes in *A. membranaceus* chloroplast genome have only one intron (Table S1); *ycf3* is the only with two intron*s*. Similarly, introns in the 3′-end of *rps12*, a trans-splicing gene are also absent. Moreover, the *accD* gene of *A. membranaceus* encodes a protein with 451 amino acids, which is shorter than the other *accD* proteins (Fig. S1). Furthermore, *infA* was not found in the chloroplast genome of *A. membranaceus*; this gene codes for translation initiation factor 1 and is suspected to be an example of chloroplast-to-nucleus gene transfer^13^. The implication of this finding needs further investigation.

Overall, 60.5% of the *A. membranaceus* chloroplast genome sequence is composed of genes that code proteins. The overall GC content of the *A. membranaceus* chloroplast genome comprises 34.1%, whereas the protein-coding regions comprise 36.0%. Within the protein-coding regions, the GC contents for the first, second and third positions of the codons comprise 44.9%, 37.3% and 27.4%, respectively. The codon usage and codon-anticodon recognition pattern of the *A. membranaceus* chloroplast genome are summarized in Table S2. The 30 tRNA genes contain codons corresponding to all 20 amino acids that are necessary for biosynthesis. Among these genes, six contain an intron, as follows: *trnK-UUU*, *trnC-ACA*, *trnL-UAA*, *trnT-CGU*, *trnE-UUC*, and *trnA-UGC*. The lengths of these introns range from 543 bp to 2494 bp.

### Repeat and SSR analysis

SSRs are valuable molecular markers of high-degree variations within the same species and have been used in population genetics and polymorphism investigations^19^. We analyzed the occurrence, type, and distribution of SRRs in the *A. membranaceus* chloroplast genome and the distribution of SSRs in 13 other IRLC chloroplast genomes belonging to Papilionoideae. In total, 262 SSRs were identified in *A. membranaceus* chloroplast genome (Table S3, Table 2). Among these SSRs, the majority consisted of mono- and di- nucleotide repeats, which were found 148 and 89 times, respectively. Tri- (12), tetra- (11), penta- nucleotide repeat sequences (1) were found with much lower frequency. This observed pattern is similar to those observed in 13 IRLC chloroplast genomes of other species belonging to Papilionoideae (Table S4). Most mononucleotide repeat sequences consisted of A/T repeats (99.3%). Similarly, 86.5% of the dinucleotide repeat sequences consisted of AT/AT repeats (Table S3). Our findings are in agreement with the previous findings that the chloroplast SSRs are generally composed of short polyA or polyT repeats and rarely contained tandem G or C repeats^20^. In this study, we also analyzed the locations of 24 tri-, tetra- and penta- nucleotides in the chloroplast genome, and the results are shown in Table 2. Among these nucleotides, 21 are localized in the intergenic regions, and 3 are in the coding regions.

Seven forward repeats were identified using REPuter with a size cutoff of 30 bp (Table 3). The longest forward repeat unit was 114 bp long and was located in the intergenic region of *trn*N-GUU and *ycf1*. Six tandem repeats longer than 30 bp were identified, and the similarities among these repeat units were >90%. All of these tandem repeats were located in the intergenic regions (Table 3).

### Presence of hypermutation regions in *A. membranaceus* chloroplast genome

The initial whole genome de novo assembly revealed seven scaffolds labeled as A, B, C, D, E, F, and G. To close these gaps, we designed seven sets of primers spanning the adjacent scaffolds. PCR products were easily obtained using the primer pairs spanning the gaps between scaffolds A and B, B and C, as well as D and E (Fig. S2); however, DNA sequencing for these three PCR products could not generate high-quality DNA sequences. Manual examination of the trace files suggested the presence of multiple and similar, but non-identical, sequences in these PCR products (Fig. S2). In particular, the quality of the sequences in these PCR products significantly dropped after the poly A/T stretches, which are located in the intergenic regions between the genes *trnF-GAA* and *trnT-UGU* (region AB, bases 14421–15192), *psbK* and *trnQ-UUG* (region BC, bases 53416–54021), and *rpl33* and *rps18* (region DE, bases 65175–65575). The start and end positions of these regions were determined by the 3′ ends of the corresponding PCR primers used for their amplification. These regions probably contained low complexity sequences of variable length.

**Figure 2:**
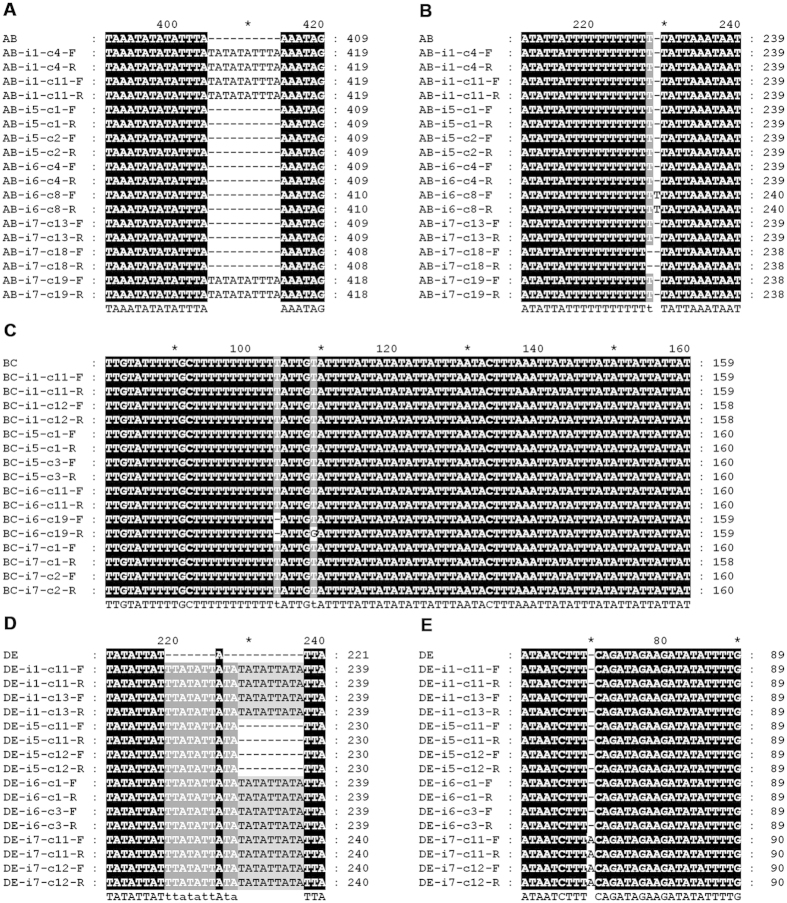
Alignment of sequences from the PCR products for the identification of highly polymorphic regions in the *A. membranaceus* chloroplast genomes. Panels (**A,B**) show the sequences obtained from the region AB. Panel (**C**) is for the region BC. Panels (**D,E**) show the sequences obtained from the region DE. The ID of each sequence is shown on the left side of each panel. The ID is the concatenation of region name, plant individual id, clone ID and primer direction (F: forward; R: reverse).

To determine the exact structure of these polymorphic regions, DNA from four plant individuals, named i1, i5, i6 and i7 were extracted. PCR amplification was performed and the PCR products were cloned. Ten positive clones for each PCR product were selected and sequenced. The sequences of all fragments with high quality were aligned with MegAlign (DNASTAR, WI) using the CLUSTALW2 algorithm (Fig. S3). Five variable loci: vl1, vl2, vl3, vl4 and vl5 are shown in Fig. 2A–E respectively. The name of each sequence follows the format [name of genome region]-[id of plant individual]-[clone id]-[primer direction]. For the locus vl1 (Fig. 2A), an extra copy of “TATATATTTA” repeat was found in i1, which were absent in i5 and i6. In i7, sequences from one out of three clones (AB-i7-c19) contain the extra copy “TATATATTTA”. In contrast, the sequences from the other two clones AB-i7-c13 and AB-i7-c18 did not have the extra copy. For the locus vl2 (Fig. 2B), we observed a single nucleotide insertion and deletion in the sequences from clones AB-i6-c8 and AB-i7-c18, respectively compared to the consensus sequences. It is noted that this region is rich in “A”. For the locus vl3 (Fig. 2C), a single nucleotide deletion was observed in the sequences from one clone of i6 (BC-i6-c19). For the locus vl4, (Fig. 2D), an extra copy of “TATATTATA” was observed in all sequences of i1, i6 and i7 comparing to those of i5, which is the repeat unit between genes *rpl33* and *rps18*. For the locus vl5 (Fig. 2E), there was an insertion of a single nucleotide “A” in the sequences from all clones of i7. All five loci are intraspecific variations. Among them, vl1, vl2 and vl3 are also heteroplasmic. These intra-specific loci represent markers that can potentially be used to distinguish closely related varieties of *Astragalus membranaceu.*

### Phylogenetic analysis of *A. membranaceus* based on conserved protein sequences

**Figure 3:**
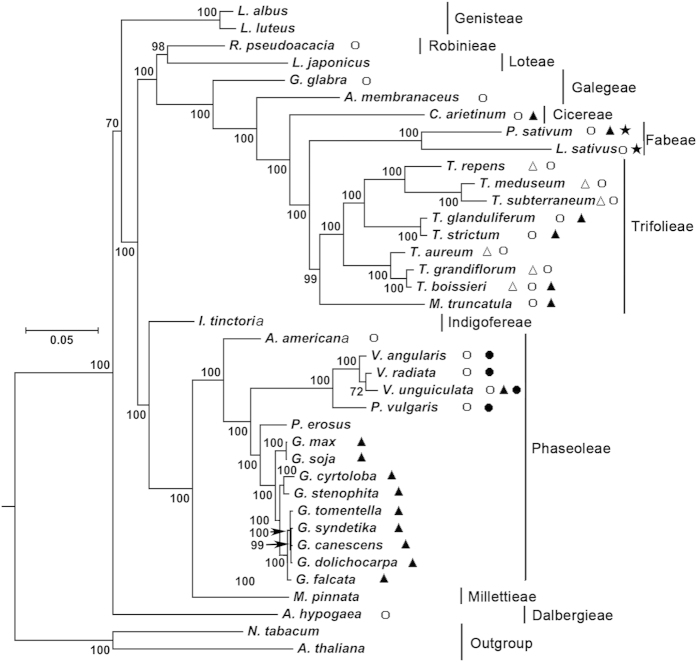
Molecular phylogenetic analysis of the Papilionoideae subfamily. The tree was constructed with the sequences of 67 proteins present in all 38 species (*Lupinus albus, Lupinus luteus, Robinia pseudoacacia, Lotus japonicus, Glycyrrhiza glabra, Astragalus membranaceus, Cicer arietinum, Pisum sativum, Lathyrus sativus, Trifolium repens, Trifolium meduseum, Trifolium subterraneum, Trifolium glanduliferum, Trifolium strictum, Trifolium aureum,Trifolium grandiflorum, Trifolium boissieri, Medicago truncatula, Indigofera tinctoria, Apios americana, Vigna angularis, Vigna radiata, Vigna unguiculata, Phaseolus vulgaris, Pachyrhizus erosus, Glycine max, Glycine soja, Glycine cyrtoloba, Glycine stenophita, Glycine tomentella, Glycine syndetika, Glycine canescens, Glycine dolichocarpa, Glycine falcata, Millettia pinnata, Arachis hypogaea, Arabidopsis thaliana, Nicotiana tabacum*), using the Maximum Likelihood method implemented in RAxML. Two taxa, *Nicotiana tabacum* and *Arabidopsis thaliana* were used as outgroups. The tribes, to which each species belongs, are shown to the right side of the tree. Bootstrap supports were calculated from 1000 replicates. Genes lost in a particular branch were indicated with the following symbols: ○(rps16), ▲(ycf4), △(accD), •(rpl23) and ●(rpl33).

To determine the phylogenetic position of *A. membranaceu* of in Papilionoideae, 37 complete chloroplast genome sequences were obtained from the RefSeq database (Table S5). *Nicotiana tabacum* and *Arabidopsis thaliana* were included in the analysis as the outgroup taxa. The other 35 species belong to Cicereae (1), Dalbergieae (1), Fabeae (1), Galegeae (1), Indigofereae (1), Loteae (1), Millettieae (1), Robinieae (1), Genisteae (2), Trifolieae (9), and Phaseoleae (15) respectively. The number shown in the parenthesis represents the number of species in the corresponding clade. To conduct phylogenetic analysis, we extracted 67 protein sequences, which were present among all the 38 chloroplast genomes. There were a total of 18515 positions in the final dataset. Results showed that *A. membranaceus* is the closest sister species of *Glycyrrhiza glabra* and *Cicer arietinum* with bootstrap values of 100% (Fig. 3). The symbols next to each species represent genes that were found lost. More details on gene losses are shown in Table 4. Overall, the patterns of gene loss are consistent with the tree topology with a few exceptions. For example, *ycf*4 was found lost in *V. unguiculata*, but not in the closely related species *V. angularis* and *V. radiate*. In addition, *ycf*4 was found lost in *T. boissieri*, but not in the closely related *T. grandiflorum* and *T. aureum*. These findings suggest that the loss of *ycf*4 occurred after the geneses of *Vigna* and *Trifolium* species.

### Frequent inversions in the chloroplast genomes of Papilionoideae

**Figure 4:**
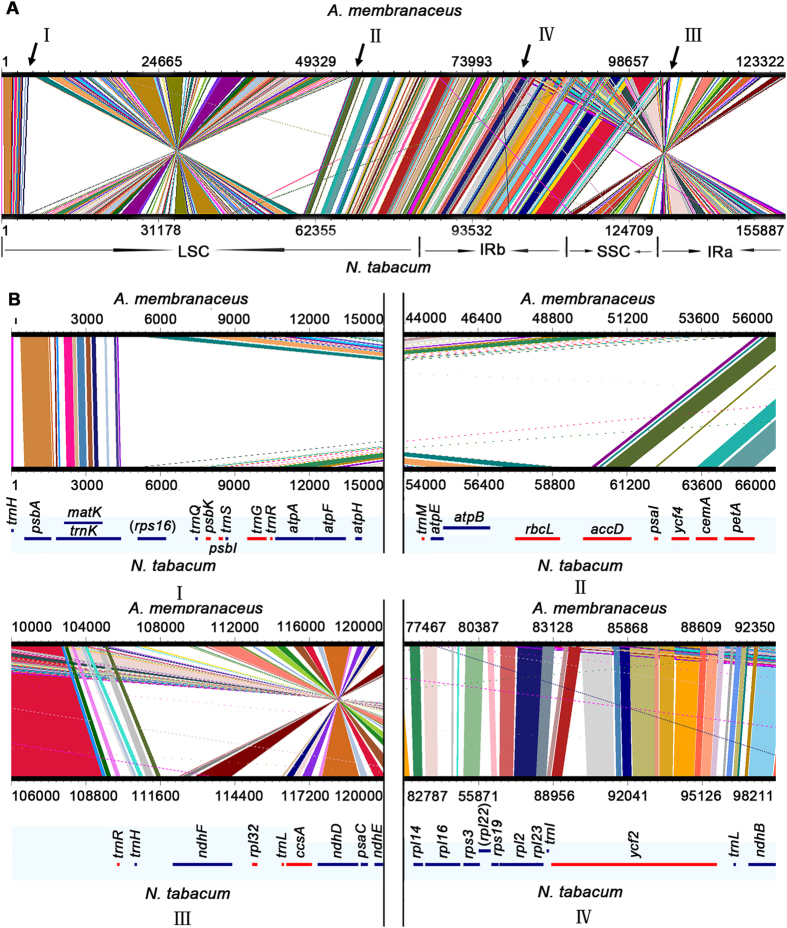
Synteny analyses of chloroplast genomes from *A. membranaceus* and *N. tabacum*. (**A**) Global synteny view; LSC region, the IRa and IRb and SSC regions are shown at the bottom of the alignment. I, II, III, and IV represent the border regions of the two inversions (enlarged and shown below); (**B**) Detailed alignments of the border regions of two inversions between *A. membranaceus* and *N. tabacum*. The coding regions of genes are represented by lines below the synteny maps, with their names shown on top of the lines. Blue and red colors indicate that the genes are transcribed clockwise and counterclockwise, respectively. The genes lost in *A. membranaceus* are enclosed in parentheses.

**Figure 5:**
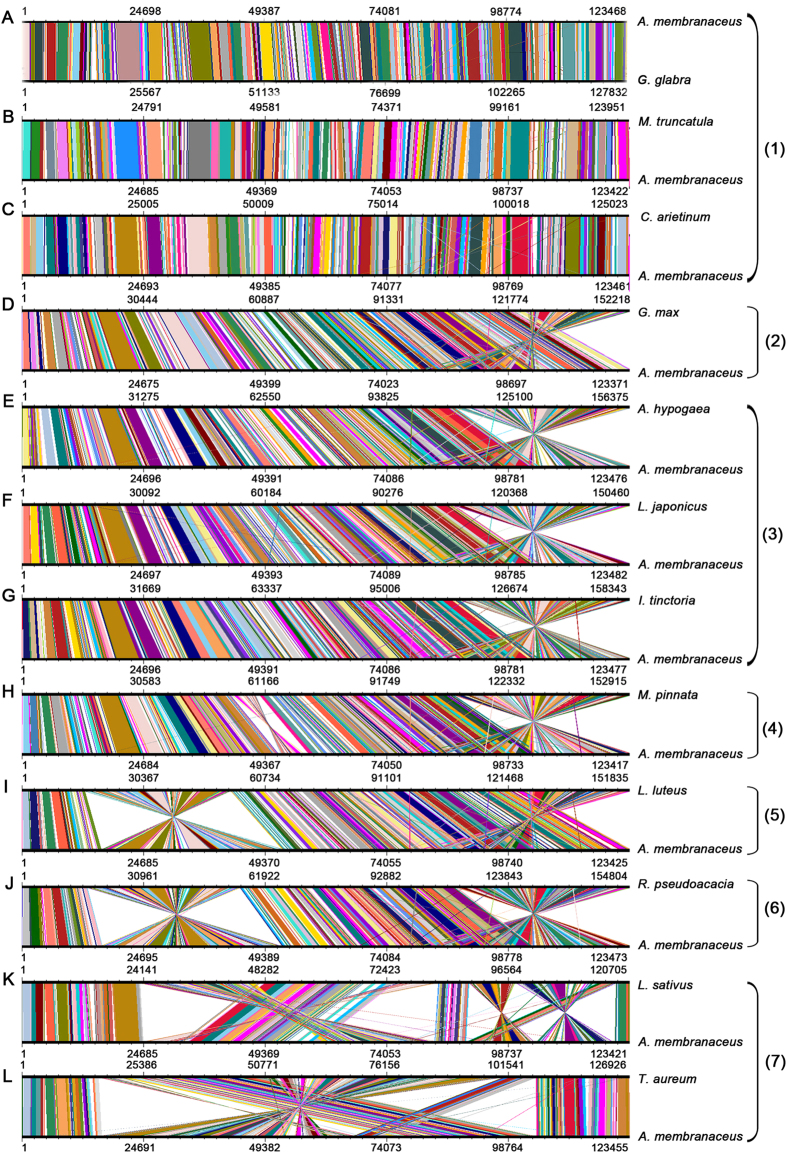
Comparative genomic analyses of thirteen chloroplast genomes. The chloroplast genome of *A. membranaceus* was aligned with those of twelve species. Each horizontal black line represents a genome. The species names are shown to the right of the corresponding line. The conserved regions are bridged by lines. The numbers on the right of each panel indicates the group number to which the chloroplast genomes have been assigned.

To identify the possible occurrence of genome rearrangement, the chloroplast genome sequences of *A. membranaceus*, *N. tabacum* and 12 other species belonging to Papilionoideae were selected for synteny analyses. These 12 species include *C. arietinum*, *Arachis hypogaea*, *Lathyrus sativus*, *G. glabra*, *Lupinus luteus*, *Indigofera. tinctoria*, *Lotus japonicus*, *Millettia pinnata*, *Glycine max*, *Robinia pseudoacacia*, *Medicago truncatula*, and *Trifolium aureum*, which are members of the tribes Cicereae, Dalbergieae, Fabeae, Galegeae, Genisteae, Indigofereae, Loteae, Millettieae, Phaseoleae, Robinieae, and Trifolieae, respectively (Figs 4 and 5).

Two inversions are readily discernible between chloroplast genomes of *N. tabacum* and *A. membranaceus* (Fig. 4A). The genes at the enlarged inversion boundaries (I, II, III and IV) are shown in Fig. 4B. A large inversion of 50 kb, which is apparently shared by the majority of papilionoid legumes^21^, is located between the *rps16* (Fig. 4B–I) and *rbcL* genes (Fig. 4B–II). Similarly, the other notable inversion of 20 kb is located between the *ndhF* (Fig. 4B–III) and *ycf1* genes (Fig. 4B–IV). This inversion has also been found in other species such as *G. glabra*, *M. truncatula* and *C. arietinum* (Fig. 5)

The 12 species were classified into seven groups based on the degree of genome conservation relative to the *A. membranaceus* chloroplast genome. The first group includes *C. arietinum*, *M. truncatula,* and *G. glabra*. The gene order of the chloroplast genomes of this group is highly conserved compared with that of *A. membranaceus* (Fig. 5A–C). Particularly, these chloroplast genomes had only one copy of the IR. The second group includes *G. max* (Fig. 5D), whose chloroplast genome structure is similar to that of *A. membranaceus,* except for the presence of two copies of the IR. The third group includes *A. hypogaea*, *L. japonicus*, and *I. tinctoria* whose genomes contain the 20 kb inversions in the SSC region and two copies of the IR (Fig. 5E–G). The fourth group includes *M. pinnata*, whose genome contains not only the 20 kb inversion in the SSC region but also one small inversion in the LSC region (Fig. 5H). The fifth group includes *L. luteus*, whose genome contains the 50 kb inversion in the LSC region (Fig. 5I). The sixth group includes *R. pseudoacacia*, whose genome includes two large inversions. One is of approximately 50 kb long in the LSC region and the other one is 20 kb long in the SSC regions (Fig. 5J). All chloroplast genomes of the second to sixth groups have two copies of the IR. The seventh group includes two IRLC species, namely, *L. sativus* and *T. aureum* whose chloroplast genomes contain numerous inversions (Fig. 5K,L). These results suggested that inversions frequently occurred in the evolution of Papilionadeae.

### Comparative analyses of the gene losses among the chloroplast genomes in Papilionoideae

The loss of genes in the chloroplast genomes of Papilionoideae was then analyzed in detail (Table 4). The species names were order based on that shown in Fig. 4. And the gene names were ordered based on the number of species in which the gene was found lost. The *rpl22* gene was absent in all 36 chloroplast genomes of Papilionoideae. In addition, *rps16* gene was not found in the chloroplast genomes of 21 completely sequenced Papilionoideae species, including all IRLC species. Moreover, the loss of *ycf4* gene was observed in 16 chloroplast genomes. The loss of *accD* was observed in six *Trifolium* genomes. Loss of the *rpl33* and *rpl23* genes occurred in four chloroplast genomes (*P. vulgaris*, *V. radiata*, *V. unguiculata*, and *V. angularis*) and in two chloroplast genomes (*P. sativum* and *L. sativus*), respectively. The losses of *ndhD*, *psaI*, *rps18*, and *rps19* were only found in *V. angularis*, *L. sativus*, *T. subterraneum*, and *R. pseudoacacia*, respectively. The most frequently lost genes *rps*16, *ycf*4 and *rpl*33 were found to locate at the boundaries of the 50 kb inversion, suggesting that their losses might be related to the genesis of this 50 kb inversion. The patterns of gene loss were found to be largely consistent with the topology of the phylogenetic tree (Fig. 3).

## Discussion

In the present study, we have: (1) sequenced the chloroplast genome of *A. membranaceus*; (2) annotated the chloroplast genome; (3) identified SSR and tandem repeats of the genome; (4) carried out a phylogenetic analysis of the 38 chloroplast genomes based on 67 conserved proteins; (4) compared the structures of 13 chloroplast genomes in Papilionoideae; (5) identified genes that have been lost among the 36 chloroplast genomes in Papilionoideae subfamily; and (6) identified five hypermutation loci that can potentially serve as markers to distinguish *A. membranaceus* varieties. Our results have laid the foundation for future studies on the evolution of chloroplast genomes of legumes, as well as the molecular identification of *A. membranaceus* varieties.

PCR products with primers spanning the targeted gaps are directly obtained during gap filling; however, obtaining sequencing results of good quality in these three regions was difficult. After checking the trace files, we hypothesize that this regions largely contains low-complexity sequences and might be highly polymorphic. DNA samples from four plant individuals were extracted. The corresponding regions were amplified. The PCR products were then cloned and sequenced. The results revealed five hypermutative regions, which contain variable repeat numbers or single nucleotide indels (Fig. 2). Furthermore, variations were also observed among sequences derived from the same plant individual (vl1, vl2 and vl3), a manifestation of heteroplasmy. This finding also explains why the de novo genome assembly program failed to assemble the genome at these regions in the first place.

Moreover, the current study demonstrated high degree of diversity in the structure of legume chloroplast genomes. Genome organization and gene content of chloroplast genomes is believed to be highly conserved in most angiosperms^22^. With the increasing number of chloroplast genome sequences, the diverse organization of chloroplast genome is becoming more evident, as demonstrated by the extensive genome rearrangement and gene losses in the chloroplast genomes of the legume family. For example, all members of the Carmichaelieae, Cicereae, Hedysareae, Trifolieae, Fabeae (Vicieae), Galegeae tribes, and three genera of Millettieae contain only one copy of the IR and are thereby assigned as belonging to the IRLC^15^. Furthermore, the losses of *rpl22*, rps*16*, and *ycf4* have been reported in various chloroplast genomes^15^. These genomic rearrangements combined with variations at the gene structural levels provided valuable information to resolve relationships among several deep nodes of legumes^21^,^23^,^24^,^25^.

Whether or not there are any links among hypermutation, inversion and gene loss is an interesting question. Compared with that of *N. tabacum*, two large inversions have been identified in the *A. membranaceus* chloroplast genome (Fig. 4A). The 50 kb inversion was identified in the LSC region between *rps16* and *rbcL* in *N. tabacum* (Fig. 4B), while *rps16* was absent in the *A. membranaceus* chloroplast genome. From a systematical analysis of gene losses in 36 other species (Table 4), it is found that three of the most frequently lost genes, namely, *accD*, *rps16*, and *ycf4*, are located at the boundaries of the 50 kb inversion. While one of them, the *ycf4* has not been lost in *A. membranaceus*, its loss has been found in *Lathyrus odoratus* and three other groups of legumes. Particularly, each of the four consecutive genes *ycf4-psaI-accD-rps16* has been lost in at least one member of the legume’s IRLC^25^. In contrast to the 50 kb inversion, gene losses were not observed at the boundaries of the 20 kb inversion. Hypermutation has been implicated in gene loss before. For example, a 1.5 kb long region of chloroplast DNA in plants related to sweetpea (Lathyrus) was found to be coincides with *ycf4*, whose local point mutation rate is at least 20 times higher than elsewhere in the same molecule^25^. In *A. membranaceus*, the three hypermuation regions found are not adjacent to any of the inversions. Taking together, while the inversions and gene losses are likely to be associated based on their adjacency in *A. membranaceus*, the relationship between inversion and hypermuation is not evident.

In the future, we plan to apply the same approach to sequence and analyze more chloroplast genomes from *A. membranaceus* varieties. Comparative analyses will likely provide insight into the chloroplast genome evolution of *A. membranaceus* varieties. Furthermore, detailed characterization of the highly polymorphic regions is another interesting direction. Samples from individual plants belonging to different varieties of *A. membranaceus* can be collected. Primers specific to these regions can be used to amplify these regions for sequencing. Alignment of these sequences can be used to determine the degree of variations at the individual, population, variety, and species levels. This information will facilitate the establishment of an effective DNA barcoding-based identification method and provide valuable markers to study the population genetics of *A. membranaceus*.

## Methods

### Plant material and chloroplast DNA purification

Fresh leaves of *A. membranaceus* from multiple individuals were collected from the fields of Institute of Medicinal Plant Development, Beijing, China and stored at 4 °C for chloroplast genomic DNA isolation. Chloroplasts were isolated from approximately 100 g fresh leaves using the high salt saline plus Percoll gradient method described before^26^. Subsequently, chloroplast DNA was extracted from the purified chloroplasts, and the chloroplast DNA purity was evaluated with 1.0% agarose gel, whereas DNA concentration was measured using a Nanodrop spectrophotometer 2000 (Thermo Fisher Scientific, America).

### Chloroplast genome sequencing, assembly and gap filling

Approximately 50 ng of chloroplast DNA was sheared to yield approximately 500 bp long fragments for paired-end library construction according to the manufacturer’s instructions (Illumina Inc., San Diego, CA). The library was sequenced on Illumina HiSeq 2000 (Illumina Inc.). In total, 15,000,362 paired-end reads (2 × 100 bp) were obtained.

To identify a reference genome to assist the assembly, we first downloaded 27 chloroplast genomes belonging to the Papilionoideae from GenBank in December 2014. These chloroplast genome sequences were used to search against Illumina paired-end reads using BLASTN with an E-value cutoff of 1e-5. The genome sequence of *G. glabra* (Accession number: NC_024038) had the highest overall sequence similarity to the reads and was used as a reference for the downstream genome assembly.

AbySS (v1.5.2)^27^ was used for the *De novo* genome assembly. Different k-mer sizes were tested. The k-mer size of 64 gave the best results in terms of the smallest numbers of scaffolds and the longest average length of scaffolds. And this parameter was used to generate the final assembly.

The resulting contigs were compared against the chloroplast genome sequence of *G. glabra* using BLASTN with an E-value cutoff of 1e-5. Seven large contigs were identified and were temporarily arranged based on their mapping positions on the reference genome. Moreover, primers were designed based on the sequences at the ends of the adjacent contigs. PCR amplification and subsequent DNA sequencing were used to fill the gaps. PCR amplifications were performed using the sequence specific primers (Table S6) under the following conditions: predenaturation at 94 °C for 2 min, 35 cycles of amplification at 94 °C for 30 s, 55 °C for 30 s and 72 °C for 30 s, followed by a final extension at 72 °C for 2 min. The PCR reaction mixture contained 25 μl of Taq MasterMix (2×), 2 μl of forward primer (10 μM), 2 μl of reverse primer (10 μM), purified chloroplast DNA (<1 μg). RNase-free water was added to the final reaction volume of 50 μl.

The correctness of the assembly was validated further by mapping all raw sequence reads to the assembly using Bowtie 2 (v2. 0.1) program^28^ with the default settings. Manual examination of the coverage of the entire assembly was performed using Tablet (v1.14.10.20)^29^. The primer sequences are listed in Table S6.

### Genome annotation and codon usage analyses

The CpGAVAS web service^30^ was used to annotate the *A. membranaceus* chloroplast genome. Cutoffs for the E-values of BLASTN and BLASTX were 1e-10. The number of top hits to be included in the reference gene sets for annotation after the pre-filtering step was 10. Meanwhile, tRNA genes were identified using tRNAscan-SE^31^ and ARAGORN^32^. Manual corrections on the positions of the start and stop codons, and for the intron/exon boundaries were performed based on the entries in the Chloroplast Genome Database^33^ using the Apollo program^34^. Moreover, the circular chloroplast genome map of *A. membranaceus* was drawn using OrganellarGenomeDRAW^35^. Furthermore, codon usage and GC content were analyzed using the Cusp and Compseq programs provided by EMBOSS^36^. Final genome assembly and genome annotation results were deposited in the GenBank (accession number: KU666554).

### Repeat sequence analysis

SSRs were detected using MISA Perl Script available at (http://pgrc.ipk-gatersleben.de/misa/), with the following thresholds: 8 repeat units for mononucleotide SSRs, 4 repeat units for di- and trinucleotide repeat SSRs, and 3 repeat units for tetra-, penta-, and hexanucleotide repeat SSRs. Tandem repeats were analyzed using Tandem Repeats Finder^37^ with parameter settings of 2 for matches and 7 for mismatches and indels. The minimum alignment score and maximum period size were set at 50 and 500, respectively. All the identified repeats were manually verified and nested or redundant results were removed. REPuter^38^ was employed to identify the IRs in *A. membranaceus* by forward vs. reverse complement (palindromic) alignment. The minimal repeat size was set at 30 bp, and the cutoff for similarities among the repeat units was set at 90%.

### Phylogenetic analysis

A total of 37 complete chloroplast DNA sequences belonging to the Papilionoideae subfamily were obtained from RefSeq database (Table S5). For the phylogenetic analysis, 67 protein sequences shared among all these 37 species and *A. membranaceus* were aligned using the CLUSTALW2 (v2.0.12) program. The 67 proteins are ATPA, ATPB, ATPE, ATPF, ATPH, ATPI, CCSA, CEMA, CLPP, MATK, NDHA, NDHB, NDHC, NDHE, NDHF, NDHG, NDHH, NDHI, NDHJ, NDHK, PETA, PETB, PETD, PETG, PETL, PETN, PSAA, PSAB, PSAC, PSAJ, PSBA, PSBB, PSBC, PSBD, PSBE, PSBF, PSBH, PSBI, PSBJ, PSBK, PSBL, PSBM, PSBN, PSBT, PSBZ, RBCL, RPL14, RPL16, RPL2, RPL20, RPL36, RPOA, RPOB, RPOC1, RPOC2, RPS11, RPS12, RPS14, RPS15, RPS2, RPS3, RPS4, RPS7, RPS8, YCF1, YCF2 and YCF3 (Supplementary file 1). The alignment was manually examined and adjusted. Then, the evolutionary history was inferred using the Maximum Likelihood method implemented in RaxML (v8.2.4)^39^. The detailed parameters were “raxmlHPC-PTHREADS-SSE3 -f a -N 1000 -m PROTGAMMACPREV -x 551314260 -p 551314260 -o A_thaliana,N_tabacum -T 20”. The tree with the highest log likelihood (−233993.753326) was shown. The significance level for the phylogenetic tree was assessed by bootstrap testing with 1000 replications. Only branches supported by bootstrap values >50% are shown.

### Comparative genome analysis

Conserved sequences were identified between the chloroplast genomes of *A. membranaceus* and those of *N. tabacum* (NC_001879), *C. arietinum* (NC_011163), *A.hypogaea* (NC_026676), *L. sativus* (NC_014063), *G. glabra* (NC_024038), *L. albus* (NC_023090), *I. tinctoria* (NC_026680), *L. japonicus* (NC_002694), *M. pinnata* (NC_016708), *G. max* (NC_007942), *R. pseudoacacia* (NC_026684), *M. truncatula* (NC_003119), and *T. aureum* (NC_024035) using BLASTN with an E-value cutoff of 1e-10. The homologous regions and gene annotations were visualized using a web-based genome synteny viewer GSV^40^.

### Examination of hypermutation regions in *A. membranaceus* chloroplast genome by PCR amplification, PCR product cloning and DNA sequencing

To determine the structure of the likely hypermutation regions in *A. membranaceus* chloroplast genome, the total DNA of four *A. membranaceus* individuals were extracted independently using the plant genomic DNA kit (Tiangen Biotech, Beijing) and subjected to PCR amplification using the PrimeSTAR max DNA polymerase (*Takara* Bio, Japan), a high fidelity polymerase. The primers specific for the gaps between scaffolds A and B, B and C, as well as D and E were used (Table S6). The PCR reactions were performed under the following conditions: pre-denaturation at 95 °C for 1 min, 40 cycles of amplification at 98 °C for 10 s, 53 °C for 15 s and 72 °C for 10 s, followed by a final extension at 72 °C for 2 min. The PCR products were purified with TIANquick Midi Purification Kit (Tiangen Biotech) and cloned using Lethal Based Fast Cloning Kit (Tiangen Biotech). For each region from each individual plant, 10 positive clones were selected and sequenced by Sanger method. A total of 120 clones were sequenced in both the forward and reverse direction by Sinogenomax Co., Ltd (Beijing).

## Additional Information

**How to cite this article**: Lei, W. *et al.* Intraspecific and heteroplasmic variations, gene losses and inversions in the chloroplast genome of *Astragalus membranaceus.* *Sci. Rep.* **6**, 21669; doi: 10.1038/srep21669 (2016).

## Supplementary Material

Supplementary Information

Supplementary Dataset 1

## Figures and Tables

**Table 1:** Genes predicted in the chloroplast genome of *A. membranaceus*.

Category of genes	Group of genes	Name of genes
Self-replication	rRNA genes	*rrn16S*, *rrn23S*, *rrn 4.5S*, *rrn 5S*
	tRNA genes	30 *trn* genes (6 contain an intron)
	Small subunit of ribosome	*rps2*, *rps3*, *rps4*, *rps7*, rps8, rps11, rps12*, rps14, rps15, rps18, rps19
	Large subunit of ribosome	rpl14, rpl16*, rpl2*, rpl20, rpl23, rpl32, rpl33, rpl36
	DNA dependent RNA polymerase	rpoA, rpoB, rpoC1*,rpoC2
Genes for photosynthesis	Subunits of NADH-dehydrogenase	ndhA*, ndhB*, ndhC, ndhD, *ndhE, ndhF, ndhG, ndhH, ndhI, ndhJ, ndhK*
	Subunits of photosystem I	psaA, psaB, psaC, psaI, psaJ, ycf3**
	Subunits of photosystem II	*psbA, psbB, psbC, psbD, psbE, psbF, psbH, psbI, psbJ, psbK, psbL, psbM, psbN, psbT, psbZ*
	Subunits of cytochrome b/f complex	petA, petB*, petD*, petG, petL, petN
	Subunits of ATP synthase	atpA, atpB, atpE, atpF*, atpH, atpI
	Subunit of rubisco	*rbcL*
Other genes	Maturase	*matK*
	Protease	clpP*
	Envelope membrane protein	*cemA*
	Subunit of Acetyl-CoA-carboxylase	*accD*
	C-type cytochrome synthesis gene	*ccsA*
Genes of unkown function	Conserved open reading frames	*ycf1, ycf2, ycf4*

^*^The number of asterisks after the gene names indicates the number of introns contained in the genes.

**Table 2:** Distribution of tri-, tetra-, and penta- nucleotide SSR loci in the chloroplast genome of *A. membranaceus*.

SSR type	SSR sequence	Start	End	Location
tri	(TAT)4	4483	4494	IGS^a^(*matK*-*rbcL*)
tri	(ATA)4	32341	32352	IGS(*petN*-*trnC-GCA*)
tri	(TAT)4	45484	45495	IGS(*rps2*-*atp*I)
tri	(ATT)4	51038	51049	IGS(*trnR-GCU*-*trnS-GCU*)
tri	(TAT)4	53563	53574	IGS(*psbK*-*trnQ-UUG*)
tri	(TAT)4	54247	54258	IGS(*trnQ-UUG*-*accD*)
tri	(TAT)4	60883	60894	IGS(*petA*-*psbJ*)
tri	(ATA)4	64011	64022	IGS(*trnP-UGG*-*psaJ*)
tri	(TAA)4	83455	83463	IGS(*rpl23*-*trnI-CAU*)
tri	(AAT)4	94710	94721	IGS(*rps12*-3′-*trnV-GAC*)
tri	(ATA)4	114770	114781	IGS(*ndhI*-*ndhG*)
tri	(TAA)4	120393	120404	IGS(*trnL-UAG*-*rpl32*)
tetra	(ATAG)3	1686	1697	IGS(*psbA*-*matK*)
tetra	(TTTA)3	10123	10134	IGS(*trnM-CAU*-*ndhC*)
tetra	(CTTA)3	47735	47746	IGS(*atpH*-*atpF*)
tetra	(ATAG)3	55121	55132	IGS(*trnQ-UUG*-*accD*)
tetra	(TCTT)3	62405	62416	IGS(*psbE*-*petL*)
tetra	(TAAT)3	83444	83455	IGS(*rpl23*-*trnI-CAU*)
tetra	(ATAG)3	90687	90698	IGS(*trnL-CAA*-*ndhB*)
tetra	(AGGT)3	101290	101301	CDS^b^(*rrn23S*)
tetra	(CAAA)3	108493	108504	CDS(*ycf1*)
tetra	(TATT)3	118107	118118	CDS(*ndhD*)
tetra	(AAAT)3	119565	119576	IGS(*ccsA*-*trnL-UAG*)
penta	(TATAT)3	65384	65398	IGS(*rpl33*-*rps18*)

^a^intergenic spacer region, ^*b*^coding sequences.

**Table 3:** Repeat sequences identified in the chloroplast genome of *A. membranaceus*.

Repeat Number	Repeatsize (bp)	Type	Location	Repeat Unit sequence
1	52	F	CDS^a^ (*psaA*), CDS (*psaB*)	CTATGGCTGACCGATATTGCACATCATCATTTAGCTATTGCAATTCTTTTTC
2	48	F	IGS^*b*^ (*accD*-*psaI*), IGS (*psaI*-*ycf4*)	CAAAAAAGAACAGGTACAAATATAAAATTGAGGTACCCATTTTATGAT
3	41	F	introns(*rpl16*), IGS(*rps12*-*trnV-GAC*)	TTACAGAACCGTACATGAGATTTTCACCTCATACGGCTCCT
4	38	F	IGS(*rpl23*-*trnI-CAU*), CDS(*ycf2*)	GTCTGGATTCAAATCCTACTGAAAGGTCCAGTAGAGAT
5	30	F	IGS(*rpl23*-*trnI-CAU*), IGS(*rpl23*-*trnI-CAU*)	AAATAATAATCTAATTGAAGTTTAGTAATT
6	83	F	IGS(*trnN-GUU*-*ycf1*), IGS(*trnN*-*GUU-ycf1*)	TATTATAACATAACAAATTATAACATAACAAAATCATATATATAATTATCATATTATAACATAACAAATTATAACATAACAAA
7	114	F	IGS(*trnN-GUU*-*ycf1*), IGS(*trnN-GUU*-*ycf1*)	TATATAATTATCATATTATAACATAACAAATTATAACATAACAAATAACATAACAAAATCATACATATAACATATAATTATCATATTATAACATAACAAATTATAACATAACAA
8	42	T	IGS(*ycf3*-*psaA*)	AAAGAGGAGGACTCAATGATT (Х2)
9	72	T	IGS(*rpl33*-*rps18*)	ATTATTTATATTATATAT(Х4)
10	30	T	IGS(*rpl23*-*trnI-CAU*)	AATTAATTAT (Х3)
11	280	T	IGS(*trnN*-*GUU-ycf1*)	ATTATAACATAACAAAATAACATAACAAAACATACATATAATATAATTATCATATTATAACATAACAA (Х4)
12	32	T	IGS(*trnL-UAG*-*rpl32*)	ATATATTATAATATAT (Х2)
13	36	T	IGS(*trnL-UAG*-*rpl32*)	TAAATATTCTTATATTAC (Х2)

^a^coding sequences; ^*b*^intergenic spacers.

**Table 4:** Gene losses in the chloroplast genomes of the Papilionoideae subfamily.

Name of species	rpl22	rps16^a^	ycf4^a^	accD^a^	rpl33	rpl23	ndhD	psaI	rpl32	rps18	rps19
*L. albus*	**−**	**+**	**+**	**+**	**+**	**+**	**+**	**+**	**+**	**+**	**+**
*L. luteus*	**−**	**+**	**+**	**+**	**+**	**+**	**+**	**+**	**+**	**+**	**+**
*R. pseudoacacia*	**−**	**−**	**+**	**+**	**+**	**+**	**+**	**+**	**+**	**+**	**−**
*L. japonicus*	**−**	**+**	**+**	**+**	**+**	**+**	**+**	**+**	**+**	**+**	**+**
*G. glabra*	**−**	**−**	**+**	**+**	**+**	**+**	**+**	**+**	**+**	**+**	**+**
*A. membranaceus*	**−**	**−**	**+**	**+**	**+**	**+**	**+**	**+**	**+**	**+**	**+**
*C. arietinum*	**−**	**−**	**−**	**+**	**+**	**+**	**+**	**+**	**+**	**+**	**+**
*P. sativum*	**−**	**−**	**−**	**+**	**+**	**−**	**+**	**+**	**+**	**+**	**+**
*L. sativus*	**−**	**−**	**+**	**+**	**+**	**−**	**+**	**−**	**+**	**+**	**+**
*T. repens*	**−**	**−**	**+**	**−**	**+**	**+**	**+**	**+**	**+**	**+**	**+**
*T. meduseum*	**−**	**−**	**+**	**−**	**+**	**+**	**+**	**+**	**+**	**+**	**+**
*T. subterraneum*	**−**	**−**	**+**	**−**	**+**	**+**	**+**	**+**	**−**	**−**	**+**
*T. glanduliferum*	**−**	**−**	**−**	**+**	**+**	**+**	**+**	**+**	**+**	**+**	**+**
*T. strictum*	**−**	**−**	**−**	**+**	**+**	**+**	**+**	**+**	**+**	**+**	**+**
*T. aureum*	**−**	**−**	**+**	**−**	**+**	**+**	**+**	**+**	**+**	**+**	**+**
*T. grandiflorum*	**−**	**−**	**+**	**−**	**+**	**+**	**+**	**+**	**+**	**+**	**+**
*T. boissieri*	**−**	**−**	**−**	**−**	**+**	**+**	**+**	**+**	**+**	**+**	**+**
*M. truncatula*	**−**	**−**	**−**	**+**	**+**	**+**	**+**	**+**	**+**	**+**	**+**
*I. tinctoria*	**−**	**+**	**+**	**+**	**+**	**+**	**+**	**+**	**+**	**+**	**+**
*A. americana*	**−**	**−**	**+**	**+**	**+**	**+**	**+**	**+**	**+**	**+**	**+**
*V. angularis*	**−**	**−**	**+**	**+**	**−**	**+**	**−**	**+**	**+**	**+**	**+**
*V. radiata*	**−**	**−**	**+**	**+**	**−**	**+**	**+**	**+**	**+**	**+**	**+**
*V. unguiculata*	**−**	**−**	**−**	**+**	**−**	**+**	**+**	**+**	**+**	**+**	**+**
*P. vulgaris*	**−**	**−**	**+**	**+**	**−**	**+**	**+**	**+**	**+**	**+**	**+**
*P. erosus*	**−**	**+**	**+**	**+**	**+**	**+**	**+**	**+**	**+**	**+**	**+**
*G. max*	**−**	**+**	**−**	**+**	**+**	**+**	**+**	**+**	**+**	**+**	**+**
*G. soja*	**−**	**+**	**−**	**+**	**+**	**+**	**+**	**+**	**+**	**+**	**+**
*G. cyrtoloba*	**−**	**+**	**−**	**+**	**+**	**+**	**+**	**+**	**+**	**+**	**+**
*G. stenophita*	**−**	**+**	**−**	**+**	**+**	**+**	**+**	**+**	**+**	**+**	**+**
*G. tomentella*	**−**	**+**	**−**	**+**	**+**	**+**	**+**	**+**	**+**	**+**	**+**
*G. syndetika*	**−**	**+**	**−**	**+**	**+**	**+**	**+**	**+**	**+**	**+**	**+**
*G. canescens*	**−**	**+**	**−**	**+**	**+**	**+**	**+**	**+**	**+**	**+**	**+**
*G. dolichocarpa*	**−**	**+**	**−**	**+**	**+**	**+**	**+**	**+**	**+**	**+**	**+**
*G. falcata*	**−**	**+**	**−**	**+**	**+**	**+**	**+**	**+**	**+**	**+**	**+**
*M. pinnata*	**−**	**+**	**+**	**+**	**+**	**+**	**+**	**+**	**+**	**+**	**+**
*A. hypogaea*	**−**	**−**	**+**	**+**	**+**	**+**	**+**	**+**	**+**	**+**	**+**
Total number of missing gene	**36**	**21**	**16**	**6**	**4**	**2**	**1**	**1**	**1**	**1**	**1**

^a^genes located at the boundaries of the 50 kb inversions.

## References

[b1] Fu J. *et al.* Review of the botanical characteristics, phytochemistry and pharmacology of *Astragalus membranaceus* (Huangqi). Phytother. Res. 28, 1275–1283 (2014). 25087616 10.1002/ptr.5188

[b2] Chu C. *et al.* Radix Astragali (Astragalus): latest advancements and trends in chemistry, analysis, pharmacology and pharmacokinetics. Curr. Org. Chem. 14, 1792–1807 (2010).

[b3] Tang L., Liu Y., Wang Y. & Long C. Phytochemical analysis of an antiviral fraction of Radix astragali using HPLC-DAD-ESI-MS/MS. J. Nat. Med. 64, 182–186 (2010). 20037801 10.1007/s11418-009-0381-1

[b4] Xu F. *et al.* Absorption and metabolism of Astragali radix decoction: in silico, *in vitro* and a case study *in vivo*. Drug Metab. Dispos. 34, 913–924 (2006). 16507649 10.1124/dmd.105.008300

[b5] Kim C. *et al.* Induction of growth hormone by the roots of *Astragalus membranaceus* in pituitary cell culture. Arch. Pharm. Res. 26, 34–39 (2003). 12568355 10.1007/BF03179928

[b6] Gao J., Liu Z. J., Chen T. & Zhao D. Pharmaceutical properties of calycosin, the major bioactive isoflavonoid in the dry root extract of Radix astragali. Pharm. Biol. 52, 1217–1222 (2014). 24635389 10.3109/13880209.2013.879188

[b7] Ma X. Q., Duan J. A., Zhu D. Y., Dong T. T. & Tsim K. W. Species identification of Radix Astragali (Huangqi) by DNA sequence of its 5S-rRNA spacer domain. Phytochemistry. 54, 363–368 (2000). 10897476 10.1016/s0031-9422(00)00111-4

[b8] Parks M., Cronn R. & Liston A. Increasing phylogenetic resolution at low taxonomic levels using massively parallel sequencing of chloroplast genomes. BMC Biol. 7, 84 (2009). 19954512 10.1186/1741-7007-7-84 PMC2793254

[b9] Jansen R. K. *et al.* Methods for obtaining and analyzing whole chloroplast genome sequences. Methods Enzymol. 395, 348–384 (2005). 15865976 10.1016/S0076-6879(05)95020-9

[b10] Palmer J. D. & Thompson W. F. Chloroplast DNA rearrangements are more frequent when a large inverted repeat sequence is lost. Cell. 29, 537–550 (1982). 6288261 10.1016/0092-8674(82)90170-2

[b11] Palmer J. D. & Thompson W. F. Rearrangements in the chloroplast genomes of mung bean and pea. Proc. Natl. Acad. Sci. USA 78, 5533–5537 (1981). 16593087 10.1073/pnas.78.9.5533 PMC348780

[b12] Bruneau A., Doyle J. J. & Palmer J. D. A Chloroplast DNA Inversion as a Subtribal Character in the Phaseoleae (Leguminosae). Syst. Bot. 15, 378–386 (1990).

[b13] Millen R. S. *et al.* Many parallel losses of *infA* from chloroplast DNA during angiosperm evolution with multiple independent transfers to the nucleus. Plant Cell. 13, 645–658 (2001). 11251102 10.1105/tpc.13.3.645 PMC135507

[b14] Doyle J. J., Doyle J. L. & Palmer J. D. Multiple independent losses of two genes and one intron from legume chloroplast genomes. Syst. Bot. 20, 272–294 (1995).

[b15] Jansen R. K., Wojciechowski M. F., Sanniyasi E., Lee S. B. & Daniell H. Complete plastid genome sequence of the chickpea (*Cicer arietinum*) and the phylogenetic distribution of *rps12* and *clpP* intron losses among legumes (Leguminosae). Mol. Phylogenet. Evol. 48, 1204–1217 (2008). 18638561 10.1016/j.ympev.2008.06.013 PMC2586962

[b16] Goremykin V. V., Hirsch-Ernst K. I., Wolfl S. & Hellwig F. H. Analysis of the *Amborella trichopoda* chloroplast genome sequence suggests that *Amborella* is not a basal angiosperm. Mol. Biol. Evol. 20, 1499–1505 (2003). 12832641 10.1093/molbev/msg159

[b17] Hansen D. R. *et al.* Phylogenetic and evolutionary implications of complete chloroplast genome sequences of four early-diverging angiosperms: *Buxus* (Buxaceae), *Chloranthus* (Chloranthaceae), *Dioscorea* (Dioscoreaceae) and *Illicium* (Schisandraceae). Mol. Phylogenet. Evol. 45, 547–563 (2007). 17644003 10.1016/j.ympev.2007.06.004

[b18] Raubeson L. A. *et al.* Comparative chloroplast genomics: analyses including new sequences from the angiosperms *Nuphar advena* and *Ranunculus macranthus*. BMC Genomics. 8, 174 (2007). 17573971 10.1186/1471-2164-8-174 PMC1925096

[b19] Xue J., Wang S. & Zhou S. L. Polymorphic chloroplast microsatellite loci in *Nelumbo* (Nelumbonaceae). Am. J. Bot. 99, e240–244 (2012). 22615305 10.3732/ajb.1100547

[b20] Kuang D. Y. *et al.* Complete chloroplast genome sequence of *Magnolia kwangsiensis* (Magnoliaceae): implication for DNA barcoding and population genetics. Genome. 54, 663–673 (2011). 21793699 10.1139/g11-026

[b21] Doyle J. J., Doyle J. L., Ballenger J. A. & Palmer J. D. The distribution and phylogenetic significance of a 50-kb chloroplast DNA inversion in the flowering plant family Leguminosae. Mol. Phylogenet. Evol. 5, 429–438 (1996). 8728401 10.1006/mpev.1996.0038

[b22] Jansen R. K. *et al.* Analysis of 81 genes from 64 plastid genomes resolves relationships in angiosperms and identifies genome-scale evolutionary patterns. Proc. Natl. Acad. Sci. USA 104, 19369–19374 (2007). 18048330 10.1073/pnas.0709121104 PMC2148296

[b23] Hu J. M., Lavin M., Wojciechowski M. F. & Sanderson M. J. Phylogenetic systematics of the tribe Millettieae (Leguminosae) based on chloroplast trnK/matK sequences and its implications for evolutionary patterns in Papilionoideae. Am. J. Bot. 87, 418–430 (2000). 10719003

[b24] Wojciechowski M. F., Lavin M. & Sanderson M. J. A phylogeny of legumes (Leguminosae) based on analysis of the plastid matK gene resolves many well-supported subclades within the family. Am. J. Bot. 91, 1846–1862 (2004). 21652332 10.3732/ajb.91.11.1846

[b25] Magee A. M. *et al.* Localized hypermutation and associated gene losses in legume chloroplast genomes. Genome Res. 20, 1700–1710 (2010). 20978141 10.1101/gr.111955.110 PMC2989996

[b26] Vieira Ldo N. *et al.* An improved protocol for intact chloroplasts and cpDNA isolation in conifers. PLoS One. 9, e84792 (2014). 24392157 10.1371/journal.pone.0084792 PMC3879346

[b27] Simpson J. T. *et al.* ABySS: a parallel assembler for short read sequence data. Genome Res. 19, 1117–1123 (2009). 19251739 10.1101/gr.089532.108 PMC2694472

[b28] Langmead B., Trapnell C., Pop M. & Salzberg S. L. Ultrafast and memory-efficient alignment of short DNA sequences to the human genome. Genome Biol. 10, R25 (2009). 19261174 10.1186/gb-2009-10-3-r25 PMC2690996

[b29] Milne I. *et al.* Using Tablet for visual exploration of second-generation sequencing data. Brief. Bioinform. 14, 193–202 (2013). 22445902 10.1093/bib/bbs012

[b30] Liu C. *et al.* CpGAVAS, an integrated web server for the annotation, visualization, analysis and GenBank submission of completely sequenced chloroplast genome sequences. BMC Genomics. 13, 715 (2012). 23256920 10.1186/1471-2164-13-715 PMC3543216

[b31] Schattner P., Brooks A. N. & Lowe T. M. The tRNAscan-SE, snoscan and snoGPS web servers for the detection of tRNAs and snoRNAs. Nucleic Acids Res. 33, W686–689 (2005). 15980563 10.1093/nar/gki366 PMC1160127

[b32] Laslett D. & Canback B. ARAGORN, a program to detect tRNA genes and tmRNA genes in nucleotide sequences. Nucleic Acids Res. 32, 11–16 (2004). 14704338 10.1093/nar/gkh152 PMC373265

[b33] Cui L. *et al.* ChloroplastDB: the Chloroplast Genome Database. Nucleic Acids Res. 34, D692–696 (2006). 16381961 10.1093/nar/gkj055 PMC1347418

[b34] Misra S. & Harris N. Using Apollo to browse and edit genome annotations. Curr. Protoc. Bioinformatics. Chapter **9**, Unit 9 5 (2006). 10.1002/0471250953.bi0905s12 18428771

[b35] Lohse M., Drechsel O. & Bock R. OrganellarGenomeDRAW (OGDRAW): a tool for the easy generation of high-quality custom graphical maps of plastid and mitochondrial genomes. Curr. Genet. 52, 267–274 (2007). 17957369 10.1007/s00294-007-0161-y

[b36] Rice P., Longden I. & Bleasby A. EMBOSS: the European Molecular Biology Open Software Suite. Trends Genet. 16, 276–277 (2000). 10827456 10.1016/s0168-9525(00)02024-2

[b37] Benson G. Tandem repeats finder: a program to analyze DNA sequences. Nucleic Acids Res. 27, 573–580 (1999). 9862982 10.1093/nar/27.2.573 PMC148217

[b38] Kurtz S. *et al.* REPuter: the manifold applications of repeat analysis on a genomic scale. Nucleic Acids Res. 29, 4633–4642 (2001). 11713313 10.1093/nar/29.22.4633 PMC92531

[b39] Stamatakis A. RAxML version 8: a tool for phylogenetic analysis and post-analysis of large phylogenies. Bioinformatics. 30, 1312–1313 (2014). 24451623 10.1093/bioinformatics/btu033 PMC3998144

[b40] Revanna K. V., Chiu C. C., Bierschank E. & Dong Q. GSV: a web-based genome synteny viewer for customized data. BMC Bioinformatics. 12, 316 (2011). 21810250 10.1186/1471-2105-12-316 PMC3199762

