# Supplementary material for: Intraspecific and heteroplasmic variations, gene losses and inversions in the chloroplast genome of Astragalus membranaceus
Source: Sci Rep. 2016 Feb 22;6:21669. doi: 10.1038/srep21669 (PMC4761949; doi:10.1038/srep21669)
Supplement: Supplementary Information [file srep21669-s1.pdf]

Supplementary Information

Intraspecific and heteroplasmic variations, gene losses and inversions in the chloroplast genome of *Astragalus membranaceus*

Wanjun Lei[1], Dapeng Ni[3], Yujun Wang[1], Junjie Shao[2], Xincun Wang[2], Dan Yang[2], Jinsheng Wang[1$], Haimei Chen[2$], Chang Liu[2$]

[$]Corresponding authors: Chang Liu cliu6688@yahoo.com, 0086-10-57833111; Haimei Chen, hmchen@implad.ac.cn, 0086-10-57833114; Jinsheng Wang, sxndwjs@163.com, 0086-035- 46289318

[1]College of Life Science, Shanxi Agricultural University, Shanxi, P.R. China
[2]Institute of Medicinal Plant Development, Chinese Academy of Medical Sciences, Peking Union Medical College, Beijing, P.R. China.
[3]Research Center of Medicinal Plants, Shandong Academy of Agricultural Sciences, Shandong, P.R. China

WJL: wjleileo@163.com
DPN: ndp007@163.com
YJW: wangyujun854190@126.com
JJS: shaojie415@126.com
XCW: w822c716@163.com
DY: dyang@implad.ac.cn
JSW: sxndwjs@163.com
HMC: hmchen@implad.ac.cn
CL: cliu@implad.ac.cn

Table S1 Length of introns and exons of the split genes.

	Gene Location			Length (bp)			
Gene Name	Strand	Start	End	Exon I	Intron I	Exon II	Intron II
ycf3	+	16648	18586	117	706	228	735
rpoC1	+	37350	40117	432	713	1623	
atpF	+	47921	49176	168	677	411	
clpP	−	67413	68617	363	615	227	
petB	+	71631	73101	6	823	642	
petD	+	73307	74510	8	712	484	
rpl16	−	78346	79749	9	984	411	
rpl2	−	81189	82728	393	712	435	
ndhB	−	90709	92882	723	689	762	
ndhA	−	111824	114151	552	1236	540	
rps12-5' end	−	67138	67251	114	-	-	
rps12-3' end	−	93682	93939	-	-	258	
trnK-UUU	−	1739	4304	38	2494	36	
trnC-ACA	−	9788	10473	41	589	58	
trnL-UAA	+	13491	14118	36	543	51	
trnT-CGU	−	51154	51931	36	700	44	
trnE-UUC	+	97703	98552	33	778	41	
trnA-UGC	+	98622	99495	38	783	55	

Table S2 Codon usage and codon–anticodon recognition patterns in *A. membranaceus* chloroplast genome.

Codon	Amino acid	Number	tRNA	Codon	Amino acid	Number	tRNA	Codon	Amino acid	Number	tRNA
GCA	A	351	*trnA-UGC*	AAG	K	263		AGC	S	83	*trnS-GCU*
GCC	A	173		CTA	L	310	*trnL-UAG*	AGT	S	361	
GCG	A	120		CTC	L	137		TCA	S	320	*trnS-UGA*
GCT	A	577		CTG	L	122		TCC	S	234	*trnS-GGA*
TGC	C	58	*trnC-GCA*	CTT	L	488		TCG	S	148	
TGT	C	186	*trnC-ACA*	TTA	L	818	*trnL-UAA*	TCT	S	476	
GAC	D	160	*trnD-GUC*	TTG	L	482	*trnL-CAA*	ACA	T	357	*trnT-UGU*
GAT	D	698		ATG	M	502	*trnM-CAU*	ACC	T	191	*trnT-GGU*
GAA	E	882	*trnE-UUC*	AAC	N	251	*trnN-GUU*	ACG	T	102	*trnT-CGU*
GAG	E	256		AAT	N	854		ACT	T	467	
TTC	F	378	*trnF-GAA*	CCA	P	279		GTA	V	475	
TTT	F	923		CCC	P	169		GTC	V	140	*trnV-GAC*
GGA	G	617		CCG	P	99		GTG	V	156	
GGC	G	128	*trnG-GCC*	CCT	P	346	*trnP-UGG*	GTT	V	454	
GGG	G	198		CAA	Q	615	*trnQ-UUG*	TGG	W	372	*trnW-CCA*
GGT	G	540		CAG	Q	163		TAC	Y	151	*trnY-GUA*
CAC	H	105	*trnH-GUG*	AGA	R	369	*trnR-UCU*	TAT	Y	683	
CAT	H	385		AGG	R	128		TAA	*	43	
ATA	I	644		CGA	R	289		TAG	*	15	
ATC	I	366	*trnI-CAU* *trnfM-CAU*	CGC	R	92		TGA	*	18	
ATT	I	973		CGG	R	83					
AAA	K	931	trnK-UUU	CGT	R	276	*trnR-ACG*				

Table S3 Numbers of mono- and dinucleotide SSRs identified in the chloroplast genome of *A. membranaceus*.

Repeat	Number of Copies											
Types	4	5	6	7	8	9	10	11	12	13	14	15	Total
A/T	-	-	-	-	53	33	30	11	8	6	6	1	148
C/G	-	-	-	-	1								1
AC/GT	1												1
AG/CT	10	1											11
AT/AT	49	16	11	1									77

Table S4 Distributions of SSRs in the 14 IRLC chloroplast genomes

Species	Number of SSRs						
	1	2	3	4	5	6	Total
M. truncatula	190	63	9	14	0	2	278
C. arietinum	190	62	5	8	0	1	266
T. subterraneum	241	63	13	11	0	0	328
P. sativum	145	61	6	2	0	1	215
L.sativus	167	44	2	4	0	2	219
T.grandiflorum	174	64	4	5	2	0	249
T. aureum	174	62	5	8	2	0	251
T. repens	204	81	9	9	0	0	303
G. glabra	158	96	7	11	0	0	272
T. meduseum	243	71	8	11	4	1	338
T. boissieri	179	59	1	4	4	0	247
T. glanduliferum	162	66	8	6	3	0	245
T. strictum	156	66	10	11	4	0	247
A.membranaceus	149	89	12	11	1	0	262

Table S5 List of chloroplast genome sequences used in this study.

Tribe	Species Name	Accession	Length (bp)	Protein Number	RNA Number	Created date	Updated date
Cicereae	*Cicer arietinum* / common:chickpea	NC_011163	125319	75	33	08/26/2008	03/26/2010
Dalbergieae	*Arachis hypogaea* / common:peanut	NC_026676	156395	81	44	03/20/2015	03/20/2015
Fabeae	*Lathyrus sativus*	NC_014063	121020	74	34	04/23/2010	12/07/2010
	Pisum sativum / common:pea	NC_014057	122169	74	34	04/23/2010	12/07/2010
Galegeae	*Glycyrrhiza glabra*	NC_024038	127943	76	34	05/01/2014	09/29/2014
Genisteae	*Lupinus albus* / common:white lupine	NC_026681	154140	83	45	03/20/2015	03/20/2015
	Lupinus luteus / common:yellow lupine	NC_023090	151894	83	45	12/20/2013	09/17/2014
Indigofereae	*Indigofera tinctoria*	NC_026680	158367	82	45	03/20/2015	03/20/2015
Loteae	*Lotus japonicus* / strain:Accession MG-20	NC_002694	150519	82	45	03/01/2001	04/15/2009
Millettieae	*Millettia pinnata*	NC_016708	152968	83	45	01/27/2012	01/22/2013
Phaseoleae	*Apios americana*	NC_025909	148772	82	43	12/22/2014	12/22/2014
	Glycine canescens voucher CSIRO:G1232	NC_021647	152518	82	45	07/23/2013	09/06/2013
	Glycine cyrtoloba voucher CSIRO:G1267	NC_021645	152381	81	45	07/23/2013	09/06/2013
	Glycine dolichocarpa voucher CSIRO:G1134	NC_021648	152804	82	45	07/23/2013	09/06/2013
	Glycine falcata voucher CSIRO:G1718	NC_021649	153023	82	45	07/23/2013	09/06/2013
	Glycine max / common:soybean	NC_007942	152218	83	45	04/07/2006	04/15/2009
	Glycine soja	NC_022868	152217	83	46	11/19/2013	11/19/2013
	Glycine stenophita voucher CSIRO:G1974	NC_021646	152618	82	45	07/23/2013	09/06/2013
	Glycine syndetika voucher CSIRO:G1300	NC_021650	152783	82	45	07/23/2013	09/06/2013
	Glycine tomentella voucher CSIRO:G1403 / type:D3	NC_021636	152728	82	45	07/03/2013	07/03/2013

	Pachyrhizus erosus	NC_026682	151947	83	44	03/20/2015	03/20/2015
	Phaseolus vulgaris	NC_009259	150285	83	44	04/03/2007	04/15/2009
	Vigna angularis DNA / common:adzuki bean	NC_021091	151683	81	44	05/17/2013	05/17/2013
	Vigna radiata	NC_013843	151271	82	44	02/18/2010	07/28/2010
	Vigna unguiculata / common:cowpea	NC_018051	152415	84	46	07/02/2012	07/02/2012
Robinieae	*Robinia pseudoacacia*	NC_026684	154835	80	45	03/20/2015	03/20/2015
Trifolieae	*Medicago truncatula* / strain:Variety Jema Long A-17; common:barrel medic	NC_003119	124033	76	33	08/31/2001	04/15/2009
	Trifolium aureum	NC_024035	126970	76	34	04/30/2014	09/29/2014
	Trifolium boissieri	NC_025743	125740	74	36	01/13/2015	01/13/2015
	Trifolium glanduliferum	NC_025744	126149	75	35	12/03/2014	12/03/2014
	Trifolium grandiflorum	NC_024034	125628	75	35	05/01/2014	09/29/2014
	Trifolium meduseum	NC_024166	142595	75	34	06/09/2014	09/30/2014
	Trifolium repens / common:white clover	NC_024036	132120	75	35	04/30/2014	09/29/2014
	Trifolium strictum	NC_025745	125834	75	36	01/29/2015	01/29/2015
	Trifolium subterraneum	NC_011828	144763	74	33	01/06/2009	08/06/2014
	Nicotiana tabacum / common:common tobacco	NC_001879	155943	98	45	11/18/1986	04/15/2009
	Arabidopsis thaliana / common:thale cress	NC_000932	154478	85	44	09/15/1999	03/26/201

Table S6 The PCR primers used in this study. The first two letters in each primer's ID represent the names of the adjacent scaffolds, and the gap between the scaffolds was filled by this primer. F and R represent "Forward" and "Reverse," respectively.

Primer ID	Sequence (5'-3')
AB-F	CCTTAGAGCGGACAGATACC
AB-R	CTAAGCGGGCTCACATAACA
BC-F	AAAGGGCAGAGTGAATAGAAATAC
BC-R	AAACCCGTTGCCTTACC
CD-F	AAGGGCTTATTTGATTC
CD-R	TATATGAGATTTGTCCCTACT
DE-F	ACGCAGAGATAAAGAAATAGAGAA
DE-R	TGGATCGGGGACAAACT
EF-F	TGAAAGGTCCAGTAGAGAT
EF-R	GGATGCACCCCAATGAA
FG-F	GATTCTTTCGCTGACCTATGTTGT
FG-R	CTACTTGCTTTTCTTCGGTCCAC
GA-F	CTATAGATTTACCCGTTGTTGATT
GA-R	CTTATTGATATGACCAGGAAAAA

Figure S1 Alignment of multiple *accD* protein sequences from *A. membranaceus* (Asme) and other representative legume species: *G. glabra* (Glgl), *R. pseudoacacia* (Rops), *A. americana* (Apam), *T. strictum* (Trst), *L. japonicus* (Loja), *T. glanduliferum* (Trgl), *L. luteus* (Lulu) and *I. tinctoria* (Inti).

```
                *        20         *        40         *        60         *        80         *       100         *       120         *       140
Glgl : MEKWWFNSMLFTRELEYRCGLSKSIESFGPIENTNVSEDSPIFTDMDKNIHSWK-NSENFSYSNVDYLVGIRNIRNFLSDKTFLVRDRNRKSYSIYFAIEN---QIFEIDNDHSFLNEPESFFSSYKNSRYLE--NG--S : 132
Rops : MEKWRFNSMLFNRELEYRCGLSKSMDSFGPIENTSVSEDYPILTDMDKNINSWN-DSENASYSNVDYLVGVRNIGNFLSNKAFFVRDSKRNSYSIYFTFKNLKNQILEIDNDRSFLSEPESFLSSYKNSSYWN--NG--S : 135
Apam : MEKWWFNSMLFNRELEYRCELRKSMDSLGPMENTNVSEDAQILTDMDKEIHSWN-DNDNSSYSYLDYLVDVRDIHNFLSDKTFLVRDNKRNSYSIYFDIEN---KTLEIDNDHSFLSEPESFFSSY-----LN--NV--S : 127
Trst : MEKRWFNSMLFNRGLEYRCGLSKSIDSFGPSENNNRNEDPSIFTDMDNNIHNWKTNSANSSYNHGDYLVDVRNIRNFLSDKTFLVRDRNRNNYSIYFAIEN---QILEIDNNHSFLNEPETFFSSSKNSRYLNLKNGSSS : 137
Loja : MEKWWLNSMLRNRELAYKCGLSKSTDRFGPIENTSVSEDPPILTDMDKNIHSWN-DIENCSYNNVDYLVGVENIRNFLSNKSFLVRDSKRNSYSIYFAIEN---QILEIDNDHLFLSELESFFSRYTNSIYLN--NS--S : 132
Trgl : MEKRWFNSMLFNRGLEYRCGLSKSIDSFGPIENNNINEDPSILTDMDNNIQNWKNNSENSSYNHGDYLVDVRNIRNFLSDKTFLVRDRNRNSYSIYFAIEN---QIFEIDNNHSFLNEPESFFSSSKNSSYL--KNG--Y : 133
Lulu : MEKGWFNSMLLN---MDEWGLSKSTNRFGPIENTGVSED-QILTDMDKNIHNWN-DSDNSSCSNVDYLIGVRNIRNFLSDKTFLVKDSKRNSFSIYFDIEN---PILEIDNENSFLSEPESFFYSYQNSSYLN--NV--S : 128
Inti : MEKWSFNSILFNRELEYRCGLSKSMDSFDPIENTSVSEELLILNDMDKNIHSWN-DSDNSSYSNVDYLIGVRNIQNFLSDQTFLVRDSKRNSYSIYFDIEN---QILEIDNDHSFLSESESFFSSYKNSRYLN--NV--F : 132
Asme : ---------------------------------------------MDKNIQSWH-NSANSSYSNFDYLVDGRNIRNLLSDKTFLVRDSKRKSYSIYFAIEN---NILEIDNDHSFLNEPESFFSISNNSSYLK--NG--S :  87
       mek  fns l nr l y cglsks   fgp en    ed  i tdMDknI  W  1s N Sy   DYL6  r1I NfLS1k F1V4D  Rns5SIYF ieN    ilEIDN hsFL EpE3Ffss  ns yl   N   s

                *       160         *       180         *       200         *       220         *       240         *       260         *       280
Glgl : KSDDN-HYMYDTKSNWNNNINSCIESYLRSQIGIDSYILD--DSD--------FNRYFCGKGIN-SSESK--SASIITSTNETNDSDFTKRESSSDLDETQKYKHLWIECENCYGLNYKKILKSKMNICEHCGYHLKMSS : 258
Rops : KSDDD-HSMYDTKSSWNNNINSCIENYLRYRIRIDSYILG--DSDEY--NDSYFYSYICDKVRN-SSENK--RASIITSTNDTNASDFTKRESSNDLDETQKYKHLWIECENCYGLNYKKILKSKMNICEHCGYHLKMSS : 267
Apam : KSDDD-HYMYDTQFSWKNNINSCIDSYLRSQICIDSFILG--DSDKY--NDSFFYSYIFGKGRN-CSESENFSSSIITSTNDTNDSDSAIGEGFNNLDETQKYKHLWIECENCYGLNYKKFFKSKMNICEHCGYHLKMSS : 261
Trst : KSNDH-NYMYDTKSNWNYNTNSSIGSYLGSQISIDRYILDIYSSDKY--NDGYFTSSISSKVKKNSSESK--STSIITITN---DSDFTKRESSNDLDEIEDYSHLWVQCEDCYGLNYKRFLKSKMNICEHCGYHLKMSS : 269
Loja : KGDGD-HYMYDTESSWNNNINSFIENYIRSQICIDNYILG--DGDKY--NDSYFYSYICSTIRKKSSERE--RTSIITSTNNLNDSDFTKIEGSNDLDETQKYKHLWIECENCYGLNYKKILKSKMNICEHCGYHLKMSS : 265
Trgl : KSNDH-NYMYDTKSNWNYNINSCIENYLGSQICIDSYILDIYSSDKY--NDSYFTSSICSKVK-NSSESK--STSIITIPN---GSDFTKRESFNDLDEIQDYSHLWVQCEDCYGLNYKKFLKSKMNICEHCGYHLKMSS : 264
Lulu : KIDS----MCDNKSSWNNIINSCIESYLRSRICIDSYFLS--DSDKN--NDSYLSSYICGKGIS-WSESE--SASIITSTNDTNDSDLTIRESSNDLDETQKYKHLWVECENCYGLNYKKFFKSKMNICEHCGYHLKMRS : 257
Inti : KSDDD-HYMYDSKSSWNNNINSCIESYLRSQICIDSYILG--DSDKY--NDNYFYSYICGKGRN-SSESESSSSSIITSTNYKNDSYFTRRESSKNLDETKKYKHLWIECENCYGLNYKKFLKSKMNICEHCGYHLKMSS : 266
Asme : KSNNDHYIYDIKSNWNNKINSCIESYLRSQICIDSYILG--DSVKYKYNDSDFNSYIFGKVSN-SGESK--STSIITSTNDPNAS---YLESSNDFEETQKYKHLWIECENCYGLNYKKVLKSKMNICEHCGYHLKMSS : 219
       Ks1    y6yD ks WnnniNScIe Y6rsqI IDs5iL   dsdky  nd  f syi  k    ssEs   s SIITstN  n Sd t  Essn1ldEt kYkHLW62CE1CYGLNYK4 lKSKMNICEHCGYHLKMsS

                *       300         *       320         *       340         *       360         *       380         *       400         *       420
Glgl : SDRIELSIDPGTWNPMDEDMVSVDPIEFHSEEEPYKNRIDSYQKKTGLREAVQTGTGQLNGIPVAIGIMDFQFMGGSMGSVVGEKITRLVEYASNQLLPLILVCASGGARMQEGSLSLMQMAKISSALYDYQINQKLFYV : 398
Rops : SDRIELSIDPGTWNPMDEDMVSLDPIEFHSEEEPYKDRIDSYQKMTGLTEAVQTGTGQLNGIPVAIGIMDFQFMGGSMGSVVGEKITRLVEYATNELLPLILVCASGGARMQEGSLSLMQMAKISSALYDYQINQKLFYV : 407
Apam : SDRIELSIDPGTWNPMDEDMVSLDPIEFHSEEEPYKDRIDSYQKKTGLTEAVQTGTGQLNGIPVAIGIMDFQFMGGSMGSAVGEKITRLVEYATNQLLPLILVCASGGARMQEGSLSLMQMAKISSALYDYQTNKKLFYV : 401
Trst : SDRLELLIDPGTWNPMDEYMVSLDPIEFHSEEEPY--CLDSYQERTGLLEAVQTGTGQLHGIPVAMGIMEFQFMGGSMGSVVGEKITRLIEYATNQLLPLIIVCASGGARMQEGSLSLMQMAKISSALYDYQINQKLFYV : 407
Loja : SDRIELSIDPGTWNPMDEDMISVDPIEFHSEEEPYKDRIDSYQKTTGLTEAVQTGTGHLNGIPVAIGIMDFEFMGGSMGSVVGEKITRLVEYATNQLLPLIVVCASGGARMQEGSLSLMQMAKISSALYDYQLNKKLFYV : 405
Trgl : SDRLELLIDPGTWNPMDEYMVSLDPIEFHSEEEPY--CLDSYQERTGLLEAVQTGTGQLHGIPVAIGIMEFQFMGGSMGSVVGEKITRLIEYATNQLLPLIIVCASGGARMQEGSLSLMQMAKISSALYDYQINQKLFYV : 402
Lulu : SDRIELLIDSGTWNPMDEDMVSLDPIEFHSEEDPYKDRIDSYQRKIGLTEAVQTGTGQLNGIPVAIGIMDFQFMGGSMGSVVGEKITRLVEYATNQLLPLILVCASGGARMQEGSLSLMQMAKISSALYNYQTSIKLFYV : 397
Inti : SDRIELLIDPGTWNPMDEDMISLDPIQFHSEEEPYKDRIDSYQRKTGLTEAVQTGTGQLNGIPVAIGIMDFQFMGGSMGSVVGEKITRLVEYATNQLLPLILVCASGGARMQEGSLSLMQMAKISSALHDYQTNKKLFYV : 406
Asme : SDRIELLIDPGTWNPMDEYMVSVDPIEFHSEEEPYKNRLDSYQKRTGLMEAVQTGRGQLNGIPLAIGIMDFQFMGGSMGSVVGEKITRLIEYATNQRLPLILVCASGGARMQEGSLSLMQMAKISSALYDYQINQKLFYV : 359
       SDR6EL IDpGTWNPMDE M6S6DPI2FHSEEePYk r6DSYQ  tGL EAVQTGtGqLnGIP6A6GIMdF2FMGGSMGSvVGEKITRL6EYA3N2lLPLI6VCASGGARMQEGSLSLMQMAKISSALy1YQ n KLFYV

                *       440         *       460         *       480         *       500         *       520
Glgl : SILTSPTTGGVTASFGMLGDIIIAEPNAYIAFAGKRVIEQTLNKEVPEGSQSAEFLFQKGLFDSIVPRNPLKEVLSELFQLHAFFPLTQNEKSSRA---------- : 494
Rops : SILTSPTTGGVTASFGMLGDIIIAEPNAYIAFAGKRVIEQTLNKAVPEGSQAAEYLFHKGLFDSIVPRNPLKGVLSDLFQLHAFFPLTQNEKSSRT---------- : 503
Apam : SILTSPTTGGVTASFGMLGDIIIAEPNAYIAFAGKRVIEQTLNKAVPEGSQGAEYLFHKGLFDSIVPRNLLKGVLIELFQFHNFFSLT------------------ : 489
Trst : PILTSPTTGGVTASFGMLGDIIIAEPDATIAFAGKRVIEETLNKEVPEGSQSAEFLFEKGLFDSIVPRNLLKEVLSELFQLHGFFPLIQNEK-------------- : 499
Loja : SILTSPTTGGVTASFGMLGDIIIAEPNAYIAFAGKRVIEQTLNKAVPEGSQAAEYLFHKGLFDSIVPRNPLKGILSELFQLHAFFPLIQNEKESRS---------- : 501
Trgl : PILTSPTTGGVTASFGMLGDIIIAEPDATIAFAGKRVIEETLNKEVPEGSQSAEFLFEKGLFDSIVPRNLLKEVLSELFQLHGFFPFDSK---------------- : 492
Lulu : SILTSPTTGGVTASFGMLGDIIIAEPNAYIAFAGKRVIEQTLNKTVPEGSQAAEYLFHKGLFDSIIPRNPLKQVLSELFHLHGFFPLTQNEKSTSRALNYF----- : 498
Inti : SILTSPTTGGVTASFGMLGDIIIAEPNAYIAFAGKRVIEQTLNKAIPDGSQAAEYLFHKGLFDSIVPRNPLKGVLSELFQLHAFFSLTKNEKNYETKIKLEHTRAK : 512
Asme : PILTSPTTGGVTASFGMLGDIIIAEPNAYIAFAGKRVIEQTLNKEVPEGSQSAEFLFQKGLFDSIVPRNLLKEVLTELFQFHGFVPLTQNEK-------------- : 451
        ILTSPTTGGVTASFGMLGDIIIAEP1AyIAFAGKRVIE2TLNK 6PeGSQ AE5LF KGLFDSI6PRN LK 6L eLFqlH Ffpl  nek
```

Figure S2 PCR amplification and Sanger sequencing results for the highly polymorphic regions in the *A. membranaceus* genome. Panels A, B, and C shows the three-gap regions between scaffolds, namely, A and B, B and C, and D and E respectively. The left side of each panel shows the agarose gel electrophoresis result, whereas the right side shows the trace files indicating that multiple sequences were obtained from the same PCR bands.

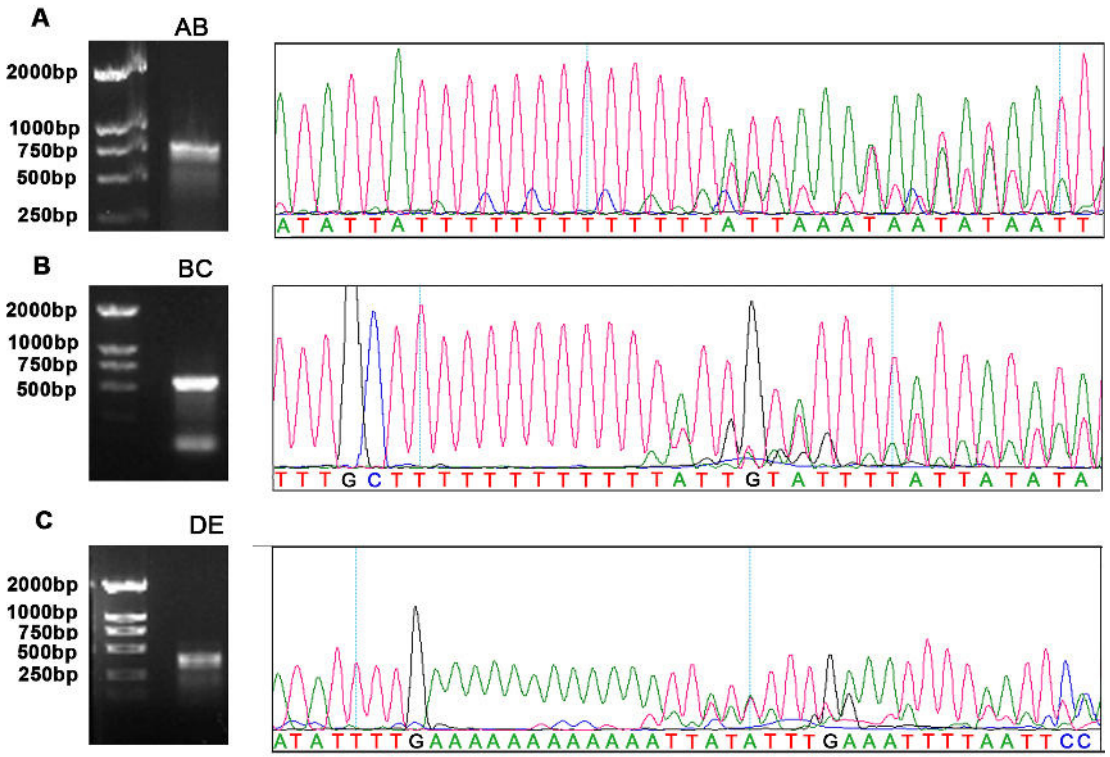

Figure S3 Alignment of the PCR products for the highly polymorphic regions in the *A. membranaceus* genome. All the sequences provided in this file were considered high quality as no overlapping peaks were observed. Panels A, B and C represent the regions AB, BC and DE respectively.

(A) Region AB

```
                            *        20         *        40         *        60         *        80         *       100         *
AB           : CCTTAGAGCGGACAGATACCGACCCTATCATTCTAATATAATATATAATAACAAAACTAAAAGCAAAAATCCATTTATTAATTTCGAATTCGATAGAAATATCGAATTTC : 110
AB-i1-c4-F   : CCTTAGAGCGGACAGATACCGACCCTATCATTCTAATATAATATATAATAACAAAACTAAAAGCAAAAATCCATTTATTAATTTCGAATTCGATAGAAATATCGAATTTC : 110
AB-i1-c4-R   : CCTTAGAGCGGACAGATACCGACCCTATCATTCTAATATAATATATAATAACAAAACTAAAAGCAAAAATCCATTTATTAATTTCGAATTCGATAGAAATATCGAATTTC : 110
AB-i1-c11-F  : CCTTAGAGCGGACAGATACCGACCCTATCATTCTAATATAATATATAATAACAAAACTAAAAGCAAAAATCCATTTATTAATTTCGAATTCGATAGAAATATCGAATTTC : 110
AB-i1-c11-R  : CCTTAGAGCGGACAGATACCGACCCTATCATTCTAATATAATATATAATAACAAAACTAAAAGCAAAAATCCATTTATTAATTTCGAATTCGATAGAAATATCGAATTTC : 110
AB-i1-c14-F  : CCTTAGAGCGGACAGATACCGACCCTATCATTCTAATATAATATATAATAACAAAACTAAAAGCAAAAATCCATTTATTAATTTCGAATTCGATAGAAATATCGAATTTC : 110
AB-i1-c14-R  : CCTTAGAGCGGACAGATACCGACCCTATCATTCTAATATAATATATAATAACAAAACTAAAAGCAAAAATCCATTTATTAATTTCGAATTCGATAGAAATATCGAATTTC : 110
AB-i1-c15-F  : CCTTAGAGCGGACAGATACCGACCCTATCATTCTAATATAATATATAATAACAAAACTAAAAGCAAAAATCTATTTATTAATTTCGAATTCGATAGAAATATCGAATTTC : 110
AB-i1-c15-R  : CCTTAGAGCGGACAGATACCGACCCTATCATTCTAATATAATATATAATAACAAAACTAAAAGCAAAAATCTATTTATTAATTTCGAATTCGATAGAAATATCGAATTTC : 110
AB-i1-c16-F  : CCTTAGAGCGGACAGATACCGACCCTATCATTCTAATATAATATATAATAACAAAACTAAAAGCAAAAATCCATTTATTAATTTCGAATTCGATAGAAATATCGAATTTC : 110
AB-i1-c16-R  : CCTTAGAGCGGACAGATACCGACCCTATCATTCTAATATAATATATAATAACAAAACTAAAAGCAAAAATCCATTTATTAATTTCGAATTCGATAGAAATATCGAATTTC : 110
AB-i1-c17-F  : CCTTAGAGCGGACAGATACCGACCCTATCATTCTAATATAATATATAATAACAAAACTAAAAGCAAAAATCCATTTATTAATTTCGAATTCGATAGAAATATCGAATTTC : 110
AB-i1-c17-R  : CCTTAGAGCGGACAGATACCGACCCTATCATTCTAATATAATATATAATAACAAAACTAAAAGCAAAAATCCATTTATTAATTTCGAATTCGATAGAAATATCGAATTTC : 110
AB-i1-c18-F  : -CCTAGAGCGGACAGATACCGACCCTATCATTCTAATATAATATATAATAACAAAACTAAAAGCAAAAATCCATTTATTAATTTCGAATTCGATAGAAATATCGAATTTC : 109
AB-i1-c18-R  : -CCTAGAGCGGACAGATACCGACCCTATCATTCTAATATAATATATAATAACAAAACTAAAAGCAAAAATCCATTTATTAATTTCGAATTCGATAGAAATATCGAATTTC : 109
AB-i1-c19-F  : CCTTAGAGCGGACAGATACCGACCCTATCATTCTAATATAATATATAATAACAAAACTAAAAGCAAAAATCCATTTATTAATTTCGAATTCGATAGAAATATCGAATTTC : 110
AB-i1-c19-R  : CCTTAGAGCGGACAGATACCGACCCTATCATTCTAATATAATATATAATAACAAAACTAAAAGCAAAAATCCATTTATTAATTTCGAATTCGATAGAAATATCGAATTTC : 110
AB-i5-c1-F   : CCTTAGAGCGGACAGATACCGACCCTATCATTCTAATATAATATATAATAACAAAACTAAAAGCAAAAATCCATTTATTAATTTCGAATTCGATAGAAATATCGAATTTC : 110
AB-i5-c1-R   : CCTTAGAGCGGACAGATACCGACCCTATCATTCTAATATAATATATAATAACAAAACTAAAAGCAAAAATCCATTTATTAATTTCGAATTCGATAGAAATATCGAATTTC : 110
AB-i5-c2-F   : CCTTAGAGCGGACAGATACCGACCCTATCATTCTAATATAATATATAATAACAAAACTAAAAGCAAAAATCCATTTATTAATTTCGAATTCGATAGAAATATCGAATTTC : 110
AB-i5-c2-R   : CCTTAGAGCGGACAGATACCGACCCTATCATTCTAATATAATATATAATAACAAAACTAAAAGCAAAAATCCATTTATTAATTTCGAATTCGATAGAAATATCGAATTTC : 110
AB-i5-c6-F   : CCTTAGAGCGGACAGATACCGACCCTATCATTCTAATATAATATATAATAACAAAACTAAAAGCAAAAATCCATTTATTAATTTCGAATTCGATAGAAATATCGAATTTC : 110
AB-i5-c6-R   : CCTTAGAGCGGACAGATACCGACCCTATCATTCTAATATAATATATAATAACAAAACTAAAAGCAAAAATCCATTTATTAATTTCGAATTCGATAGAAATATCGAATTTC : 110
AB-i5-c7-F   : CCTTAGAGCGGACAGATACCGACCCTATCATTCTAATATAATATATAATAACAAAACTAAAAGCAAAAATCCATTTATTAATTTCGAATTCGATAGAAATATCGAATTTC : 110
AB-i5-c7-R   : CCTTAGAGCGGACAGATACCGACCCTATCATTCTAATATAATATATAATAACAAAACTAAAAGCAAAAATCCATTTATTAATTTCGAATTCGATAGAAATATCGAATTTC : 110
AB-i5-c11-F  : CCTTAGAGCGGACAGATACCGACCCTATCATTCTAATATAATATATAATAACAAAACTAAAAGCAAAAATCCATTTATTAATTTCGAATTCGATAGAAATATCGAATTTC : 110
AB-i5-c11-R  : CCTTAGAGCGGACAGATACCGACCCTATCATTCTAATATAATATATAATAACAAAACTAAAAGCAAAAATCCATTTATTAATTTCGAATTCGATAGAAATATCGAATTTC : 110
AB-i5-c12-F  : CCTTAGAGCGGACAGATACCGACCCTATCATTCTAATATAATATATAATAACAAAACTAAAAGCAAAAATCCATTTATTAATTTCGAATTCGATAGAAATATCGAATTTC : 110
AB-i5-c12-R  : CCTTAGAGCGGACAGATACCGACCCTATCATTCTAATATAATATATAATAACAAAACTAAAAGCAAAAATCCATTTATTAATTTCGAATTCGATAGAAATATCGAATTTC : 110
AB-i5-c13-F  : -CTTAGAGCGGACAGATACCGACCCTATCATTCTAATATAATATATAATAACAAAACTAAAAGCAAAAATCCATTTATTAATTTCGAATTCGATAGAAATATCGAATTTC : 109
AB-i5-c13-R  : -CTTAGAGCGGACAGATACCGACCCTATCATTCTAATATAATATATAATAACAAAACTAAAAGCAAAAATCCATTTATTAATTTCGAATTCGATAGAAATATCGAATTTC : 109
AB-i5-c14-F  : CCTTAGAGCGGACAGATACCGACCCTATCATTCTAATATAATATATAATAACAAAACTAAAAGCAAAAATCCATTTATTAATTTCGAATTCGATAGAAATATCGAATTTC : 110
AB-i5-c14-R  : CCTTAGAGCGGACAGATACCGACCCTATCATTCTAATATAATATATAATAACAAAACTAAAAGCAAAAATCCATTTATTAATTTCGAATTCGATAGAAATATCGAATTTC : 110
AB-i5-c15-F  : CCTTAGAGCGGACAGATACCGACCCTATCATTCTAATATAATATATAATAACAAAACTAAAAGCAAAAATCCATTTATTAATTTCGAATTCGATAGAAATATCGAATTTC : 110
AB-i5-c15-R  : CCTTAGAGCGGACAGATACCGACCCTATCATTCTAATATAATATATAATAACAAAACTAAAAGCAAAAATCCATTTATTAATTTCGAATTCGATAGAAATATCGAATTTC : 110
```

```
AB-i6-c4-F  : CCTTAGAGCGGACAGATACCGACCCTATCATTCTAATATAATATATAATAACAAAACTAAAAGCAAAAATCCATTTATTAATTTCGAATTCGATAGAAATATCGAATTTC : 110
AB-i6-c4-R  : CCTTAGAGCGGACAGATACCGACCCTATCATTCTAATATAATATATAATAACAAAACTAAAAGCAAAAATCCATTTATTAATTTCGAATTCGATAGAAATATCGAATTTC : 110
AB-i6-c7-F  : CCTTAGAGCGGACAGATACCGACCCTATCATTCTAATATAATATATAATAACAAAACTAAAAGCAAAAATCCATTTATTAATTTCGAATTCGATAGAAATATCGAATTTC : 110
AB-i6-c7-R  : CCTTAGAGCGGACAGATACCGACCCTATCATTCTAATATAATATATAATAACAAAACTAAAAGCAAAAATCCATTTATTAATTTCGAATTCGATAGAAATATCGAATTTC : 110
AB-i6-c8-F  : CCTTAGAGCGGACAGATACCGACCCTATCATTCTAATATAATATATAATAACAAAACTAAAAGCAAAAATCCATTTATTAATTTCGAATTCGATAGAAATATCGAATTTC : 110
AB-i6-c8-R  : CCTTAGAGCGGACAGATACCGACCCTATCATTCTAATATAATATATAATAACAAAACTAAAAGCAAAAATCCATTTATTAATTTCGAATTCGATAGAAATATCGAATTTC : 110
AB-i6-c9-F  : CCTTAGAGCGGACAGATACCGACCCTATCATTCTAATATAATATATAATAACAAAACTAAAAGCAAAAATCCATTTATTAATTTCGAATTCGATAGAAATATCGAATTTC : 110
AB-i6-c9-R  : CCTTAGAGCGGACAGATACCGACCCTATCATTCTAATATAATATATAATAACAAAACTAAAAGCAAAAATCCATTTATTAATTTCGAATTCGATAGAAATATCGAATTTC : 110
AB-i6-c11-F : --TTAGAGCGGACAGATACCGACCCTATCATTCTAATATAATATATAATAACAAAACTAAAAGCAAAAATCCATTTATTAATTTCGAATTCGATAGAAATATCGAATTTC : 108
AB-i6-c11-R : --TTAGAGCGGACAGATACCGACCCTATCATTCTAATATAATATATAATAACAAAACTAAAAGCAAAAATCCATTTATTAATTTCGAATTCGATAGAAATATCGAATTTC : 108
AB-i6-c12-F : CCTTAGAGCGGACAGATACCGACCCTATCATTCTAATATAATATATAATAACAAAACTAAAAGCAAAAATCCATTTATTAATTTCGAATTCGATAGAAATATCGAATTTC : 110
AB-i6-c12-R : CCTTAGAGCGGACAGATACCGACCCTATCATTCTAATATAATATATAATAACAAAACTAAAAGCAAAAATCCATTTATTAATTTCGAATTCGATAGAAATATCGAATTTC : 110
AB-i6-c17-F : CCTTAGAGCGGACAGATACCGACCCTATCATTCTAATATAATATATAATAACAAAACTAAAAGCAAAAATCCATTTATTAATTTCGAATTCGATAGAAATATCGAATTTC : 110
AB-i6-c17-R : CCTTAGAGCGGACAGATACCGACCCTATCATTCTAATATAATATATAATAACAAAACTAAAAGCAAAAATCCATTTATTAATTTCGAATTCGATAGAAATATCGAATTTC : 110
AB-i7-c11-F : CCTTAGAGCGGACAGATACCGACCCTATCATTCTAATATAATATATAATAACAAAACTAAAAGCAAAAATCCATTTATTAATTTCGAATTCGATAGAAATATCGAATTTC : 110
AB-i7-c11-R : CCTTAGAGCGGACAGATACCGACCCTATCATTCTAATATAATATATAATAACAAAACTAAAAGCAAAAATCCATTTATTAATTTCGAATTCGATAGAAATATCGAATTTC : 110
AB-i7-c12-F : CCTTAGAGCGGACAGATACCGACCCTATCATTCTAATATAATATATAATAACAAAACTAAAAGCAAAAATCCATTTATTAATTTCGAATTCGATAGAAATATCGAATTTC : 110
AB-i7-c12-R : CCTTAGAGCGGACAGATACCGACCCTATCATTCTAATATAATATATAATAACAAAACTAAAAGCAAAAATCCATTTATTAATTTCGAATTCGATAGAAATATCGAATTTC : 110
AB-i7-c13-F : CCTTAGAGCGGACAGATACCGACCCTATCATTCTAATATAATATATAATAACAAAACTAAAAGCAAAAATCCATTTATTAATTTCGAATTCGATAGAAATATCGAATTTC : 110
AB-i7-c13-R : CCTTAGAGCGGACAGATACCGACCCTATCATTCTAATATAATATATAATAACAAAACTAAAAGCAAAAATCCATTTATTAATTTCGAATTCGATAGAAATATCGAATTTC : 110
AB-i7-c14-F : CCTTAGAGCGGACAGATACCGACCCTATCATTCTAATATAATATATAATAACAAAACTAAAAGCAAAAATCCATTTATTAATTTCGAATTCGATAGAAATATCGAATTTC : 110
AB-i7-c14-R : CCTTAGAGCGGACAGATACCGACCCTATCATTCTAATATAATATATAATAACAAAACTAAAAGCAAAAATCCATTTATTAATTTCGAATTCGATAGAAATATCGAATTTC : 110
AB-i7-c16-F : CCTTAGAGCGGACAGATACCGACCCTATCATTCTAATATAATATATAATAACAAAACTAAAAGCAAAAATCCATTTATTAATTTCGAATTCGATAGAAATATCGAATTTC : 110
AB-i7-c16-R : CCTTAGAGCGGACAGATACCGACCCTATCATTCTAATATAATATATAATAACAAAACTAAAAGCAAAAATCCATTTATTAATTTCGAATTCGATAGAAATATCGAATTTC : 110
AB-i7-c18-F : CCTTAGAGCGGACAGATACCGACCCTATCATTCTAATATAATATATAATAACAAAACTAAAAGCAAAAATCCATTTATTAATTTCGAATTCGATAGAAATATCGAATTTC : 110
AB-i7-c18-R : CCTTAGAGCGGACAGATACCGACCCTATCATTCTAATATAATATATAATAACAAAACTAAAAGCAAAAATCCATTTATTAATTTCGAATTCGATAGAAATATCGAATTTC : 110
AB-i7-c19-F : -CTTAGAGCGGACAGATACCGACCCTATCATTCTAATATAATATATAATAACAAAACTAAAAGCAAAAATCCATTTATTAATTTCGAATTCGATAGAAATATCGAATTTC : 109
AB-i7-c19-R : -CTTAGAGCGGACAGATACCGACCCTATCATTCTAATATAATATATAATAACAAAACTAAAAGCAAAAATCCATTTATTAATTTCGAATTCGATAGAAATATCGAATTTC : 109
AB-i7-c20-F : CCTTAGAGCGGACAGATACCGACCCTATCATTCTAATATAATATATAATAACAAAACTAAAAGCAAAAATCCATTTATTAATTTCGAATTCGATAGAAATATCGAATTTC : 110
AB-i7-c20-R : CCTTAGAGCGGACAGATACCGACCCTATCATTCTAATATAATATATAATAACAAAACTAAAAGCAAAAATCCATTTATTAATTTCGAATTCGATAGAAATATCGAATTTC : 110
              cctTAGAGCGGACAGATACCGACCCTATCATTCTAATATAATATATAATAACAAAACTAAAAGCAAAAATCcATTTATTAATTTCGAATTCGATAGAAATATCGAATTTC

                    120         *       140         *       160         *       180         *       200         *       220
AB          : TAATTACTTATTTATATATTTTAATTTAATTTCTTTTTATAATCCATCCAATTTTTTAGAATTCTAATGTAGAATTCTATTCTATACATTATATTACATTATATTATTTT : 220
AB-i1-c4-F  : TAATTACTTATTTATATATTTTAATTTAATTTCTTTTTATAATCCATCCAATTTTTTAGAATTCTAATGTAGAATTCTATTCTATACATTATATTACATTATATTATTTT : 220
AB-i1-c4-R  : TAATTACTTATTTATATATTTTAATTTAATTTCTTTTTATAATCCATCCAATTTTTTAGAATTCTAATGTAGAATTCTATTCTATACATTATATTACATTATATTATTTT : 220
AB-i1-c11-F : TAATTACTTATTTATATATTTTAATTTAATTTCTTTTTATAATCCATCCAATTTTTTAGAATTCTAATGTAGAATTCTATTCTATACATTATATTACATTATATTATTTT : 220
AB-i1-c11-R : TAATTACTTATTTATATATTTTAATTTAATTTCTTTTTATAATCCATCCAATTTTTTAGAATTCTAATGTAGAATTCTATTCTATACATTATATTACATTATATTATTTT : 220
AB-i1-c14-F : TAATTACTTATTTATATATTTTAATTTAATTTCTTTTTATAATCCATCCAATTTTTTAGAATTCTAATGTAGAATTCTATTCTATACATTATATTACATTATATTATTTT : 220
AB-i1-c14-R : TAATTACTTATTTATATATTTTAATTTAATTTCTTTTTATAATCCATCCAATTTTTTAGAATTCTAATGTAGAATTCTATTCTATACATTATATTACATTATATTATTTT : 220
AB-i1-c15-F : TAATTACTTATTTATATATTTTAATTTAATTTCTTTTTATAATCCATCCAATTTTTTAGAATTCTAATGTAGAATTCTATTCTATACATTATATTACATTATATTATTTT : 220
AB-i1-c15-R : TAATTACTTATTTATATATTTTAATTTAATTTCTTTTTATAATCCATCCAATTTTTTAGAATTCTAATGTAGAATTCTATTCTATACATTATATTACATTATATTATTTT : 220
AB-i1-c16-F : TAATTACTTATTTATATATTTTAATTTAATTTCTTTTTATAATCCATCCAATTTTTTAGAATTCTAATGTAGAATTCTATTCTATACATTATATTACATTATATTATTTT : 220
AB-i1-c16-R : TAATTACTTATTTATATATTTTAATTTAATTTCTTTTTATAATCCATCCAATTTTTTAGAATTCTAATGTAGAATTCTATTCTATACATTATATTACATTATATTATTTT : 220
AB-i1-c17-F : TAATTACTTATTTATATATTTTAATTTAATTTCTTTTTATAATCCATCCAATTTTTTAGAATTCTAATGTAGAATTCTATTCTATACATTATATTACATTATATTATTTT : 220
AB-i1-c17-R : TAATTACTTATTTATATATTTTAATTTAATTTCTTTTTATAATCCATCCAATTTTTTAGAATTCTAATGTAGAATTCTATTCTATACATTATATTACATTATATTATTTT : 220
```

AB-i1-c18-F	TAATTACTTATTTATATATTTTAATTTAATTTCTTTTTATAATCCATCCAATTTTTTAGAATTCTAATGTAGAATTCTATTCTATACATTATATTACATTATATTATTTT	219
AB-i1-c18-R	TAATTACTTATTTATATATTTTAATTTAATTTCTTTTTATAATCCATCCAATTTTTTAGAATTCTAATGTAGAATTCTATTCTATACATTATATTACATTATATTATTTT	219
AB-i1-c19-F	TAATTACTTATTTATATATTTTAATTTAATTTCTTTTTATAATCCATCCAATTTTTTAGAATTCTAATGTAGAATTCTATTCTATACATTATATTACATTATATTATTTT	220
AB-i1-c19-R	TAATTACTTATTTATATATTTTAATTTAATTTCTTTTTATAATCCATCCAATTTTTTAGAATTCTAATGTAGAATTCTATTCTATACATTATATTACATTATATTATTTT	220
AB-i5-c1-F	TAATTACTTATTTATATATTTTAATTTAATTTCTTTTTATAATCCATCCAATTTTTTAGAATTCTAATGTAGAATTCTATTCTATACATTATATTACATTATATTATTTT	220
AB-i5-c1-R	TAATTACTTATTTATATATTTTAATTTAATTTCTTTTTATAATCCATCCAATTTTTTAGAATTCTAATGTAGAATTCTATTCTATACATTATATTACATTATATTATTTT	220
AB-i5-c2-F	TAATTACTTATTTATATATTTTAATTTAATTTCTTTTTATAATCCATCCAATTTTTTAGAATTCTAATGTAGAATTCTATTCTATACATTATATTACATTATATTATTTT	220
AB-i5-c2-R	TAATTACTTATTTATATATTTTAATTTAATTTCTTTTTATAATCCATCCAATTTTTTAGAATTCTAATGTAGAATTCTATTCTATACATTATATTACATTATATTATTTT	220
AB-i5-c6-F	TAATTACTTATTTATATATTTTAATTTAATTTCTTTTTATAATCCATCCAATTTTTTAGAATTCTAATGTAGAATTCTATTCTATACATTATATTACATTATATTATTTT	220
AB-i5-c6-R	TAATTACTTATTTATATATTTTAATTTAATTTCTTTTTATAATCCATCCAATTTTTTAGAATTCTAATGTAGAATTCTATTCTATACATTATATTACATTATATTATTTT	220
AB-i5-c7-F	TAATTACTTATTTATATATTTTAATTTAATTTCTTTTTATAATCCATCCAATTTTTTAGAATTCTAATGTAGAATTCTATTCTATACATTATATTACATTATATTATTTT	220
AB-i5-c7-R	TAATTACTTATTTATATATTTTAATTTAATTTCTTTTTATAATCCATCCAATTTTTTAGAATTCTAATGTAGAATTCTATTCTATACATTATATTACATTATATTATTTT	220
AB-i5-c11-F	TAATTACTTATTTATATATTTTAATTTAATTTCTTTTTATAATCCATCCAATTTTTTAGAATTCTAATGTAGAATTCTATTCTATACATTATATTACATTATATTATTTT	220
AB-i5-c11-R	TAATTACTTATTTATATATTTTAATTTAATTTCTTTTTATAATCCATCCAATTTTTTAGAATTCTAATGTAGAATTCTATTCTATACATTATATTACATTATATTATTTT	220
AB-i5-c12-F	TAATTACTTATTTATATATTTTAATTTAATTTCTTTTTATAATCCATCCAATTTTTTAGAATTCTAATGTAGAATTCTATTCTATACATTATATTACATTATATTATTTT	220
AB-i5-c12-R	TAATTACTTATTTATATATTTTAATTTAATTTCTTTTTATAATCCATCCAATTTTTTAGAATTCTAATGTAGAATTCTATTCTATACATTATATTACATTATATTATTTT	220
AB-i5-c13-F	TAATTACTTATTTATATATTTTAATTTAATTTCTTTTTATAATCCATCCAATTTTTTAGAATTCTAATGTAGAATTCTATTCTATACATTATATTACATTATATTATTTT	219
AB-i5-c13-R	TAATTACTTATTTATATATTTTAATTTAATTTCTTTTTATAATCCATCCAATTTTTTAGAATTCTAATGTAGAATTCTATTCTATACATTATATTACATTATATTATTTT	219
AB-i5-c14-F	TAATTACTTATTTATATATTTTAATTTAATTTCTTTTTATAATCCATCCAATTTTTTAGAATTCTAATGTAGAATTCTATTCTATACATTATATTACATTATATTATTTT	220
AB-i5-c14-R	TAATTACTTATTTATATATTTTAATTTAATTTCTTTTTATAATCCATCCAATTTTTTAGAATTCTAATGTAGAATTCTATTCTATACATTATATTACATTATATTATTTT	220
AB-i5-c15-F	TAATTACTTATTTATATATTTTAATTTAATTTCTTTTTATAATCCATCCAATTTTTTAGAATTCTAATGTAGAATTCTATTCTATACATTATATTACATTATATTATTTT	220
AB-i5-c15-R	TAATTACTTATTTATATATTTTAATTTAATTTCTTTTTATAATCCATCCAATTTTTTAGAATTCTAATGTAGAATTCTATTCTATACATTATATTACATTATATTATTTT	220
AB-i6-c4-F	TAATTACTTATTTATATATTTTAATTTAATTTCTTTTTATAATCCATCCAATTTTTTAGAATTCTAATGTAGAATTCTATTCTATACATTATATTACATTATATTATTTT	220
AB-i6-c4-R	TAATTACTTATTTATATATTTTAATTTAATTTCTTTTTATAATCCATCCAATTTTTTAGAATTCTAATGTAGAATTCTATTCTATACATTATATTACATTATATTATTTT	220
AB-i6-c7-F	TAATTACTTATTTATATATTTTAATTTAATTTCTTTTTATAATCCATCCAATTTTTTAGAATTCTAATGTAGAATTCTATTCTATACATTATATTACATTATATTATTTT	220
AB-i6-c7-R	TAATTACTTATTTATATATTTTAATTTAATTTCTTTTTATAATCCATCCAATTTTTTAGAATTCTAATGTAGAATTCTATTCTATACATTATATTACATTATATTATTTT	220
AB-i6-c8-F	TAATTACTTATTTATATATTTTAATTTAATTTCTTTTTATAATCCATCCAATTTTTTAGAATTCTAATGTAGAATTCTATTCTATACATTATATTACATTATATTATTTT	220
AB-i6-c8-R	TAATTACTTATTTATATATTTTAATTTAATTTCTTTTTATAATCCATCCAATTTTTTAGAATTCTAATGTAGAATTCTATTCTATACATTATATTACATTATATTATTTT	220
AB-i6-c9-F	TAATTACTTATTTATATATTTTAATTTAATTTCTTTTTATAATCCATCCAATTTTTTAGAATTCTAATGTAGAATTCTATTCTATACATTATATTACATTATATTATTTT	220
AB-i6-c9-R	TAATTACTTATTTATATATTTTAATTTAATTTCTTTTTATAATCCATCCAATTTTTTAGAATTCTAATGTAGAATTCTATTCTATACATTATATTACATTATATTATTTT	220
AB-i6-c11-F	TAATTACTTATTTATATATTTTAATTTAATTTCTTTTTATAATCCATCCAATTTTTTAGAATTCTAATGTAGAATTCTATTCTATACATTATATTACATTATATTATTTT	218
AB-i6-c11-R	TAATTACTTATTTATATATTTTAATTTAATTTCTTTTTATAATCCATCCAATTTTTTAGAATTCTAATGTAGAATTCTATTCTATACATTATATTACATTATATTATTTT	218
AB-i6-c12-F	TAATTACTTATTTATATATTTTAATTTAATTTCTTTTTATAATCCATCCAATTTTTTAGAATTCTAATGTAGAATTCTATTCTATACATTATATTACATTATATTATTTT	220
AB-i6-c12-R	TAATTACTTATTTATATATTTTAATTTAATTTCTTTTTATAATCCATCCAATTTTTTAGAATTCTAATGTAGAATTCTATTCTATACATTATATTACATTATATTATTTT	220
AB-i6-c17-F	TAATTACTTATTTATATATTTTAATTTAATTTCTTTTTATAATCCATCCAATTTTTTAGAATTCTAATGTAGAATTCTATTCTATACATTATATTACATTATATTATTTT	220
AB-i6-c17-R	TAATTACTTATTTATATATTTTAATTTAATTTCTTTTTATAATCCATCCAATTTTTTAGAATTCTAATGTAGAATTCTATTCTATACATTATATTACATTATATTATTTT	220
AB-i7-c11-F	TAATTACTTATTTATATATTTTAATTTAATTTCTTTTTATAATCCATCCAATTTTTTAGAATTCTAATGTAGAATTCTATTCTATACATTATATTACATTATATTATTTT	220
AB-i7-c11-R	TAATTACTTATTTATATATTTTAATTTAATTTCTTTTTATAATCCATCCAATTTTTTAGAATTCTAATGTAGAATTCTATTCTATACATTATATTACATTATATTATTTT	220
AB-i7-c12-F	TAATTACTTATTTATATATTTTAATTTAATTTCTTTTTATAATCCATCCAATTTTTTAGAATTCTAATGTAGAATTCTATTCTATACATTATATTACATTATATTATTTT	220
AB-i7-c12-R	TAATTACTTATTTATATATTTTAATTTAATTTCTTTTTATAATCCATCCAATTTTTTAGAATTCTAATGTAGAATTCTATTCTATACATTATATTACATTATATTATTTT	220
AB-i7-c13-F	TAATTACTTATTTATATATTTTAATTTAATTTCTTTTTATAATCCATCCAATTTTTTAGAATTCTAATGTAGAATTCTATTCTATACATTATATTACATTATATTATTTT	220
AB-i7-c13-R	TAATTACTTATTTATATATTTTAATTTAATTTCTTTTTATAATCCATCCAATTTTTTAGAATTCTAATGTAGAATTCTATTCTATACATTATATTACATTATATTATTTT	220
AB-i7-c14-F	TAATTACTTATTTATATATTTTAATTTAATTTCTTTTTATAATCCATCCAATTTTTTAGAATTCTAATGTAGAATTCTATTCTATACATTATATTACATTATATTATTTT	220
AB-i7-c14-R	TAATTACTTATTTATATATTTTAATTTAATTTCTTTTTATAATCCATCCAATTTTTTAGAATTCTAATGTAGAATTCTATTCTATACATTATATTACATTATATTATTTT	220
AB-i7-c16-F	TAATTACTTATTTATATATTTTAATTTAATTTCTTTTTATAATCCATCCAATTTTTTAGAATTCTAATGTAGAATTCTATTCTATACATTATATTACATTATATTATTTT	220
AB-i7-c16-R	TAATTACTTATTTATATATTTTAATTTAATTTCTTTTTATAATCCATCCAATTTTTTAGAATTCTAATGTAGAATTCTATTCTATACATTATATTACATTATATTATTTT	220

```
AB-i7-c18-F : TAATTACTTATTTATATATTTTAATTTAATTTCTTTTTATAATCCATCCAATTTTTTAGAATTCTAATGTAGAATTCTATTCTATACATTATATTACATTATATTATTTT : 220
AB-i7-c18-R : TAATTACTTATTTATATATTTTAATTTAATTTCTTTTTATAATCCATCCAATTTTTTAGAATTCTAATGTAGAATTCTATTCTATACATTATATTACATTATATTATTTT : 220
AB-i7-c19-F : TAATTACTTATTTATATATTTTAATTTAATTTCTTTTTATAATCCATCCAATTTTTTAGAATTCTAATGTAGAATTCTATTCTATACATTATATTACATTATATTATTTT : 219
AB-i7-c19-R : TAATTACTTATTTATATATTTTAATTTAATTTCTTTTTATAATCCATCCAATTTTTTAGAATTCTAATGTAGAATTCTATTCTATACATTATATTACATTATATTATTTT : 219
AB-i7-c20-F : TAATTACTTATTTATATATTTTAATTTAATTTCTTTTTATAATCCATCCAATTTTTTAGAATTCTAATGTAGAATTCTATTCTATACATTATATTACATTATATTATTTT : 220
AB-i7-c20-R : TAATTACTTATTTATATATTTTAATTTAATTTCTTTTTATAATCCATCCAATTTTTTAGAATTCTAATGTAGAATTCTATTCTATACATTATATTACATTATATTATTTT : 220
              TAATTACTTATTTATATATTTTAATTTAATTTCTTTTTATAATCCATCCAATTTTTTAGAATTCTAATGTAGAATTCTATTCTATACATTATATTACATTATATTATTTT

                      *       240         *       260         *       280         *       300         *       320         *
AB          : TTTTTTTTT-TATTAAATAATATAATTTAATAATTGTCCTGTAATCTAATTATTCATTATAATATTATTTTCAATTTGAAATCTAATCTACTACTATTAGTCATTATTTCA : 329
AB-i1-c4-F  : TTTTTTTTT-TATTAAATAATATAATTTAATAATTGTCCTGTAATCTAATTATTCATTATAATATTATTTTCAATTTGAAATCTAATCTACTACTATTAGTCATTATTTCA : 329
AB-i1-c4-R  : TTTTTTTTT-TATTAAATAATATAATTTAATAATTGTCCTGTAATCTAATTATTCATTATAATATTATTTTCAATTTGAAATCTAATCTACTACTATTAGTCATTATTTCA : 329
AB-i1-c11-F : TTTTTTTTT-TATTAAATAATATAATTTAATAATTGTCCTGTAATCTAATTATTCATTATAATATTATTTTCAATTTGAAATCTAATCTACTACTATTAGTCATTATTTCA : 329
AB-i1-c11-R : TTTTTTTTT-TATTAAATAATATAATTTAATAATTGTCCTGTAATCTAATTATTCATTATAATATTATTTTCAATTTGAAATCTAATCTACTACTATTAGTCATTATTTCA : 329
AB-i1-c14-F : TTTTTTTTT-TATTAAATAATATAATTTAATAATTGTCCTGTAATCTAATTATTCATTATAATATTATTTTCAATTTGAAATCTAATCTACTACTATTAGTCATTATTTCA : 329
AB-i1-c14-R : TTTTTTTTT-TATTAAATAATATAATTTAATAATTGTCCTGTAATCTAATTATTCATTATAATATTATTTTCAATTTGAAATCTAATCTACTACTATTAGTCATTATTTCA : 329
AB-i1-c15-F : TTTTTTTTT-TATTAAATAATATAATTTAATAATTGTCCTGTAATCTAATTATTCATTATAATATTATTTTCAATTTGAAATCTAATCTACTACTATTAGTCATTATTTCA : 329
AB-i1-c15-R : TTTTTTTTT-TATTAAATAATATAATTTAATAATTGTCCTGTAATCTAATTATTCATTATAATATTATTTTCAATTTGAAATCTAATCTACTACTATTAGTCATTATTTCA : 329
AB-i1-c16-F : TTTTTTTTT-TATTAAATAATATAATTTAATAATTGTCCTGTAATCTAATTATTCATTATAATATTATTTTCAATTTGAAATCTAATCTACTACTATTAGTCATTATTTCA : 329
AB-i1-c16-R : TTTTTTTTT-TATTAAATAATATAATTTAATAATTGTCCTGTAATCTAATTATTCATTATAATATTATTTTCAATTTGAAATCTAATCTACTACTATTAGTCATTATTTCA : 329
AB-i1-c17-F : TTTTTTTTT-TATTAAATAATATAATTTAATAATTGTCCTGTAATCTAATTATTCATTATAATATTATTTTCAATTTGAAATCTAATCTACTACTATTAGTCATTATTTCA : 329
AB-i1-c17-R : TTTTTTTTT-TATTAAATAATATAATTTAATAATTGTCCTGTAATCTAATTATTCATTATAATATTATTTTCAATTTGAAATCTAATCTACTACTATTAGTCATTATTTCA : 329
AB-i1-c18-F : TTTTTTTTT-TATTAAATAATATAATTTAATAATTGTCCTGTAATCTAATTATTCATTATAATATTATTTTCAATTTGAAATCTAATCTACTACTATTAGTCATTATTTCA : 328
AB-i1-c18-R : TTTTTTTTT-TATTAAATAATATAATTTAATAATTGTCCTGTAATCTAATTATTCATTATAATATTATTTTCAATTTGAAATCTAATCTACTACTATTAGTCATTATTTCA : 328
AB-i1-c19-F : TTTTTTTTT-TATTAAATAATATAATTTAATAATTGTCCTGTAATCTAATTATTCATTATAATATTATTTTCAATTTGAAATCTAATCTACTACTATTAGTCATTATTTCA : 329
AB-i1-c19-R : TTTTTTTTT-TATTAAATAATATAATTTAATAATTGTCCTGTAATCTAATTATTCATTATAATATTATTTTCAATTTGAAATCTAATCTACTACTATTAGTCATTATTTCA : 329
AB-i5-c1-F  : TTTTTTTTT-TATTAAATAATATAATTTAATAATTGTCCTGTAATCTAATTATTCATTATAATATTATTTTCAATTTGAAATCTAATCTACTACTATTAGTCATTATTTCA : 329
AB-i5-c1-R  : TTTTTTTTT-TATTAAATAATATAATTTAATAATTGTCCTGTAATCTAATTATTCATTATAATATTATTTTCAATTTGAAATCTAATCTACTACTATTAGTCATTATTTCA : 329
AB-i5-c2-F  : TTTTTTTTT-TATTAAATAATATAATTTAATAATTGTCCTGTAATCTAATTATTCATTATAATATTATTTTCAATTTGAAATCTAATCTACTACTATTAGTCATTATTTCA : 329
AB-i5-c2-R  : TTTTTTTTT-TATTAAATAATATAATTTAATAATTGTCCTGTAATCTAATTATTCATTATAATATTATTTTCAATTTGAAATCTAATCTACTACTATTAGTCATTATTTCA : 329
AB-i5-c6-F  : TTTTTTTTT-TATTAAATAATATAATTTAATAATTGTCCTGTAATCTAATTATTCATTATAATATTATTTTCAATTTGAAATCTAATCTACTACTATTAGTCATTATTTCA : 329
AB-i5-c6-R  : TTTTTTTTT-TATTAAATAATATAATTTAATAATTGTCCTGTAATCTAATTATTCATTATAATATTATTTTCAATTTGAAATCTAATCTACTACTATTAGTCATTATTTCA : 329
AB-i5-c7-F  : TTTTTTTTT-TATTAAATAATATAATTTAATAATTGTCCTGTAATCTAATTATTCATTATAATATTATTTTCAATTTGAAATCTAATCTACTACTATTAGTCATTATTTCA : 329
AB-i5-c7-R  : TTTTTTTTT-TATTAAATAATATAATTTAATAATTGTCCTGTAATCTAATTATTCATTATAATATTATTTTCAATTTGAAATCTAATCTACTACTATTAGTCATTATTTCA : 329
AB-i5-c11-F : TTTTTTTTT-TATTAAATAATATAATTTAATAATTGTCCTGTAATCTAATTATTCATTATAATATTATTTTCAATTTGAAATCTAATCTACTACTATTAGTCATTATTTCA : 329
AB-i5-c11-R : TTTTTTTTT-TATTAAATAATATAATTTAATAATTGTCCTGTAATCTAATTATTCATTATAATATTATTTTCAATTTGAAATCTAATCTACTACTATTAGTCATTATTTCA : 329
AB-i5-c12-F : TTTTTTTTT-TATTAAATAATATAATTTAATAATTGTCCTGTAATCTAATTATTCATTATAATATTATTTTCAATTTGAAATCTAATCTACTACTATTAGTCATTATTTCA : 329
AB-i5-c12-R : TTTTTTTTT-TATTAAATAATATAATTTAATAATTGTCCTGTAATCTAATTATTCATTATAATATTATTTTCAATTTGAAATCTAATCTACTACTATTAGTCATTATTTCA : 329
AB-i5-c13-F : TTTTTTTTT-TATTAAATAATATAATTTAATAATTGTCCTGTAATCTAATTATTCATTATAATATTATTTTCAATTTGAAATCTAATCTACTACTATTAGTCATTATTTCA : 328
AB-i5-c13-R : TTTTTTTTT-TATTAAATAATATAATTTAATAATTGTCCTGTAATCTAATTATTCATTATAATATTATTTTCAATTTGAAATCTAATCTACTACTATTAGTCATTATTTCA : 328
AB-i5-c14-F : TTTTTTTTT-TATTAAATAATATAATTTAATAATTGTCCTGTAATCTAATTATTCATTATAATATTATTTTCAATTTGAAATCTAATCTACTACTATTAGTCATTATTTCA : 329
AB-i5-c14-R : TTTTTTTTT-TATTAAATAATATAATTTAATAATTGTCCTGTAATCTAATTATTCATTATAATATTATTTTCAATTTGAAATCTAATCTACTACTATTAGTCATTATTTCA : 329
AB-i5-c15-F : TTTTTTTTT-TATTAAATAATATAATTTAATAATTGTCCTGTAATCTAATTATTCATTATAATATTATTTTCAATTTGAAATCTAATCTACTACTATTAGTCATTATTTCA : 329
AB-i5-c15-R : TTTTTTTTT-TATTAAATAATATAATTTAATAATTGTCCTGTAATCTAATTATTCATTATAATATTATTTTCAATTTGAAATCTAATCTACTACTATTAGTCATTATTTCA : 329
AB-i6-c4-F  : TTTTTTTTT-TATTAAATAATATAATTTAATAATTGTCCTGTAATCTAATTATTCATTATAATATTATTTTCAATTTGAAATCTAATCTACTACTATTAGTCATTATTTCA : 329
AB-i6-c4-R  : TTTTTTTTT-TATTAAATAATATAATTTAATAATTGTCCTGTAATCTAATTATTCATTATAATATTATTTTCAATTTGAAATCTAATCTACTACTATTAGTCATTATTTCA : 329
```

```
AB-i6-c7-F  : TTTTTTTT-TATTAAATAATATAATTTAATAATTGTCCTGTAATCTAATTATTCATTATAATATTATTTTCAATTTGAAATCTAATCTACTACTATTAGTCATTATTTCA : 329
AB-i6-c7-R  : TTTTTTTT-TATTAAATAATATAATTTAATAATTGTCCTGTAATCTAATTATTCATTATAATATTATTTTCAATTTGAAATCTAATCTACTACTATTAGTCATTATTTCA : 329
AB-i6-c8-F  : TTTTTTTTTTATTAAATAATATAATTTAATAATTGTCCTGTAATCTAATTATTCATTATAATATTATTTTCAATTTGAAATCTAATCTACTACTATTAGTCATTATTTCA : 330
AB-i6-c8-R  : TTTTTTTTTTATTAAATAATATAATTTAATAATTGTCCTGTAATCTAATTATTCATTATAATATTATTTTCAATTTGAAATCTAATCTACTACTATTAGTCATTATTTCA : 330
AB-i6-c9-F  : TTTTTTTT-TATTAAATAATATAATTTAATAATTGTCCTGTAATCTAATTATTCATTATAATATTATTTTCAATTTGAAATCTAATCTACTACTATTAGTCATTATTTCA : 329
AB-i6-c9-R  : TTTTTTTT-TATTAAATAATATAATTTAATAATTGTCCTGTAATCTAATTATTCATTATAATATTATTTTCAATTTGAAATCTAATCTACTACTATTAGTCATTATTTCA : 329
AB-i6-c11-F : TTTTTTTT-TATTAAATAATATAATTTAATAATTGTCCTGTAATCTAATTATTCATTATAATATTATTTTCAATTTGAAATCTAATCTACTACTATTAGTCATTATTTCA : 327
AB-i6-c11-R : TTTTTTTT-TATTAAATAATATAATTTAATAATTGTCCTGTAATCTAATTATTCATTATAATATTATTTTCAATTTGAAATCTAATCTACTACTATTAGTCATTATTTCA : 327
AB-i6-c12-F : TTTTTTTT-TATTAAATAATATAATTTAATAATTGTCCTGTAATCTAATTATTCATTATAATATTATTTTCAATTTGAAATCTAATCTACTACTATTAGTCATTATTTCA : 329
AB-i6-c12-R : TTTTTTTT-TATTAAATAATATAATTTAATAATTGTCCTGTAATCTAATTATTCATTATAATATTATTTTCAATTTGAAATCTAATCTACTACTATTAGTCATTATTTCA : 329
AB-i6-c17-F : TTTTTTTT-TATTAAATAATATAATTTAATAATTGTCCTGTAATCTAATTATTCATTATAATATTATTTTCAATTTGAAATCTAATCTACTACTATTAGTCATTATTTCA : 329
AB-i6-c17-R : TTTTTTTT-TATTAAATAATATAATTTAATAATTGTCCTGTAATCTAATTATTCATTATAATATTATTTTCAATTTGAAATCTAATCTACTACTATTAGTCATTATTTCA : 329
AB-i7-c11-F : TTTTTTTT-TATTAAATAATATAATTTAATAATTGTCCTGTAATCTAATTATTCATTATAATATTATTTTCAATTTGAAATCTAATCTACTACTATTAGTCATTATTTCA : 329
AB-i7-c11-R : TTTTTTTT-TATTAAATAATATAATTTAATAATTGTCCTGTAATCTAATTATTCATTATAATATTATTTTCAATTTGAAATCTAATCTACTACTATTAGTCATTATTTCA : 329
AB-i7-c12-F : TTTTTTTT-TATTAAATAATATAATTTAATAATTGTCCTGTAATCTAATTATTCATTATAATATTATTTTCAATTTGAAATCTAATCTACTACTATTAGTCATTATTTCA : 329
AB-i7-c12-R : TTTTTTTT-TATTAAATAATATAATTTAATAATTGTCCTGTAATCTAATTATTCATTATAATATTATTTTCAATTTGAAATCTAATCTACTACTATTAGTCATTATTTCA : 329
AB-i7-c13-F : TTTTTTTT-TATTAAATAATATAATTTAATAATTGTCCTGTAATCTAATTATTCATTATAATATTATTTTCAATTTGAAATCTAATCTACTACTATTAGTCATTATTTCA : 329
AB-i7-c13-R : TTTTTTTT-TATTAAATAATATAATTTAATAATTGTCCTGTAATCTAATTATTCATTATAATATTATTTTCAATTTGAAATCTAATCTACTACTATTAGTCATTATTTCA : 329
AB-i7-c14-F : TTTTTTTT-TATTAAATAATATAATTTAATAATTGTCCTGTAATCTAATTATTCATTATAATATTATTTTCAATTTGAAATCTAATCTACTACTATTAGTCATTATTTCA : 329
AB-i7-c14-R : TTTTTTTT-TATTAAATAATATAATTTAATAATTGTCCTGTAATCTAATTATTCATTATAATATTATTTTCAATTTGAAATCTAATCTACTACTATTAGTCATTATTTCA : 329
AB-i7-c16-F : TTTTTTTT-TATTAAATAATATAATTTAATAATTGTCCTGTAATCTAATTATTCATTATAATATTATTTTCAATTTGAAATCTAATCTACTACTATTAGTCATTATTTCA : 329
AB-i7-c16-R : TTTTTTTT-TATTAAATAATATAATTTAATAATTGTCCTGTAATCTAATTATTCATTATAATATTATTTTCAATTTGAAATCTAATCTACTACTATTAGTCATTATTTCA : 329
AB-i7-c18-F : TTTTTTT--TATTAAATAATATAATTTAATAATTGTCCTGTAATCTAATTATTCATTATAATATTATTTTCAATTTGAAATCTAATCTACTACTATTAGTCATTATTTCA : 328
AB-i7-c18-R : TTTTTTT--TATTAAATAATATAATTTAATAATTGTCCTGTAATCTAATTATTCATTATAATATTATTTTCAATTTGAAATCTAATCTACTACTATTAGTCATTATTTCA : 328
AB-i7-c19-F : TTTTTTTT-TATTAAATAATATAATTTAATAATTGTCCTGTAATCTAATTATTCATTATAATATTATTTTCAATTTGAAATCTAATCTACTACTATTAGTCATTATTTCA : 328
AB-i7-c19-R : TTTTTTTT-TATTAAATAATATAATTTAATAATTGTCCTGTAATCTAATTATTCATTATAATATTATTTTCAATTTGAAATCTAATCTACTACTATTAGTCATTATTTCA : 328
AB-i7-c20-F : TTTTTTTT-TATTAAATAATATAATTTAATAATTGTCCTGTAATCTAATTATTCATTATAATATTATTTTCAATTTGAAATCTAATCTACTACTATTAGTCATTATTTCA : 329
AB-i7-c20-R : TTTTTTTT-TATTAAATAATATAATTTAATAATTGTCCTGTAATCTAATTATTCATTATAATATTATTTTCAATTTGAAATCTAATCTACTACTATTAGTCATTATTTCA : 329
              TTTTTTTt TATTAAATAATATAATTTAATAATTGTCCTGTAATCTAATTATTCATTATAATATTATTTTCAATTTGAAATCTAATCTACTACTATTAGTCATTATTTCA

                   340         *       360         *       380         *       400         *       420         *       440
AB          : AAGATTGAAATAAAGATTTGTATTTGAAATAGTATTTTACATATTTATTATATTTAAATTTAAATATATATTTA----------AAATAGAAGTAGAAGGTATTCTAGTT : 429
AB-i1-c4-F  : AAGATTGAAATAAAGATTTGTATTTGAAATAGTATTTTACATATTTATTATATTTAAATTTAAATATATATTTATATATATTTAAAATAGAAGTAGAAGGTATTCTAGTT : 439
AB-i1-c4-R  : AAGATTGAAATAAAGATTTGTATTTGAAATAGTATTTTACATATTTATTATATTTAAATTTAAATATATATTTATATATATTTAAAATAGAAGTAGAAGGTATTCTAGTT : 439
AB-i1-c11-F : AAGATTGAAATAAAGATTTGTATTTGAAATAGTATTTTACATATTTATTATATTTAAATTTAAATATATATTTATATATATTTAAAATAGAAGTAGAAGGTATTCTAGTT : 439
AB-i1-c11-R : AAGATTGAAATAAAGATTTGTATTTGAAATAGTATTTTACATATTTATTATATTTAAATTTAAATATATATTTATATATATTTAAAATAGAAGTAGAAGGTATTCTAGTT : 439
AB-i1-c14-F : AAGATTGAAATAAAGATTTGTATTTGAAATAGTATTTTACATATTTATTATATTTAAATTTAAATATATATTTATATATATTTAAAATAGAAGTAGAAGGTATTCTAGTT : 439
AB-i1-c14-R : AAGATTGAAATAAAGATTTGTATTTGAAATAGTATTTTACATATTTATTATATTTAAATTTAAATATATATTTATATATATTTAAAATAGAAGTAGAAGGTATTCTAGTT : 439
AB-i1-c15-F : AAGATTGAAATAAAGATTTGTATTTGAAATAGTATTTTACATATTTATTATATTTAAATTTAAATATATATTTATATATATTTAAAATAGAAGTAGAAGGTATTCTAGTT : 439
AB-i1-c15-R : AAGATTGAAATAAAGATTTGTATTTGAAATAGTATTTTACATATTTATTATATTTAAATTTAAATATATATTTATATATATTTAAAATAGAAGTAGAAGGTATTCTAGTT : 439
AB-i1-c16-F : AAGATTGAAATAAAGATTTGTATTTGAAATAGTATTTTACATATTTATTATATTTAAATTTAAATATATATTTATATATATTTAAAATAGAAGTAGAAGGTATTCTAGTT : 439
AB-i1-c16-R : AAGATTGAAATAAAGATTTGTATTTGAAATAGTATTTTACATATTTATTATATTTAAATTTAAATATATATTTATATATATTTAAAATAGAAGTAGAAGGTATTCTAGTT : 439
AB-i1-c17-F : AAGATTGAAATAAAGATTTGTATTTGAAATAGTATTTTACATATTTATTATATTTAAATTTAAATATATATTTATATATATTTAAAATAGAAGTAGAAGGTATTCTAGTT : 439
AB-i1-c17-R : AAGATTGAAATAAAGATTTGTATTTGAAATAGTATTTTACATATTTATTATATTTAAATTTAAATATATATTTATATATATTTAAAATAGAAGTAGAAGGTATTCTAGTT : 439
AB-i1-c18-F : AAGATTGAAATAAAGATTTGTATTTGAAATAGTATTTTACATATTTATTATATTTAAATTTAAATATATATTTATATATATTTAAAATAGAAGTAGAAGGTATTCTAGTT : 438
AB-i1-c18-R : AAGATTGAAATAAAGATTTGTATTTGAAATAGTATTTTACATATTTATTATATTTAAATTTAAATATATATTTATATATATTTAAAATAGAAGTAGAAGGTATTCTAGTT : 438
```

```
AB-i1-c19-F : AAGATTGAAATAAAGATTTGTATTTGAAATAGTATTTTACATATTTATTATATTTAAATTTAAATATATATATTTATATATATTTAAAATAGAAGTAGAAGGTATTCTAGTT : 439
AB-i1-c19-R : AAGATTGAAATAAAGATTTGTATTTGAAATAGTATTTTACATATTTATTATATTTAAATTTAAATATATATATTTATATATATTTAAAATAGAAGTAGAAGGTATTCTAGTT : 439
AB-i5-c1-F  : AAGATTGAAATAAAGATTTGTATTTGAAATAGTATTTTACATATTTATTATATTTAAATTTAAATATATATATTTA----------AAATAGAAGTAGAAGGTATTCTAGTT : 429
AB-i5-c1-R  : AAGATTGAAATAAAGATTTGTATTTGAAATAGTATTTTACATATTTATTATATTTAAATTTAAATATATATATTTA----------AAATAGAAGTAGAAGGTATTCTAGTT : 429
AB-i5-c2-F  : AAGATTGAAATAAAGATTTGTATTTGAAATAGTATTTTACATATTTATTATATTTAAATTTAAATATATATATTTA----------AAATAGAAGTAGAAGGTATTCTAGTT : 429
AB-i5-c2-R  : AAGATTGAAATAAAGATTTGTATTTGAAATAGTATTTTACATATTTATTATATTTAAATTTAAATATATATATTTA----------AAATAGAAGTAGAAGGTATTCTAGTT : 429
AB-i5-c6-F  : AAGATTGAAATAAAGATTTGTATTTGAAATAGTATTTTACATATTTATTATATTTAAATTTAAATATATATATTTA----------AAATAGAAGTAGAAGGTATTCTAGTT : 429
AB-i5-c6-R  : AAGATTGAAATAAAGATTTGTATTTGAAATAGTATTTTACATATTTATTATATTTAAATTTAAATATATATATTTA----------AAATAGAAGTAGAAGGTATTCTAGTT : 429
AB-i5-c7-F  : AAGATTGAAATAAAGATTTGTATTTGAAATAGTATTTTACATATTTATTATATTTAAATTTAAATATATATATTT--A--------AAATAGAAGTAGAAGGTATTCTAGTT : 429
AB-i5-c7-R  : AAGATTGAAATAAAGATTTGTATTTGAAATAGTATTTTACATATTTATTATATTTAAATTTAAATATATATATTT----------AAAATAGAAGTAGAAGGTATTCTAGTT : 429
AB-i5-c11-F : AAGATTGAAATAAAGATTTGTATTTGAAATAGTATTTTACATATTTATTATATTTAAATTTAAATATATATATTTA----------AAATAGAAGTAGAAGGTATTCTAGTT : 429
AB-i5-c11-R : AAGATTGAAATAAAGATTTGTATTTGAAATAGTATTTTACATATTTATTATATTTAAATTTAAATATATATATTTA----------AAATAGAAGTAGAAGGTATTCTAGTT : 429
AB-i5-c12-F : AAGATTGAAATAAAGATTTGTATTTGAAATAGTATTTTACATATTTATTATATTTAAATTTAAATATATATATTTA----------AAATAGAAGTAGAAGGTATTCTAGTT : 429
AB-i5-c12-R : AAGATTGAAATAAAGATTTGTATTTGAAATAGTATTTTACATATTTATTATATTTAAATTTAAATATATATATTTA----------AAATAGAAGTAGAAGGTATTCTAGTT : 429
AB-i5-c13-F : AAGATTGAAATAAAGATTTGTATTTGAAATAGTATTTTACATATTTATTATATTTAAATTTAAATATATATATTTA----------AAATAGAAGTAGAAGGTATTCTAGTT : 428
AB-i5-c13-R : AAGATTGAAATAAAGATTTGTATTTGAAATAGTATTTTACATATTTATTATATTTAAATTTAAATATATATATTTA----------AAATAGAAGTAGAAGGTATTCTAGTT : 428
AB-i5-c14-F : AAGATTGAAATAAAGATTTGTATTTGAAATAGTATTTTACATATTTATTATATTTAAATTTAAATATATATATTTA----------AAATAGAAGTAGAAGGTATTCTAGTT : 429
AB-i5-c14-R : AAGATTGAAATAAAGATTTGTATTTGAAATAGTATTTTACATATTTATTATATTTAAATTTAAATATATATATTTA----------AAATAGAAGTAGAAGGTATTCTAGTT : 429
AB-i5-c15-F : AAGATTGAAATAAAGATTTGTATTTGAAATAGTATTTTACATATTTATTATATTTAAATTTAAATATATATATTTA----------AAATAGAAGTAGAAGGTATTCTAGTT : 429
AB-i5-c15-R : AAGATTGAAATAAAGATTTGTATTTGAAATAGTATTTTACATATTTATTATATTTAAATTTAAATATATATATTTA----------AAATAGAAGTAGAAGGTATTCTAGTT : 429
AB-i6-c4-F  : AAGATTGAAATAAAGATTTGTATTTGAAATAGTATTTTACATATTTATTATATTTAAATTTAAATATATATATTTA----------AAATAGAAGTAGAAGGTATTCTAGTT : 429
AB-i6-c4-R  : AAGATTGAAATAAAGATTTGTATTTGAAATAGTATTTTACATATTTATTATATTTAAATTTAAATATATATATTTA----------AAATAGAAGTAGAAGGTATTCTAGTT : 429
AB-i6-c7-F  : AAGATTGAAATAAAGATTTGTATTTGAAATAGTATTTTACATATTTATTATATTTAAATTTAAATATATATATTTA----------AAATAGAAGTAGAAGGTATTCTAGTT : 429
AB-i6-c7-R  : AAGATTGAAATAAAGATTTGTATTTGAAATAGTATTTTACATATTTATTATATTTAAATTTAAATATATATATTTA----------AAATAGAAGTAGAAGGTATTCTAGTT : 429
AB-i6-c8-F  : AAGATTGAAATAAAGATTTGTATTTGAAATAGTATTTTACATATTTATTATATTTAAATTTAAATATATATATTTA----------AAATAGAAGTAGAAGGTATTCTAGTT : 430
AB-i6-c8-R  : AAGATTGAAATAAAGATTTGTATTTGAAATAGTATTTTACATATTTATTATATTTAAATTTAAATATATATATTTA----------AAATAGAAGTAGAAGGTATTCTAGTT : 430
AB-i6-c9-F  : AAGATTGAAATAAAGATTTGTATTTGAAATAGTATTTTACATATTTATTATATTTAAATTTAAATATATATATTTA----------AAATAGAAGTAGAAGGTATTCTAGTT : 429
AB-i6-c9-R  : AAGATTGAAATAAAGATTTGTATTTGAAATAGTATTTTACATATTTATTATATTTAAATTTAAATATATATATTTA----------AAATAGAAGTAGAAGGTATTCTAGTT : 429
AB-i6-c11-F : AAGATTGAAATAAAGATTTGTATTTGAAATAGTATTTTACATATTTATTATATTTAAATTTAAATATATATATTTA----------AAATAGAAGTAGAAGGTATTCTAGTT : 427
AB-i6-c11-R : AAGATTGAAATAAAGATTTGTATTTGAAATAGTATTTTACATATTTATTATATTTAAATTTAAATATATATATTTA----------AAATAGAAGTAGAAGGTATTCTAGTT : 427
AB-i6-c12-F : AAGATTGAAATAAAGATTTGTATTTGAAATAGTATTTTACATATTTATTATATTTAAATTTAAATATATATATTTA----------AAATAGAAGTAGAAGGTATTCTAGTT : 429
AB-i6-c12-R : AAGATTGAAATAAAGATTTGTATTTGAAATAGTATTTTACATATTTATTATATTTAAATTTAAATATATATATTTA----------AAATAGAAGTAGAAGGTATTCTAGTT : 429
AB-i6-c17-F : AAGATTGAAATAAAGATTTGTATTTGAAATAGTATTTTACATATTTATTATATTTAAATTTAAATATATATATTTA----------AAATAGAAGTAGAAGGTATTCTAGTT : 429
AB-i6-c17-R : AAGATTGAAATAAAGATTTGTATTTGAAATAGTATTTTACATATTTATTATATTTAAATTTAAATATATATATTTA----------AAATAGAAGTAGAAGGTATTCTAGTT : 429
AB-i7-c11-F : AAGATTGAAATAAAGATTTGTATTTGAAATAGTATTTTACATATTTATTATATTTAAATTTAAATATATATATTTATATATATTTAAAATAGAAGTAGAAGGTATTCTAGTT : 439
AB-i7-c11-R : AAGATTGAAATAAAGATTTGTATTTGAAATAGTATTTTACATATTTATTATATTTAAATTTAAATATATATATTTATATATATTTAAAATAGAAGTAGAAGGTATTCTAGTT : 439
AB-i7-c12-F : AAGATTGAAATAAAGATTTGTATTTGAAATAGTATTTTACATATTTATTATATTTAAATTTAAATATATATATTTATATATATTTAAAATAGAAGTAGAAGGTATTCTAGTT : 439
AB-i7-c12-R : AAGATTGAAATAAAGATTTGTATTTGAAATAGTATTTTACATATTTATTATATTTAAATTTAAATATATATATTTATATATATTTAAAATAGAAGTAGAAGGTATTCTAGTT : 439
AB-i7-c13-F : AAGATTGAAATAAAGATTTGTATTTGAAATAGTATTTTACATATTTATTATATTTAAATTTAAATATATATATTTA----------AAATAGAAGTAGAAGGTATTCTAGTT : 429
AB-i7-c13-R : AAGATTGAAATAAAGATTTGTATTTGAAATAGTATTTTACATATTTATTATATTTAAATTTAAATATATATATTTA----------AAATAGAAGTAGAAGGTATTCTAGTT : 429
AB-i7-c14-F : AAGATTGAAATAAAGATTTGTATTTGAAATAGTATTTTACATATTTATTATATTTAAATTTAAATATATATATTTATATATATTTAAAATAGAAGTAGAAGGTATTCTAGTT : 439
AB-i7-c14-R : AAGATTGAAATAAAGATTTGTATTTGAAATAGTATTTTACATATTTATTATATTTAAATTTAAATATATATATTTATATATATTTAAAATAGAAGTAGAAGGTATTCTAGTT : 439
AB-i7-c16-F : AAGATTGAAATAAAGATTTGTATTTGAAATAGTATTTTACATATTTATTATATTTAAATTTAAATATATATATTTATATATATTTAAAATAGAAGTAGAAGGTATTCTAGTT : 439
AB-i7-c16-R : AAGATTGAAATAAAGATTTGTATTTGAAATAGTATTTTACATATTTATTATATTTAAATTTAAATATATATATTTATATATATTTAAAATAGAAGTAGAAGGTATTCTAGTT : 439
AB-i7-c18-F : AAGATTGAAATAAAGATTTGTATTTGAAATAGTATTTTACATATTTATTATATTTAAATTTAAATATATATATTTA----------AAATAGAAGTAGAAGGTATTCTAGTT : 428
AB-i7-c18-R : AAGATTGAAATAAAGATTTGTATTTGAAATAGTATTTTACATATTTATTATATTTAAATTTAAATATATATATTTA----------AAATAGAAGTAGAAGGTATTCTAGTT : 428
```

```
AB-i7-c19-F : AAGATTGAAATAAAGATTTGTATTTGAAATAGTATTTTACATATTTATTATATTTAAATTTAAATATATATATTTATATATATTTAAAATAGAAGTAGAAGGTATTCTAGTT : 438
AB-i7-c19-R : AAGATTGAAATAAAGATTTGTATTTGAAATAGTATTTTACATATTTATTATATTTAAATTTAAATATATATATTTATATATATTTAAAATAGAAGTAGAAGGTATTCTAGTT : 438
AB-i7-c20-F : AAGATTGAAATAAAGATTTGTATTTGAAATAGTATTTTACATATTTATTATATTTAAATTTAAATATATATATTTATATATATTTAAAATAGAAGTAGAAGGTATTCTAGTT : 439
AB-i7-c20-R : AAGATTGAAATAAAGATTTGTATTTGAAATAGTATTTTACATATTTATTATATTTAAATTTAAATATATATATTTATATATATTTAAAATAGAAGTAGAAGGTATTCTAGTT : 439
              AAGATTGAAATAAAGATTTGTATTTGAAATAGTATTTTACATATTTATTATATTTAAATTTAAATATATATATTTa          AAATAGAAGTAGAAGGTATTCTAGTT

                    *       460         *       480         *       500         *       520         *       540         *
AB          : CTATAATTCTTATTATATTAATTATAATTATTAATATATAATAATATATATTATAGAAAAAACTTATTAATAAATTTATTAAATTAAATAATAGATATAATAATAGATAT : 539
AB-i1-c4-F  : CTATAATTCTTATTATATTAATTATAATTATTAATATATAATAATATATATTATAGAAAAAACTTATTAATAAATTTATTAAATTAAATAATAGATATAATAATAGATAT : 549
AB-i1-c4-R  : CTATAATTCTTATTATATTAATTATAATTATTAATATATAATAATATATATTATAGAAAAAACTTATTAATAAATTTATTAAATTAAATAATAGATATAATAATAGATAT : 549
AB-i1-c11-F : CTATAATTCTTATTATATTAATTATAATTATTAATATATAATAATATATATTATAGAAAAAACTTATTAATAAATTTATTAAATTAAATAATAGATATAATAATAGATAT : 549
AB-i1-c11-R : CTATAATTCTTATTATATTAATTATAATTATTAATATATAATAATATATATTATAGAAAAAACTTATTAATAAATTTATTAAATTAAATAATAGATATAATAATAGATAT : 549
AB-i1-c14-F : CTATAATTCTTATTATATTAATTATAATTATTAATATATAATAATATATATTATAGAAAAAACTTATTAATAAATTTATTAAATTAAATAATAGATATAATAATAGATAT : 549
AB-i1-c14-R : CTATAATTCTTATTATATTAATTATAATTATTAATATATAATAATATATATTATAGAAAAAACTTATTAATAAATTTATTAAATTAAATAATAGATATAATAATAGATAT : 549
AB-i1-c15-F : CTATAATTCTTATTATATTAATTATAATTATTAATATATAATAATATATATTATAGAAAAAACTTATTAATAAATTTATTAAATTAAATAATAGATATAATAATAGATAT : 549
AB-i1-c15-R : CTATAATTCTTATTATATTAATTATAATTATTAATATATAATAATATATATTATAGAAAAAACTTATTAATAAATTTATTAAATTAAATAATAGATATAATAATAGATAT : 549
AB-i1-c16-F : CTATAATTCTTATTATATTAATTATAATTATTAATATATAATAATATATATTATAGAAAAAACTTATTAATAAATTTATTAAATTAAATAATAGATATAATAATAGATAT : 549
AB-i1-c16-R : CTATAATTCTTATTATATTAATTATAATTATTAATATATAATAATATATATTATAGAAAAAACTTATTAATAAATTTATTAAATTAAATAATAGATATAATAATAGATAT : 549
AB-i1-c17-F : CTATAATTCTTATTATATTAATTATAATTATTAATATATAATAATATATATTATAGAAAAAACTTATTAATAAATTTATTAAATTAAATAATAGATATAATAATAGATAT : 549
AB-i1-c17-R : CTATAATTCTTATTATATTAATTATAATTATTAATATATAATAATATATATTATAGAAAAAACTTATTAATAAATTTATTAAATTAAATAATAGATATAATAATAGATAT : 549
AB-i1-c18-F : CTATAATTCTTATTATATTAATTATAATTATTAATATATAATAATATATATTATAGAAAAAACTTATTAATAAATTTATTAAATTAAATAATAGATATAATAATAGATAT : 548
AB-i1-c18-R : CTATAATTCTTATTATATTAATTATAATTATTAATATATAATAATATATATTATAGAAAAAACTTATTAATAAATTTATTAAATTAAATAATAGATATAATAATAGATAT : 548
AB-i1-c19-F : CTATAATTCTTATTATATTAATTATAATTATTAATATATAATAATATATATTATAGAAAAAACTTATTAATAAATTTATTAAATTAAATAATAGATATAATAATAGATAT : 549
AB-i1-c19-R : CTATAATTCTTATTATATTAATTATAATTATTAATATATAATAATATATATTATAGAAAAAACTTATTAATAAATTTATTAAATTAAATAATAGATATAATAATAGATAT : 549
AB-i5-c1-F  : CTATAATTCTTATTATATTAATTATAATTATTAATATATAATAATATATATTATAGAAAAAACTTATTAATAAATTTATTAAATTAAATAATAGATATAATAATAGATAT : 539
AB-i5-c1-R  : CTATAATTCTTATTATATTAATTATAATTATTAATATATAATAATATATATTATAGAAAAAACTTATTAATAAATTTATTAAATTAAATAATAGATATAATAATAGATAT : 539
AB-i5-c2-F  : CTATAATTCTTATTATATTAATTATAATTATTAATATATAATAATATATATTATAGAAAAAACTTATTAATAAATTTATTAAATTAAATAATAGATATAATAATAGATAT : 539
AB-i5-c2-R  : CTATAATTCTTATTATATTAATTATAATTATTAATATATAATAATATATATTATAGAAAAAACTTATTAATAAATTTATTAAATTAAATAATAGATATAATAATAGATAT : 539
AB-i5-c6-F  : CTATAATTCTTATTATATTAATTATAATTATTAATATATAATAATATATATTATAGAAAAAACTTATTAATAAATTTATTAAATTAAATAATAGATATAATAATAGATAT : 539
AB-i5-c6-R  : CTATAATTCTTATTATATTAATTATAATTATTAATATATAATAATATATATTATAGAAAAAACTTATTAATAAATTTATTAAATTAAATAATAGATATAATAATAGATAT : 539
AB-i5-c7-F  : CTATAATTCTTATTATATTAATTATAATTATTAATATATAATAATATATATTATAGAAAAAACTTATTAATAAATTTATTAAATTAAATAATAGATATAATAATAGATAT : 539
AB-i5-c7-R  : CTATAATTCTTATTATATTAATTATAATTATTAATATATAATAATATATATTATAGAAAAAACTTATTAATAAATTTATTAAATTAAATAATAGATATAATAATAGATAT : 539
AB-i5-c11-F : CTATAATTCTTATTATATTAATTATAATTATTAATATATAATAATATATATTATAGAAAAAACTTATTAATAAATTTATTAAATTAAATAATAGATATAATAATAGATAT : 539
AB-i5-c11-R : CTATAATTCTTATTATATTAATTATAATTATTAATATATAATAATATATATTATAGAAAAAACTTATTAATAAATTTATTAAATTAAATAATAGATATAATAATAGATAT : 539
AB-i5-c12-F : CTATAATTCTTATTATATTAATTATAATTATTAATATATAATAATATATATTATAGAAAAAACTTATTAATAAATTTATTAAATTAAATAATAGATATAATAATAGATAT : 539
AB-i5-c12-R : CTATAATTCTTATTATATTAATTATAATTATTAATATATAATAATATATATTATAGAAAAAACTTATTAATAAATTTATTAAATTAAATAATAGATATAATAATAGATAT : 539
AB-i5-c13-F : CTATAATTCTTATTATATTAATTATAATTATTAATATATAATAATATATATTATAGAAAAAACTTATTAATAAATTTATTAAATTAAATAATAGATATAATAATAGATAT : 538
AB-i5-c13-R : CTATAATTCTTATTATATTAATTATAATTATTAATATATAATAATATATATTATAGAAAAAACTTATTAATAAATTTATTAAATTAAATAATAGATATAATAATAGATAT : 538
AB-i5-c14-F : CTATAATTCTTATTATATTAATTATAATTATTAATATATAATAATATATATTATAGAAAAAACTTATTAATAAATTTATTAAATTAAATAATAGATATAATAATAGATAT : 539
AB-i5-c14-R : CTATAATTCTTATTATATTAATTATAATTATTAATATATAATAATATATATTATAGAAAAAACTTATTAATAAATTTATTAAATTAAATAATAGATATAATAATAGATAT : 539
AB-i5-c15-F : CTATAATTCTTATTATATTAATTATAATTATTAATATATAATAATATATATTATAGAAAAAACTTATTAATAAATTTATTAAATTAAATAATAGATATAATAATAGATAT : 539
AB-i5-c15-R : CTATAATTCTTATTATATTAATTATAATTATTAATATATAATAATATATATTATAGAAAAAACTTATTAATAAATTTATTAAATTAAATAATAGATATAATAATAGATAT : 539
AB-i6-c4-F  : CTATAATTCTTATTATATTAATTATAATTATTAATATATAATAATATATATTATAGAAAAAACTTATTAATAAATTTATTAAATTAAATAATAGATATAATAATAGATAT : 539
AB-i6-c4-R  : CTATAATTCTTATTATATTAATTATAATTATTAATATATAATAATATATATTATAGAAAAAACTTATTAATAAATTTATTAAATTAAATAATAGATATAATAATAGATAT : 539
AB-i6-c7-F  : CTATAATTCTTATTATATTAATTATAATTATTAATATATAATAATATATATTATAGAAAAAACTTATTAATAAATTTATTAAATTAAATAATAGATATAATAATAGATAT : 539
AB-i6-c7-R  : CTATAATTCTTATTATATTAATTATAATTATTAATATATAATAATATATATTATAGAAAAAACTTATTAATAAATTTATTAAATTAAATAATAGATATAATAATAGATAT : 539
```

```
AB-i6-c8-F  : CTATAATTCTTATTATATTAATTATAATTATTAATATATAATAATATATATTATAGAAAAAACTTATTAATAAATTTATTAAATTAAATAATAGATATAATAATAGATAT : 540
AB-i6-c8-R  : CTATAATTCTTATTATATTAATTATAATTATTAATATATAATAATATATATTATAGAAAAAACTTATTAATAAATTTATTAAATTAAATAATAGATATAATAATAGATAT : 540
AB-i6-c9-F  : CTATAATTCTTATTATATTAATTATAATTATTAATATATAATAATATATATTATAGAAAAAACTTATTAATAAATTTATTAAATTAAATAATAGATATAATAATAGATAT : 539
AB-i6-c9-R  : CTATAATTCTTATTATATTAATTATAATTATTAATATATAATAATATATATTATAGAAAAAACTTATTAATAAATTTATTAAATTAAATAATAGATATAATAATAGATAT : 539
AB-i6-c11-F : CTATAATTCTTATTATATTAATTATAATTATTAATATATAATAATATATATTATAGAAAAAACTTATTAATAAATTTATTAAATTAAATAATAGATATAATAATAGATAT : 537
AB-i6-c11-R : CTATAATTCTTATTATATTAATTATAATTATTAATATATAATAATATATATTATAGAAAAAACTTATTAATAAATTTATTAAATTAAATAATAGATATAATAATAGATAT : 537
AB-i6-c12-F : CTATAATTCTTATTATATTAATTATAATTATTAATATATAATAATATATATTATAGAAAAAACTTATTAATAAATTTATTAAATTAAATAATAGATATAATAATAGATAT : 539
AB-i6-c12-R : CTATAATTCTTATTATATTAATTATAATTATTAATATATAATAATATATATTATAGAAAAAACTTATTAATAAATTTATTAAATTAAATAATAGATATAATAATAGATAT : 539
AB-i6-c17-F : CTATAATTCTTATTATATTAATTATAATTATTAATATATAATAATATATATTATAGAAAAAACTTATTAATAAATTTATTAAATTAAATAATAGATATAATAATAGATAT : 539
AB-i6-c17-R : CTATAATTCTTATTATATTAATTATAATTATTAATATATAATAATATATATTATAGAAAAAACTTATTAATAAATTTATTAAATTAAATAATAGATATAATAATAGATAT : 539
AB-i7-c11-F : CTATAATTCTTATTATATTAATTATAATTATTAATATATAATAATATATATTATAGAAAAAACTTATTAATAAATTTATTAAATTAAATAATAGATATAATAATAGATAT : 549
AB-i7-c11-R : CTATAATTCTTATTATATTAATTATAATTATTAATATATAATAATATATATTATAGAAAAAACTTATTAATAAATTTATTAAATTAAATAATAGATATAATAATAGATAT : 549
AB-i7-c12-F : CTATAATTCTTATTATATTAATTATAATTATTAATATATAATAATATATATTATAGAAAAAACTTATTAATAAATTTATTAAATTAAATAATAGATATAATAATAGATAT : 549
AB-i7-c12-R : CTATAATTCTTATTATATTAATTATAATTATTAATATATAATAATATATATTATAGAAAAAACTTATTAATAAATTTATTAAATTAAATAATAGATATAATAATAGATAT : 549
AB-i7-c13-F : CTATAATTCTTATTATATTAATTATAATTATTAATATATAATAATATATATTATAGAAAAAACTTATTAATAAATTTATTAAATTAAATAATAGATATAATAATAGATAT : 539
AB-i7-c13-R : CTATAATTCTTATTATATTAATTATAATTATTAATATATAATAATATATATTATAGAAAAAACTTATTAATAAATTTATTAAATTAAATAATAGATATAATAATAGATAT : 539
AB-i7-c14-F : CTATAATTCTTATTATATTAATTATAATTATTAATATATAATAATATATATTATAGAAAAAACTTATTAATAAATTTATTAAATTAAATAATAGATATAATAATAGATAT : 549
AB-i7-c14-R : CTATAATTCTTATTATATTAATTATAATTATTAATATATAATAATATATATTATAGAAAAAACTTATTAATAAATTTATTAAATTAAATAATAGATATAATAATAGATAT : 549
AB-i7-c16-F : CTATAATTCTTATTATATTAATTATAATTATTAATATATAATAATATATATTATAGAAAAAACTTATTAATAAATTTATTAAATTAAATAATAGATATAATAATAGATAT : 549
AB-i7-c16-R : CTATAATTCTTATTATATTAATTATAATTATTAATATATAATAATATATATTATAGAAAAAACTTATTAATAAATTTATTAAATTAAATAATAGATATAATAATAGATAT : 549
AB-i7-c18-F : CTATAATTCTTATTATATTAATTATAATTATTAATATATAATAATATATATTATAGAAAAAACTTATTAATAAATTTATTAAATTAAATAATAGATATAATAATAGATAT : 538
AB-i7-c18-R : CTATAATTCTTATTATATTAATTATAATTATTAATATATAATAATATATATTATAGAAAAAACTTATTAATAAATTTATTAAATTAAATAATAGATATAATAATAGATAT : 538
AB-i7-c19-F : CTATAATTCTTATTATATTAATTATAATTATTAATATATAATAATATATATTATAGAAAAAACTTATTAATAAATTTATTAAATTAAATAATAGATATAATAATAGATAT : 548
AB-i7-c19-R : CTATAATTCTTATTATATTAATTATAATTATTAATATATAATAATATATATTATAGAAAAAACTTATTAATAAATTTATTAAATTAAATAATAGATATAATAATAGATAT : 548
AB-i7-c20-F : CTATAATTCTTATTATATTAATTATAATTATTAATATATAATAATATATATTATAGAAAAAACTTATTAATAAATTTATTAAATTAAATAATAGATATAATAATAGATAT : 549
AB-i7-c20-R : CTATAATTCTTATTATATTAATTATAATTATTAATATATAATAATATATATTATAGAAAAAACTTATTAATAAATTTATTAAATTAAATAATAGATATAATAATAGATAT : 549
              CTATAATTCTTATTATATTAATTATAATTATTAATATATAATAATATATATTATAGAAAAAACTTATTAATAAATTTATTAAATTAAATAATAGATATAATAATAGATAT

                   560         *       580         *       600         *       620         *       640         *       660
AB          : ATTGAATAGAAGAGATATAATAATAATTATGTGTTAGATGTAAATATTTTATTTAATATATATATATTGTTATATATTATTAATATTTAAATTTATTTATCTATTATATTGA : 649
AB-i1-c4-F  : ATTGAATAGAAGAGATATAATAATAATTATGTGTTAGATGTAAATATTTTATTTAATATATATATATTGTTATATATTATTAATATTTAAATTTATTTATCTATTATATTGA : 659
AB-i1-c4-R  : ATTGAATAGAAGAGATATAATAATAATTATGTGTTAGATGTAAATATTTTATTTAATATATATATATTGTTATATATTATTAATATTTAAATTTATTTATCTATTATATTGA : 659
AB-i1-c11-F : ATTGAATAGAAGAGATATAATAATAATTATGTGTTAGATGTAAATATTTTATTTAATATATATATATTGTTATATATTATTAATATTTAAATTTATTTATCTATTATATTGA : 659
AB-i1-c11-R : ATTGAATAGAAGAGATATAATAATAATTATGTGTTAGATGTAAATATTTTATTTAATATATATATATTGTTATATATTATTAATATTTAAATTTATTTATCTATTATATTGA : 659
AB-i1-c14-F : ATTGAATAGAAGAGATATAATAATAATTATGTGTTAGATGTAAATATTTTATTTAATATATATATATTGTTATATATTATTAATATTTAAATTTATTTATCTATTATATTGA : 659
AB-i1-c14-R : ATTGAATAGAAGAGATATAATAATAATTATGTGTTAGATGTAAATATTTTATTTAATATATATATATTGTTATATATTATTAATATTTAAATTTATTTATCTATTATATTGA : 659
AB-i1-c15-F : ATTGAATAGAAGAGATATAATAATAATTATGTGTTAGATGTAAATATTTTATTTAATATATATATATTGTTATATATTATTAATATTTAAATTTATTTATCTATTATATTGA : 659
AB-i1-c15-R : ATTGAATAGAAGAGATATAATAATAATTATGTGTTAGATGTAAATATTTTATTTAATATATATATATTGTTATATATTATTAATATTTAAATTTATTTATCTATTATATTGA : 659
AB-i1-c16-F : ATTGAATAGAAGAGATATAATAATAATTATGTGTTAGATGTAAATATTTTATTTAATATATATATATTGTTATATATTATTAATATTTAAATTTATTTATCTATTATATTGA : 659
AB-i1-c16-R : ATTGAATAGAAGAGATATAATAATAATTATGTGTTAGATGTAAATATTTTATTTAATATATATATATTGTTATATATTATTAATATTTAAATTTATTTATCTATTATATTGA : 659
AB-i1-c17-F : ATTGAATAGAAGAGATATAATAATAATTATGTGTTAGATGTAAATATTTTATTTAATATATATATATTGTTATATATTATTAATATTTAAATTTATTTATCTATTATATTGA : 659
AB-i1-c17-R : ATTGAATAGAAGAGATATAATAATAATTATGTGTTAGATGTAAATATTTTATTTAATATATATATATTGTTATATATTATTAATATTTAAATTTATTTATCTATTATATTGA : 659
AB-i1-c18-F : ATTGAATAGAAGAGATATAATAATAATTATGTGTTAGATGTAAATATTTTATTTAATATATATATATTGTTATATATTATTAATATTTAAATTTATTTATCTATTATATTGA : 658
AB-i1-c18-R : ATTGAATAGAAGAGATATAATAATAATTATGTGTTAGATGTAAATATTTTATTTAATATATATATATTGTTATATATTATTAATATTTAAATTTATTTATCTATTATATTGA : 658
AB-i1-c19-F : ATTGAATAGAAGAGATATAATAATAATTATGTGTTAGATGTAAATATTTTATTTAATATATATATATTGTTATATATTATTAATATTTAAATTTATTTATCTATTATATTGA : 659
AB-i1-c19-R : ATTGAATAGAAGAGATATAATAATAATTATGTGTTAGATGTAAATATTTTATTTAATATATATATATTGTTATATATTATTAATATTTAAATTTATTTATCTATTATATTGA : 659
```

AB-i5-c1-F	ATTGAATAGAAGAGATATAATAATAATTATGTGTTAGATGTAAATATTTTATTTAATATATATATTGTTATATATTATTAATATTTAAATTTATTTATCTATTATATTGA	649
AB-i5-c1-R	ATTGAATAGAAGAGATATAATAATAATTATGTGTTAGATGTAAATATTTTATTTAATATATATATTGTTATATATTATTAATATTTAAATTTATTTATCTATTATATTGA	649
AB-i5-c2-F	ATTGAATAGAAGAGATATAATAATAATTATGTGTTAGATGTAAATATTTTATTTAATATATATATTGTTATATATTATTAATATTTAAATTTATTTATCTATTATATTGA	649
AB-i5-c2-R	ATTGAATAGAAGAGATATAATAATAATTATGTGTTAGATGTAAATATTTTATTTAATATATATATTGTTATATATTATTAATATTTAAATTTATTTATCTATTATATTGA	649
AB-i5-c6-F	ATTGAATAGAAGAGATATAATAATAATTATGTGTTAGATGTAAATATTTTATTTAATATATATATTGTTATATATTATTAATATTTAAATTTATTTATCTATTATATTGA	649
AB-i5-c6-R	ATTGAATAGAAGAGATATAATAATAATTATGTGTTAGATGTAAATATTTTATTTAATATATATATTGTTATATATTATTAATATTTAAATTTATTTATCTATTATATTGA	649
AB-i5-c7-F	ATTGAATAGAAGAGATATAATAATAATTATGTGTTAGATGTAAATATTTTATTTAATATATATATTGTTATATATTATTAATATTTAAATTTATTTATCTATTATATTGA	649
AB-i5-c7-R	ATTGAATAGAAGAGATATAATAATAATTATGTGTTAGATGTAAATATTTTATTTAATATATATATTGTTATATATTATTAATATTTAAATTTATTTATCTATTATATTGA	649
AB-i5-c11-F	ATTGAATAGAAGAGATATAATAATAATTATGTGTTAGATGTAAATATTTTATTTAATATATATATTGTTATATATTATTAATATTTAAATTTATTTATCTATTATATTGA	649
AB-i5-c11-R	ATTGAATAGAAGAGATATAATAATAATTATGTGTTAGATGTAAATATTTTATTTAATATATATATTGTTATATATTATTAATATTTAAATTTATTTATCTATTATATTGA	649
AB-i5-c12-F	ATTGAATAGAAGAGATATAATAATAATTATGTGTTAGATGTAAATATTTTATTTAATATATATATTGTTATATATTATTAATATTTAAATTTATTTATCTATTATATTGA	649
AB-i5-c12-R	ATTGAATAGAAGAGATATAATAATAATTATGTGTTAGATGTAAATATTTTATTTAATATATATATTGTTATATATTATTAATATTTAAATTTATTTATCTATTATATTGA	649
AB-i5-c13-F	ATTGAATAGAAGAGATATAATAATAATTATGTGTTAGATGTAAATATTTTATTTAATATATATATTGTTATATATTATTAATATTTAAATTTATTTATCTATTATATTGA	648
AB-i5-c13-R	ATTGAATAGAAGAGATATAATAATAATTATGTGTTAGATGTAAATATTTTATTTAATATATATATTGTTATATATTATTAATATTTAAATTTATTTATCTATTATATTGA	648
AB-i5-c14-F	ATTGAATAGAAGAGATATAATAATAATTATGTGTTAGATGTAAATATTTTATTTAATATATATATTGTTATATATTATTAATATTTAAATTTATTTATCTATTATATTGA	649
AB-i5-c14-R	ATTGAATAGAAGAGATATAATAATAATTATGTGTTAGATGTAAATATTTTATTTAATATATATATTGTTATATATTATTAATATTTAAATTTATTTATCTATTATATTGA	649
AB-i5-c15-F	ATTGAATAGAAGAGATATAATAATAATTATGTGTTAGATGTAAATATTTTATTTAATATATATATTGTTATATATTATTAATATTTAAATTTATTTATCTATTATATTGA	649
AB-i5-c15-R	ATTGAATAGAAGAGATATAATAATAATTATGTGTTAGATGTAAATATTTTATTTAATATATATATTGTTATATATTATTAATATTTAAATTTATTTATCTATTATATTGA	649
AB-i6-c4-F	ATTGAATAGAAGAGATATAATAATAATTATGTGTTAGATGTAAATATTTTATTTAATATATATATTGTTATATATTATTAATATTTAAATTTATTTATCTATTATATTGA	649
AB-i6-c4-R	ATTGAATAGAAGAGATATAATAATAATTATGTGTTAGATGTAAATATTTTATTTAATATATATATTGTTATATATTATTAATATTTAAATTTATTTATCTATTATATTGA	649
AB-i6-c7-F	ATTGAATAGAAGAGATATAATAATAATTATGTGTTAGATGTAAATATTTTATTTAATATATATATTGTTATATATTATTAATATTTAAATTTATTTATCTATTATATTGA	649
AB-i6-c7-R	ATTGAATAGAAGAGATATAATAATAATTATGTGTTAGATGTAAATATTTTATTTAATATATATATTGTTATATATTATTAATATTTAAATTTATTTATCTATTATATTGA	649
AB-i6-c8-F	ATTGAATAGAAGAGATATAATAATAATTATGTGTTAGATGTAAATATTTTATTTAATATATATATTGTTATATATTATTAATATTTAAATTTATTTATCTATTATATTGA	650
AB-i6-c8-R	ATTGAATAGAAGAGATATAATAATAATTATGTGTTAGATGTAAATATTTTATTTAATATATATATTGTTATATATTATTAATATTTAAATTTATTTATCTATTATATTGA	650
AB-i6-c9-F	ATTGAATAGAAGAGATATAATAATAATTATGTGTTAGATGTAAATATTTTATTTAATATATATATTGTTATATATTATTAATATTTAAATTTATTTATCTATTATATTGA	649
AB-i6-c9-R	ATTGAATAGAAGAGATATAATAATAATTATGTGTTAGATGTAAATATTTTATTTAATATATATATTGTTATATATTATTAATATTTAAATTTATTTATCTATTATATTGA	649
AB-i6-c11-F	ATTGAATAGAAGAGATATAATAATAATTATGTGTTAGATGTAAATATTTTATTTAATATATATATTGTTATATATTATTAATATTTAAATTTATTTATCTATTATATTGA	647
AB-i6-c11-R	ATTGAATAGAAGAGATATAATAATAATTATGTGTTAGATGTAAATATTTTATTTAATATATATATTGTTATATATTATTAATATTTAAATTTATTTATCTATTATATTGA	647
AB-i6-c12-F	ATTGAATAGAAGAGATATAATAATAATTATGTGTTAGATGTAAATATTTTATTTAATATATATATTGTTATATATTATTAATATTTAAATTTATTTATCTATTATATTGA	649
AB-i6-c12-R	ATTGAATAGAAGAGATATAATAATAATTATGTGTTAGATGTAAATATTTTATTTAATATATATATTGTTATATATTATTAATATTTAAATTTATTTATCTATTATATTGA	649
AB-i6-c17-F	ATTGAATAGAAGAGATATAATAATAATTATGTGTTAGATGTAAATATTTTATTTAATATATATATTGTTATATATTATTAATATTTAAATTTATTTATCTATTATATTGA	649
AB-i6-c17-R	ATTGAATAGAAGAGATATAATAATAATTATGTGTTAGATGTAAATATTTTATTTAATATATATATTGTTATATATTATTAATATTTAAATTTATTTATCTATTATATTGA	649
AB-i7-c11-F	ATTGAATAGAAGAGATATAATAATAATTATGTGTTAGATGTAAATATTTTATTTAATATATATATTGTTATATATTATTAATATTTAAATTTATTTATCTATTATATTGA	659
AB-i7-c11-R	ATTGAATAGAAGAGATATAATAATAATTATGTGTTAGATGTAAATATTTTATTTAATATATATATTGTTATATATTATTAATATTTAAATTTATTTATCTATTATATTGA	659
AB-i7-c12-F	ATTGAATAGAAGAGATATAATAATAATTATGTGTTAGATGTAAATATTTTATTTAATATATATATTGTTATATATTATTAATATTTAAATTTATTTATCTATTATATTGA	659
AB-i7-c12-R	ATTGAATAGAAGAGATATAATAATAATTATGTGTTAGATGTAAATATTTTATTTAATATATATATTGTTATATATTATTAATATTTAAATTTATTTATCTATTATATTGA	659
AB-i7-c13-F	ATTGAATAGAAGAGATATAATAATAATTATGTGTTAGATGTAAATATTTTATTTAATATATATATTGTTATATATTATTAATATTTAAATTTATTTATCTATTATATTGA	649
AB-i7-c13-R	ATTGAATAGAAGAGATATAATAATAATTATGTGTTAGATGTAAATATTTTATTTAATATATATATTGTTATATATTATTAATATTTAAATTTATTTATCTATTATATTGA	649
AB-i7-c14-F	ATTGAATAGAAGAGATATAATAATAATTATGTGTTAGATGTAAATATTTTATTTAATATATATATTGTTATATATTATTAATATTTAAATTTATTTATCTATTATATTGA	659
AB-i7-c14-R	ATTGAATAGAAGAGATATAATAATAATTATGTGTTAGATGTAAATATTTTATTTAATATATATATTGTTATATATTATTAATATTTAAATTTATTTATCTATTATATTGA	659
AB-i7-c16-F	ATTGAATAGAAGAGATATAATAATAATTATGTGTTAGATGTAAATATTTTATTTAATATATATATTGTTATATATTATTAATATTTAAATTTATTTATCTATTATATTGA	659
AB-i7-c16-R	ATTGAATAGAAGAGATATAATAATAATTATGTGTTAGATGTAAATATTTTATTTAATATATATATTGTTATATATTATTAATATTTAAATTTATTTATCTATTATATTGA	659
AB-i7-c18-F	ATTGAATAGAAGAGATATAATAATAATTATGTGTTAGATGTAAATATTTTATTTAATATATATATTGTTATATATTATTAATATTTAAATTTATTTATCTATTATATTGA	648
AB-i7-c18-R	ATTGAATAGAAGAGATATAATAATAATTATGTGTTAGATGTAAATATTTTATTTAATATATATATTGTTATATATTATTAATATTTAAATTTATTTATCTATTATATTGA	648
AB-i7-c19-F	ATTGAATAGAAGAGATATAATAATAATTATGTGTTAGATGTAAATATTTTATTTAATATATATATTGTTATATATTATTAATATTTAAATTTATTTATCTATTATATTGA	658
AB-i7-c19-R	ATTGAATAGAAGAGATATAATAATAATTATGTGTTAGATGTAAATATTTTATTTAATATATATATTGTTATATATTATTAATATTTAAATTTATTTATCTATTATATTGA	658

```
AB-i7-c20-F : ATTGAATAGAAGAGATATAATAATAATTATGTGTTAGATGTAAATATTTTATTTAATATATATATTGTTATATATTATTAATATTTAAATTTATTTATCTATTATATTGA : 659
AB-i7-c20-R : ATTGAATAGAAGAGATATAATAATAATTATGTGTTAGATGTAAATATTTTATTTAATATATATATTGTTATATATTATTAATATTTAAATTTATTTATCTATTATATTGA : 659
              ATTGAATAGAAGAGATATAATAATAATTATGTGTTAGATGTAAATATTTTATTTAATATATATATTGTTATATATTATTAATATTTAAATTTATTTATCTATTATATTGA

                      *       680         *       700         *       720         *       740         *       760         *
AB          : ATTTTTAGAGATGAAAACTATAGTTATGAAGAGCTAATTAATAAACAATTAATAAGTAATATCCCAGTAGTTTATGAAAAAATTCATATGCATGCACAATTTGTGTTATG : 759
AB-i1-c4-F  : ATTTTTAGAGATGAAAACTATAGTTATGAAGAGCTAATTAATAAACAATTAATAAGTAATATCCCAGTAGTTTATGAAAAAATTCATATGCATGCACAATTTGTGTTATG : 769
AB-i1-c4-R  : ATTTTTAGAGATGAAAACTATAGTTATGAAGAGCTAATTAATAAACAATTAATAAGTAATATCCCAGTAGTTTATGAAAAAATTCATATGCATGCACAATTTGTGTTATG : 769
AB-i1-c11-F : ATTTTTAGAGATGAAAACTATAGTTATGAAGAGCTAATTAATAAACAATTAATAAGTAATATCCCAGTAGTTTATGAAAAAATTCATATGCATGCACAATTTGTGTTATG : 769
AB-i1-c11-R : ATTTTTAGAGATGAAAACTATAGTTATGAAGAGCTAATTAATAAACAATTAATAAGTAATATCCCAGTAGTTTATGAAAAAATTCATATGCATGCACAATTTGTGTTATG : 769
AB-i1-c14-F : ATTTTTAGAGATGAAAACTATAGTTATGAAGAGCTAATTAATAAACAATTAATAAGTAATATCCCAGTAGTTTATGAAAAAATTCATATGCATGCACAATTTGTGTTATG : 769
AB-i1-c14-R : ATTTTTAGAGATGAAAACTATAGTTATGAAGAGCTAATTAATAAACAATTAATAAGTAATATCCCAGTAGTTTATGAAAAAATTCATATGCATGCACAATTTGTGTTATG : 769
AB-i1-c15-F : ATTTTTAGAGATGAAAACTATAGTTATGAAGAGCTAATTAATAAACAATTAATAAGTAATATCCCAGTAGTTTATGAAAAAATTCATATGCATGCACAATTTGTGTTATG : 769
AB-i1-c15-R : ATTTTTAGAGATGAAAACTATAGTTATGAAGAGCTAATTAATAAACAATTAATAAGTAATATCCCAGTAGTTTATGAAAAAATTCATATGCATGCACAATTTGTGTTATG : 769
AB-i1-c16-F : ATTTTTAGAGATGAAAACTATAGTTATGAAGAGCTAATTAATAAACAATTAATAAGTAATATCCCAGTAGTTTATGAAAAAATTCATATGCATGCACAATTTGTGTTATG : 769
AB-i1-c16-R : ATTTTTAGAGATGAAAACTATAGTTATGAAGAGCTAATTAATAAACAATTAATAAGTAATATCCCAGTAGTTTATGAAAAAATTCATATGCATGCACAATTTGTGTTATG : 769
AB-i1-c17-F : ATTTTTAGAGATGAAAACTATAGTTATGAAGAGCTAATTAATAAACAATTAATAAGTAATATCCCAGTAGTTTATGAAAAAATTCATATGCATGCACAATTTGTGTTATG : 769
AB-i1-c17-R : ATTTTTAGAGATGAAAACTATAGTTATGAAGAGCTAATTAATAAACAATTAATAAGTAATATCCCAGTAGTTTATGAAAAAATTCATATGCATGCACAATTTGTGTTATG : 769
AB-i1-c18-F : ATTTTTAGAGATGAAAACTATAGTTATGAAGAGCTAATTAATAAACAATTAATAAGTAATATCCCAGTAGTTTATGAAAAAATTCATATGCATGCACAATTTGTGTTATG : 768
AB-i1-c18-R : ATTTTTAGAGATGAAAACTATAGTTATGAAGAGCTAATTAATAAACAATTAATAAGTAATATCCCAGTAGTTTATGAAAAAATTCATATGCATGCACAATTTGTGTTATG : 768
AB-i1-c19-F : ATTTTTAGAGATGAAAACTATAGTTATGAAGAGCTAATTAATAAACAATTAATAAGTAATATCCCAGTAGTTTATGAAAAAATTCATATGCATGCACAATTTGTGTTATG : 769
AB-i1-c19-R : ATTTTTAGAGATGAAAACTATAGTTATGAAGAGCTAATTAATAAACAATTAATAAGTAATATCCCAGTAGTTTATGAAAAAATTCATATGCATGCACAATTTGTGTTATG : 769
AB-i5-c1-F  : ATTTTTAGAGATGAAAACTATAGTTATGAAGAGCTAATTAATAAACAATTAATAAGTAATATCCCAGTAGTTTATGAAAAAATTCATATGCATGCACAATTTGTGTTATG : 759
AB-i5-c1-R  : ATTTTTAGAGATGAAAACTATAGTTATGAAGAGCTAATTAATAAACAATTAATAAGTAATATCCCAGTAGTTTATGAAAAAATTCATATGCATGCACAATTTGTGTTATG : 759
AB-i5-c2-F  : ATTTTTAGAGATGAAAACTATAGTTATGAAGAGCTAATTAATAAACAATTAATAAGTAATATCCCAGTAGTTTATGAAAAAATTCATATGCATGCACAATTTGTGTTATG : 759
AB-i5-c2-R  : ATTTTTAGAGATGAAAACTATAGTTATGAAGAGCTAATTAATAAACAATTAATAAGTAATATCCCAGTAGTTTATGAAAAAATTCATATGCATGCACAATTTGTGTTATG : 759
AB-i5-c6-F  : ATTTTTAGAGATGAAAACTATAGTTATGAAGAGCTAATTAATAAACAATTAATAAGTAATATCCCAGTAGTTTATGAAAAAATTCATATGCATGCACAATTTGTGTTATG : 759
AB-i5-c6-R  : ATTTTTAGAGATGAAAACTATAGTTATGAAGAGCTAATTAATAAACAATTAATAAGTAATATCCCAGTAGTTTATGAAAAAATTCATATGCATGCACAATTTGTGTTATG : 759
AB-i5-c7-F  : ATTTTTAGAGATGAAAACTATAGTTATGAAGAGCTAATTAATAAACAATTAATAAGTAATATCCCAGTAGTTTATGAAAAAATTCATATGCATGCACAATTTGTGTTATG : 759
AB-i5-c7-R  : ATTTTTAGAGATGAAAACTATAGTTATGAAGAGCTAATTAATAAACAATTAATAAGTAATATCCCAGTAGTTTATGAAAAAATTCATATGCATGCACAATTTGTGTTATG : 759
AB-i5-c11-F : ATTTTTAGAGATGAAAACTATAGTTATGAAGAGCTAATTAATAAACAATTAATAAGTAATATCCCAGTAGTTTATGAAAAAATTCATATGCATGCACAATTTGTGTTATG : 759
AB-i5-c11-R : ATTTTTAGAGATGAAAACTATAGTTATGAAGAGCTAATTAATAAACAATTAATAAGTAATATCCCAGTAGTTTATGAAAAAATTCATATGCATGCACAATTTGTGTTATG : 759
AB-i5-c12-F : ATTTTTAGAGATGAAAACTATAGTTATGAAGAGCTAATTAATAAACAATTAATAAGTAATATCCCAGTAGTTTATGAAAAAATTCATATGCATGCACAATTTGTGTTATG : 759
AB-i5-c12-R : ATTTTTAGAGATGAAAACTATAGTTATGAAGAGCTAATTAATAAACAATTAATAAGTAATATCCCAGTAGTTTATGAAAAAATTCATATGCATGCACAATTTGTGTTATG : 759
AB-i5-c13-F : ATTTTTAGAGATGAAAACTATAGTTATGAAGAGCTAATTAATAAACAATTAATAAGTAATATCCCAGTAGTTTATGAAAAAATTCATATGCATGCACAATTTGTGTTATG : 758
AB-i5-c13-R : ATTTTTAGAGATGAAAACTATAGTTATGAAGAGCTAATTAATAAACAATTAATAAGTAATATCCCAGTAGTTTATGAAAAAATTCATATGCATGCACAATTTGTGTTATG : 758
AB-i5-c14-F : ATTTTTAGAGATGAAAACTATAGTTATGAAGAGCTAATTAATAAACAATTAATAAGTAATATCCCAGTAGTTTATGAAAAAATTCATATGCATGCACAATTTGTGTTATG : 759
AB-i5-c14-R : ATTTTTAGAGATGAAAACTATAGTTATGAAGAGCTAATTAATAAACAATTAATAAGTAATATCCCAGTAGTTTATGAAAAAATTCATATGCATGCACAATTTGTGTTATG : 759
AB-i5-c15-F : ATTTTTAGAGATGAAAACTATAGTTATGAAGAGCTAATTAATAAACAATTAATAAGTAATATCCCAGTAGTTTATGAAAAAATTCATATGCATGCACAATTTGTGTTATG : 759
AB-i5-c15-R : ATTTTTAGAGATGAAAACTATAGTTATGAAGAGCTAATTAATAAACAATTAATAAGTAATATCCCAGTAGTTTATGAAAAAATTCATATGCATGCACAATTTGTGTTATG : 759
AB-i6-c4-F  : ATTTTTAGAGATGAAAACTATAGTTATGAAGAGCTAATTAATAAACAATTAATAAGTAATATCCCAGTAGTTTATGAAAAAATTCATATGCATGCACAATTTGTGTTATG : 759
AB-i6-c4-R  : ATTTTTAGAGATGAAAACTATAGTTATGAAGAGCTAATTAATAAACAATTAATAAGTAATATCCCAGTAGTTTATGAAAAAATTCATATGCATGCACAATTTGTGTTATG : 759
AB-i6-c7-F  : ATTTTTAGAGATGAAAACTATAGTTATGAAGAGCTAATTAATAAACAATTAATAAGTAATATCCCAGTAGTTTATGAAAAAATTCATATGCATGCACAATTTGTGTTATG : 759
AB-i6-c7-R  : ATTTTTAGAGATGAAAACTATAGTTATGAAGAGCTAATTAATAAACAATTAATAAGTAATATCCCAGTAGTTTATGAAAAAATTCATATGCATGCACAATTTGTGTTATG : 759
AB-i6-c8-F  : ATTTTTAGAGATGAAAACTATAGTTATGAAGAGCTAATTAATAAACAATTAATAAGTAATATCCCAGTAGTTTATGAAAAAATTCATATGCATGCACAATTTGTGTTATG : 760
AB-i6-c8-R  : ATTTTTAGAGATGAAAACTATAGTTATGAAGAGCTAATTAATAAACAATTAATAAGTAATATCCCAGTAGTTTATGAAAAAATTCATATGCATGCACAATTTGTGTTATG : 760
```

```
AB-i6-c9-F  : ATTTTTAGAGATGAAAACTATAGTTATGAAGAGCTAATTAATAAACAATTAATAAGTAATATCCCAGTAGTTTATGAAAAAATTCATATGCATGCACAATTTGTGTTATG : 759
AB-i6-c9-R  : ATTTTTAGAGATGAAAACTATAGTTATGAAGAGCTAATTAATAAACAATTAATAGGTAATATCCCAGTAGTTTATGAAAAAATTCATATGCATGCACAATTTGTGTTATG : 759
AB-i6-c11-F : ATTTTTAGAGATGAAAACTATAGTTATGAAGAGCTAATTAATAAACAATTAATAAGTAATATCCCAGTAGTTTATGAAAAAATTCATATGCATGCACAATTTGTGTTATG : 757
AB-i6-c11-R : ATTTTTAGAGATGAAAACTATAGTTATGAAGAGCTAATTAATAAACAATTAATAAGTAATATCCCAGTAGTTTATGAAAAAATTCATATGCATGCACAATTTGTGTTATG : 757
AB-i6-c12-F : ATTTTTAGAGATGAAAACTATAGTTATGAAGAGCTAATTAATAAACAATTAATAAGTAATATCCCAGTAGTTTATGAAAAAATTCATATGCATGCACAATTTGTGTTATG : 759
AB-i6-c12-R : ATTTTTAGAGATGAAAACTATAGTTATGAAGAGCTAATTAATAAACAATTAATAAGTAATATCCCAGTAGTTTATGAAAAAATTCATATGCATGCACAATTTGTGTTATG : 759
AB-i6-c17-F : ATTTTTAGAGATGAAAACTATAGTTATGAAGAGCTAATTAATAAACAATTAATAAGTAATATCCCAGTAGTTTATGAAAAAATTCATATGCATGCACAATTTGTGTTATG : 759
AB-i6-c17-R : ATTTTTAGAGATGAAAACTATAGTTATGAAGAGCTAATTAATAAACAATTAATAAGTAATATCCCAGTAGTTTATGAAAAAATTCATATGCATGCACAATTTGTGTTATG : 759
AB-i7-c11-F : ATTTTTAGAGATGAAAACTATAGTTATGAAGAGCTAATTAATAAACAATTAATAAGTAATATCCCAGTAGTTTATGAAAAAATTCATATGCATGCACAATTTGTGTTATG : 769
AB-i7-c11-R : ATTTTTAGAGATGAAAACTATAGTTATGAAGAGCTAATTAATAAACAATTAATAAGTAATATCCCAGTAGTTTATGAAAAAATTCATATGCATGCACAATTTGTGTTATG : 769
AB-i7-c12-F : ATTTTTAGAGATGAAAACTATAGTTATGAAGAGCTAATTAATAAACAATTAATAAGTAATATCCCAGTAGTTTATGAAAAAATTCATATGCATGCACAATTTGTGTTATG : 769
AB-i7-c12-R : ATTTTTAGAGATGAAAACTATAGTTATGAAGAGCTAATTAATAAACAATTAATAAGTAATATCCCAGTAGTTTATGAAAAAATTCATATGCATGCACAATTTGTGTTATG : 769
AB-i7-c13-F : ATTTTTAGAGATGAAAACTATAGTTATGAAGAGCTAATTAATAAACAATTAATAAGTAATATCCCAGTAGTTTATGAAAAAATTCATATGCATGCACAATTTGTGTTATG : 759
AB-i7-c13-R : ATTTTTAGAGATGAAAACTATAGTTATGAAGAGCTAATTAATAAACAATTAATAAGTAATATCCCAGTAGTTTATGAAAAAATTCATATGCATGCACAATTTGTGTTATG : 759
AB-i7-c14-F : ATTTTTAGAGATGAAAACTATAGTTATGAAGAGCTAATTAATAAACAATTAATAAGTAATATCCCAGTAGTTTATGAAAAAATTCATATGCATGCACAATTTGTGTTATG : 769
AB-i7-c14-R : ATTTTTAGAGATGAAAACTATAGTTATGAAGAGCTAATTAATAAACAATTAATAAGTAATATCCCAGTAGTTTATGAAAAAATTCATATGCATGCACAATTTGTGTTATG : 769
AB-i7-c16-F : ATTTTTAGAGATGAAAACTATAGTTATGAAGAGCTAATTAATAAACAATTAATAAGTAATATCCCAGTAGTTTATGAAAAAATTCATATGCATGCACAATTTGTGTTATG : 769
AB-i7-c16-R : ATTTTTAGAGATGAAAACTATAGTTATGAAGAGCTAATTAATAAACAATTAATAAGTAATATCCCAGTAGTTTATGAAAAAATTCATATGCATGCACAATTTGTGTTATG : 769
AB-i7-c18-F : ATTTTTAGAGATGAAAACTATAGTTATGAAGAGCTAATTAATAAACAATTAATAAGTAATATCCCAGTAGTTTATGAAAAAATTCATATGCATGCACAATTTGTGTTATG : 758
AB-i7-c18-R : ATTTTTAGAGATGAAAACTATAGTTATGAAGAGCTAATTAATAAACAATTAATAAGTAATATCCCAGTAGTTTATGAAAAAATTCATATGCATGCACAATTTGTGTTATG : 758
AB-i7-c19-F : ATTTTTAGAGATGAAAACTATAGTTATGAAGAGCTAATTAATAAACAATTAATAAGTAATATCCCAGTAGTTTATGAAAAAATTCATATGCATGCACAATTTGTGTTATG : 768
AB-i7-c19-R : ATTTTTAGAGATGAAAACTATAGTTATGAAGAGCTAATTAATAAACAATTAATAAGTAATATCCCAGTAGTTTATGAAAAAATTCATATGCATGCACAATTTGTGTTATG : 768
AB-i7-c20-F : ATTTTTAGAGATGAAAACTATAGTTATGAAGAGCTAATTAATAAACAATTAATAAGTAATATCCCAGTAGTTTATGAAAAAATTCATATGCATGCACAATTTGTGTTATG : 769
AB-i7-c20-R : ATTTTTAGAGATGAAAACTATAGTTATGAAGAGCTAATTAATAAACAATTAATAAGTAATATCCCAGTAGTTTATGAAAAAATTCATATGCATGCACAATTTGTGTTATG : 769
              ATTTTTAGAGATGAAAACTATAGTTATGAAGAGCTAATTAATAAACAATTAATAaGTAATATCCCAGTAGTTTATGAAAAAATTCATATGCATGCACAATTTGTGTTATG
```

```
                  780
AB          : TGAGCCCGCTTAG : 772
AB-i1-c4-F  : TGAGCCCGCTTAG : 782
AB-i1-c4-R  : TGAGCCCGCTTAG : 782
AB-i1-c11-F : TGAGCCCGCTTAG : 782
AB-i1-c11-R : TGAGCCCGCTTAG : 782
AB-i1-c14-F : TGAGCCCGCTTAG : 782
AB-i1-c14-R : TGAGCCCGCTTAG : 782
AB-i1-c15-F : TGAGCCCGCTTAG : 782
AB-i1-c15-R : TGAGCCCGCTTAG : 782
AB-i1-c16-F : TGAGCCCGCTTAG : 782
AB-i1-c16-R : TGAGCCCGCTTAG : 782
AB-i1-c17-F : -GAGCCCGCTTAG : 781
AB-i1-c17-R : -GAGCCCGCTTAG : 781
AB-i1-c18-F : TGAGCCCGCTTAG : 781
AB-i1-c18-R : TGAGCCCGCTTAG : 781
AB-i1-c19-F : TGAGCCCGCTTAG : 782
AB-i1-c19-R : TGAGCCCGCTTAG : 782
AB-i5-c1-F  : TGAGCCCGCTTAG : 772
AB-i5-c1-R  : TGAGCCCGCTTAG : 772
```

```
AB-i5-c2-F  : TGAGCCCGCTTAG : 772
AB-i5-c2-R  : TGAGCCCGCTTAG : 772
AB-i5-c6-F  : TGAGCCCGCTTAG : 772
AB-i5-c6-R  : TGAGCCCGCTTAG : 772
AB-i5-c7-F  : TGAGCCCGCTTA- : 771
AB-i5-c7-R  : TGAGCCCGCTTA- : 771
AB-i5-c11-F : TGAGCCCGCTTAG : 772
AB-i5-c11-R : TGAGCCCGCTTAG : 772
AB-i5-c12-F : TGAGCCCGCTTAG : 772
AB-i5-c12-R : TGAGCCCGCTTAG : 772
AB-i5-c13-F : TGAGCCCGCTTAG : 771
AB-i5-c13-R : TGAGCCCGCTTAG : 771
AB-i5-c14-F : TGAGCCCGCTTAG : 772
AB-i5-c14-R : TGAGCCCGCTTAG : 772
AB-i5-c15-F : TGAGCCCGCTTAG : 772
AB-i5-c15-R : TGAGCCCGCTTAG : 772
AB-i6-c4-F  : TGAGCCCGCTTAG : 772
AB-i6-c4-R  : TGAGCCCGCTTAG : 772
AB-i6-c7-F  : TGAGCCCGCTTAG : 772
AB-i6-c7-R  : TGAGCCCGCTTAG : 772
AB-i6-c8-F  : TGAGCCCGCTTAG : 773
AB-i6-c8-R  : TGAGCCCGCTTAG : 773
AB-i6-c9-F  : TGAGCCCGCTTAG : 772
AB-i6-c9-R  : TGAGCCCGCTTAG : 772
AB-i6-c11-F : TGAGCCCGCTTAG : 770
AB-i6-c11-R : TGAGCCCGCTTAG : 770
AB-i6-c12-F : TGAGCCCGCTTAG : 772
AB-i6-c12-R : TGAGCCCGCTTAG : 772
AB-i6-c17-F : TGAGCCCGCTTAG : 772
AB-i6-c17-R : TGAGCCCGCTTAG : 772
AB-i7-c11-F : TGAGCCCGCTTAG : 782
AB-i7-c11-R : TGAGCCCGCTTAG : 782
AB-i7-c12-F : TGAGCCCGCTTAG : 782
AB-i7-c12-R : TGAGCCCGCTTAG : 782
AB-i7-c13-F : TGAGCCCGCTTAG : 772
AB-i7-c13-R : TGAGCCCGCTTAG : 772
AB-i7-c14-F : TGAGCCCGCTTAG : 782
AB-i7-c14-R : TGAGCCCGCTTAG : 782
AB-i7-c16-F : TGAGCCCGCTTA- : 781
AB-i7-c16-R : TGAGCCCGCTTA- : 781
AB-i7-c18-F : TGAGCCCGCTTAG : 771
AB-i7-c18-R : TGAGCCCGCTTAG : 771
AB-i7-c19-F : TGAGCCCGCTTAG : 781
AB-i7-c19-R : TGAGCCCGCTTAG : 781
AB-i7-c20-F : TGAGCCCGCTTAG : 782
AB-i7-c20-R : TGAGCCCGCTTAG : 782
```

tGAGCCCGCTTAg

(B) Region BC

```
                       *        20         *        40         *        60         *        80         *       100         *
BC          : -AAAGGGCAGAGTGAATAGAAATACAGACCAAGCTAAAGATATTAAGCATAACAAAAATGTTGTTCTTTAAAAAAATGAATTGTATTTTTGCTTTTTTTTTTTTATTGTA : 109
BC-i1-c11-F : -AAAGGGCAGAGTGAATAGAAATACAGACCAAGCTAAAGATATTAAGCATAACAAAAATGTTGTTCTTTAAAAAAATGAATTGTATTTTTGCTTTTTTTTTTTTATTGTA : 109
BC-i1-c11-R : -AAAGGGCAGAGTGAATAGAAATACAGACCAAGCTAAAGATATTAAGCATAACAAAAATGTTGTTCTTTAAAAAAATGAATTGTATTTTTGCTTTTTTTTTTTTATTGTA : 109
BC-i1-c12-F : --AAGGGCAGAGTGAATAGAAATACAGACCAAGCTAAAGATATTAAGCATAACAAAAATGTTGTTCTTTAAAAAAATGAATTGTATTTTTGCTTTTTTTTTTTTATTGTA : 108
BC-i1-c12-R : --AAGGGCAGAGTGAATAGAAATACAGACCAAGCTAAAGATATTAAGCATAACAAAAATGTTGTTCTTTAAAAAAATGAATTGTATTTTTGCTTTTTTTTTTTTATTGTA : 108
BC-i1-c13-F : -AAAGGGCAGAGTGAATAGAAATACAGACCAAGCTAAAGATATTAAGCATAACAAAAATGTTGTTCTTTAAAAAAATGAATTGTATTTTTGCTTTTTTTTTTTTATTGTA : 109
BC-i1-c13-R : -AAAGGGCAGAGTGAATAGAAATACAGACCAAGCTAAAGATATTAAGCATAACAAAAATGTTGTTCTTTAAAAAAATGAATTGTATTTTTGCTTTTTTTTTTTTATTGTA : 109
BC-i1-c18-F : -AAAGGGCAGAGTGAATAGAAATACAGACCAAGCTAAAGATATTAAGCATAACAAAAATGTTGTTCTTTAAAAAAATGAATTGTATTTTTGCTTTTTTTTTTTTATTGTA : 109
BC-i1-c18-R : -AAAGGGCAGAGTGAATAGAAATACAGACCAAGCTAAAGATATTAAGCATAACAAAAATGTTGTTCTTTAAAAAAATGAATTGTATTTTTGCTTTTTTTTTTTTATTGTA : 109
BC-i1-c19-F : -AAAGGGCAGAGTGAATAGAAATACAGACCAAGCTAAAGATATTAAGCATAACAAAAATGTTGTTCTTTAAAAAAATGAATTGTATTTTTGCTTTTTTTTTTTTATTGTA : 109
BC-i1-c19-R : -AAAGGGCAGAGTGAATAGAAATACAGACCAAGCTAAAGATATTAAGCATAACAAAAATGTTGTTCTTTAAAAAAATGAATTGTATTTTTGCTTTTTTTTTTTTATTGTA : 109
BC-i1-c20-F : --AAGGGCAGAGTGAATAGAAATACAGACCAAGCTAAAGATATTAAGCATAACAAAAATGTTGTTCTTTAAAAAAATGAATTGTATTTTTGCTTTTTTTTTTTTATTGTA : 108
BC-i1-c20-R : --AAGGGCAGAGTGAATAGAAATACAGACCAAGCTAAAGATATTAAGCATAACAAAAATGTTGTTCTTTAAAAAAATGAATTGTATTTTTGCTTTTTTTTTTTTATTGTA : 108
BC-i5-c1-F  : ATAAGGGCAGAGTGAATAGAAATACAGACCAAGCTAAAGATATTAAGCATAACAAAAATGTTGTTCTTTAAAAAAATGAATTGTATTTTTGCTTTTTTTTTTTTATTGTA : 110
BC-i5-c1-R  : ATAAGGGCAGAGTGAATAGAAATACAGACCAAGCTAAAGATATTAAGCATAACAAAAATGTTGTTCTTTAAAAAAATGAATTGTATTTTTGCTTTTTTTTTTTTATTGTA : 110
BC-i5-c3-F  : ATAAGGGCAGAGTGAATAGAAATACAGACCAAGCTAAAGATATTAAGCATAACAAAAATGTTGTTCTTTAAAAAAATGAATTGTATTTTTGCTTTTTTTTTTTTATTGTA : 110
BC-i5-c3-R  : ATAAGGGCAGAGTGAATAGAAATACAGACCAAGCTAAAGATATTAAGCATAACAAAAATGTTGTTCTTTAAAAAAATGAATTGTATTTTTGCTTTTTTTTTTTTATTGTA : 110
BC-i5-c4-F  : ATAAGGGCAGAGTGAATAGAAATACAGACCAAGCTAAAGATATTAAGCATAACAAAAATGTTGTTCTTTAAAAAAATGAATTGTATTTTTGCTTTTTTTTTTTTATTGTA : 110
BC-i5-c4-R  : ATAAGGGCAGAGTGAATAGAAATACAGACCAAGCTAAAGATATTAAGCATAACAAAAATGTTGTTCTTTAAAAAAATGAATTGTATTTTTGCTTTTTTTTTTTTATTGTA : 110
BC-i5-c5-F  : ATAAGGGCAGAGTGAATAGAAATACAGACCAAGCTAAAGATATTAAGCATAACAAAAATGTTGTTCTTTAAAAAAATGAATTGTATTTTTGCTTTTTTTTTTTTATTGTA : 110
BC-i5-c5-R  : ATAAGGGCAGAGTGAATAGAAATACAGACCAAGCTAAAGATATTAAGCATAACAAAAATGTTGTTCTTTAAAAAAATGAATTGTATTTTTGCTTTTTTTTTTTTATTGTA : 110
BC-i5-c6-F  : ATAAGGGCAGAGTGAATAGAAATACAGACCAAGCTAAAGATATTAAGCATAACAAAAATGTTGTTCTTTAAAAAAATGAATTGTATTTTTGCTTTTTTTTTTTTATTGTA : 110
BC-i5-c6-R  : ATAAGGGCAGAGTGAATAGAAATACAGACCAAGCTAAAGATATTAAGCATAACAAAAATGTTGTTCTTTAAAAAAATGAATTGTATTTTTGCTTTTTTTTTTTTATTGTA : 110
BC-i5-c7-F  : ATAAGGGCAGAGTGAATAGAA-TACAGACCAAGCTAAAGATATTAAGCATAACAAAAATGTTGTTCTTTAAAAAAATGAATTGTATTTTTGCTTTTTTTTTTTTATTGTA : 109
BC-i5-c7-R  : ATAAGGGCAGAGTGAATAGAA-TACAGACCAAGCTAAAGATATTAAGCATAACAAAAATGTTGTTCTTTAAAAAAATGAATTGTATTTTTGCTTTTTTTTTTTTATTGTA : 109
BC-i5-c8-F  : ATAAGGGCAGAGTGAATAGAAATACAGACCAAGCTAAAGATATTAAGCATAACAAAAATGTTGTTCTTTAAAAAAATGAATTGTATTTTTGCTTTTTTTTTTTTATTGTA : 110
BC-i5-c8-R  : ATAAGGGCAGAGTGAATAGAAATACAGACCAAGCTAAAGATATTAAGCATAACAAAAATGTTGTTCTTTAAAAAAATGAATTGTATTTTTGCTTTTTTTTTTTTATTGTA : 110
BC-i5-c9-F  : -AAAGGGCAGAGTGAATAGAAATACAGACCAAGCTAAAGATATTAAGCATAACAAAAATGTTGTTCTTTAAAAAAATGAATTGTATTTTTGCTTTTTTTTTTTTATTGTA : 109
BC-i5-c9-R  : -AAAGGGCAGAGTGAATAGAAATACAGACCAAGCTAAAGATATTAAGCATAACAAAAATGTTGTTCTTTAAAAAAATGAATTGTATTTTTGCTTTTTTTTTTTTATTGTA : 109
BC-i5-c10-F : ATAAGGGCAGAGTGAATAGAAATACAGACCAAGCTAAAGATATTAAGCATAACAAAAATGTTGTTCTTTAAAAAAATGAATTGTATTTTTGCTTTTTTTTTTTTATTGTA : 110
BC-i5-c10-R : ATAAGGGCAGAGTGAATAGAAATACAGACCAAGCTAAAGATATTAAGCATAACAAAAATGTTGTTCTTTAAAAAAATGAATTGTATTTTTGCTTTTTTTTTTTTATTGTA : 110
BC-i6-c11-F : ATAAGGGCAGAGTGAATAGAAATACAGACCAAGCTAAAGATATTAAGCATAACAAAAATGTTGTTCTTTAAAAAAATGAATTGTATTTTTGCTTTTTTTTTTTTATTGTA : 110
BC-i6-c11-R : ATAAGGGCAGAGTGAATAGAAATACAGACCAAGCTAAAGATATTAAGCATAACAAAAATGTTGTTCTTTAAAAAAATGAATTGTATTTTTGCTTTTTTTTTTTTATTGTA : 110
BC-i6-c12-F : -AAAGGGCAGAGTGAATAGAAATACAGACCAAGCTAAAGATATTAAGCATAACAAAAATGTTGTTCTTTAAAAAAATGAATTGTATTTTTGCTTTTTTTTTTTTATTGTA : 109
BC-i6-c12-R : -AAAGGGCAGAGTGAATAGAAATACAGACCAAGCTAAAGATATTAAGCATAACAAAAATGTTGTTCTTTAAAAAAATGAATTGTATTTTTGCTTTTTTTTTTTTATTGTA : 109
BC-i6-c13-F : ATAAGGGCAGAGTGAATAGAAATACAGACCAAGCTAAAGATATTAAGCATAACAAAAATGTTGTTCTTTAAAAAAATGAATTGTATTTTTGCTTTTTTTTTTTTATTGTA : 110
BC-i6-c13-R : ATAAGGGCAGAGTGAATAGAAATACAGACCAAGCTAAAGATATTAAGCATAACAAAAATGTTGTTCTTTAAAAAAATGAATTGTATTTTTGCTTTTTTTTTTTTATTGTA : 110
BC-i6-c14-F : -ATAGGGCAGAGTGAATAGAAATACAGACCAAGCTAAAGATATTAAGCATAACAAAAATGTTGTTCTTTAAAAAAATGAATTGTATTTTTGCTTTTTTTTTTTTATTGTA : 109
BC-i6-c14-R : -ATAGGGCAGAGTGAATAGAAATACAGACCAAGCTAAAGATATTAAGCATAACAAAAATGTTGTTCTTTAAAAAAATGAATTGTATTTTTGCTTTTTTTTTTTTATTGTA : 109
BC-i6-c16-F : ATAAGGGCAGAGTGAATAGAAATACAGACCAAGCTAAAGATATTAAGCATAACAAAAATGTTGTTCTTTAAAAAAATGAATTGTATTTTTGCTTTTTTTTTTTTATTGTA : 110
BC-i6-c16-R : ATAAGGGCAGAGTGAATAGAAATACAGACCAAGCTAAAGATATTAAGCATAACAAAAATGTTGTTCTTTAAAAAAATGAATTGTATTTTTGCTTTTTTTTTTTTATTGTA : 110
BC-i6-c17-F : -AAAGGGCAGAGTGAATAGAAATACAGACCAAGCTAAAGATATTAAGCATAACAAAAATGTTGTTCTTTAAAAAAATGAATTGTATTTTTGCTTTTTTTTTTTTATTGTA : 109
```

```
BC-i6-c17-R : -AAAGGGCAGAGTGAATAGAAATACAGACCAAGCTAAAGATATTAAGCATAACAAAAATGTTGTTCTTTAAAAAAATGAATTGTATTTTTGCTTTTTTTTTTTTATTGTA : 109
BC-i6-c19-F : ATAAGGGCAGAGTGAATAGAAATACAGACCAAGCTAAAGATATTAAGCATAACAAAAATGTTGTTCTTTAAAAAAATGAATTGTATTTTTGCTTTTTTTTTTT-ATTGTA : 109
BC-i6-c19-R : ATAAGGGCAGAGTGAATAGAAATACAGACCAAGCTAAAGATATTAAGCATAACAAAAATGTTGTTCTTTAAAAAAATGAATTGTATTTTTGCTTTTTTTTTTT-ATTGGA : 109
BC-i7-c1-F  : ATAAGGGCAGAGTGAATAGAAATACAGACCAAGCTAAAGATATTAAGCATAACAAAAATGTTGTTCTTTAAAAAAATGAATTGTATTTTTGCTTTTTTTTTTTTATTGTA : 110
BC-i7-c1-R  : --AAGGGCAGAGTGAATAGAAATACAGACCAAGCTAAAGATATTAAGCATAACAAAAATGTTGTTCTTTAAAAAAATGAATTGTATTTTTGCTTTTTTTTTTTTATTGTA : 108
BC-i7-c2-F  : ATAAGGGCAGAGTGAATAGAAATACAGACCAAGCTAAAGATATTAAGCATAACAAAAATGTTGTTCTTTAAAAAAATGAATTGTATTTTTGCTTTTTTTTTTTTATTGTA : 110
BC-i7-c2-R  : ATAAGGGCAGAGTGAATAGAAATACAGACCAAGCTAAAGATATTAAGCATAACAAAAATGTTGTTCTTTAAAAAAATGAATTGTATTTTTGCTTTTTTTTTTTTATTGTA : 110
BC-i7-c3-F  : ATAAGGGCAGAGTGAATAGAAATACAGACCAAGCTAAAGATATTAAGCATAACAAAAATGTTGTTCTTTAAAAAAATGAATTGTATTTTTGCTTTTTTTTTTTTATTGTA : 110
BC-i7-c3-R  : ATAAGGGCAGAGTGAATAGAAATACAGACCAAGCTAAAGATATTAAGCATAACAAAAATGTTGTTCTTTAAAAAAATGAATTGTATTTTTGCTTTTTTTTTTTTATTGTA : 110
BC-i7-c4-F  : -AAAGGGCAGAGTGAATAGAAATACAGACCAAGCTAAAGATATTAAGCATAACAAAAATGTTGTTCTTTAAAAAAATGAATTGTATTTTTGCTTTTTTTTTTTTATTGTA : 109
BC-i7-c4-R  : -AAAGGGCAGAGTGAATAGAAATACAGACCAAGCTAAAGATATTAAGCATAACAAAAATGTTGTTCTTTAAAAAAATGAATTGTATTTTTGCTTTTTTTTTTTTATTGTA : 109
BC-i7-c5-F  : -AAAGGGCAGAGTGAATAGAAATACAGACCAAGCTAAAGATATTAAGCATAACAAAAATGTTGTTCTTTAAAAAAATGAATTGTATTTTTGCTTTTTTTTTTTTATTGTA : 109
BC-i7-c5-R  : -AAAGGGCAGAGTGAATAGAAATACAGACCAAGCTAAAGATATTAAGCATAACAAAAATGTTGTTCTTTAAAAAAATGAATTGTATTTTTGCTTTTTTTTTTTTATTGTA : 109
BC-i7-c6-F  : ATAAGGGCAGAGTGAATAGAAATACAGACCAAGCTAAAGATATTAAGCATAACAAAAATGTTGTTCTTTAAAAAAATGAATTGTATTTTTGCTTTTTTTTTTTTATTGTA : 110
BC-i7-c6-R  : ATAAGGGCAGAGTGAATAGAAATACAGACCAAGCTAAAGATATTAAGCATAACAAAAATGTTGTTCTTTAAAAAAATGAATTGTATTTTTGCTTTTTTTTTTTTATTGTA : 110
BC-i7-c7-F  : ATAAGGGCAGAGTGAATAGAAATACAGACCAAGCTAAAGATATTAAGCATAACAAAAATGTTGTTCTTTAAAAAAATGAATTGTATTTTTGCTTTTTTTTTTTTATTGTA : 110
BC-i7-c7-R  : ATAAGGGCAGAGTGAATAGAAATACAGACCAAGCTAAAGATATTAAGCATAACAAAAATGTTGTTCTTTAAAAAAATGAATTGTATTTTTGCTTTTTTTTTTTTATTGTA : 110
BC-i7-c8-F  : -AAAGGGCAGAGTGAATAGAAATACAGACCAAGCTAAAGATATTAAGCATAACAAAAATGTTGTTCTTTAAAAAAATGAATTGTATTTTTGCTTTTTTTTTTTTATTGTA : 109
BC-i7-c8-R  : -AAAGGGCAGAGTGAATAGAAATACAGACCAAGCTAAAGATATTAAGCATAACAAAAATGTTGTTCTTTAAAAAAATGAATTGTATTTTTGCTTTTTTTTTTTTATTGTA : 109
BC-i7-c9-F  : -AAAGGGCAGAGTGAATAGAAATACAGACCAAGCTAAAGATATTAAGCATAACAAAAATGTTGTTCTTTAAAAAAATGAATTGTATTTTTGCTTTTTTTTTTTTATTGTA : 109
BC-i7-c9-R  : -AAAGGGCAGAGTGAATAGAAATACAGACCAAGCTAAAGATATTAAGCATAACAAAAATGTTGTTCTTTAAAAAAATGAATTGTATTTTTGCTTTTTTTTTTTTATTGTA : 109
BC-i7-c10-F : ATAAGGGCAGAGTGAATAGAAATACAGACCAAGCTAAAGATATTAAGCATAACAAAAATGTTGTTCTTTAAAAAAATGAATTGTATTTTTGCTTTTTTTTTTTTATTGTA : 110
BC-i7-c10-R : --AAGGGCAGAGTGAATAGAAATACAGACCAAGCTAAAGATATTAAGCATAACAAAAATGTTGTTCTTTAAAAAAATGAATTGTATTTTTGCTTTTTTTTTTTTATTGTA : 108
                aAGGGCAGAGTGAATAGAAaTACAGACCAAGCTAAAGATATTAAGCATAACAAAAATGTTGTTCTTTAAAAAAATGAATTGTATTTTTGCTTTTTTTTTTTtATTGtA

                    120         *       140         *       160         *       180         *       200         *       220
BC          : TTTTATTATATATTATTTAATACTTTAAATTATATTTATATTATTATTATATTAGATATATTATATATATTATATGATTTAATTAATTTAATATAATTAAATTTGATTAT : 219
BC-i1-c11-F : TTTTATTATATATTATTTAATACTTTAAATTATATTTATATTATTATTATATTAGATATATTATATATATTATATGATTTAATTAATTTAATATAATTAAATTTGATTAT : 219
BC-i1-c11-R : TTTTATTATATATTATTTAATACTTTAAATTATATTTATATTATTATTATATTAGATATATTATATATATTATATGATTTAATTAATTTAATATAATTAAATTTGATTAT : 219
BC-i1-c12-F : TTTTATTATATATTATTTAATACTTTAAATTATATTTATATTATTATTATATTAGATATATTATATATATTATATGATTTAATTAATTTAATATAATTAAATTTGATTAT : 218
BC-i1-c12-R : TTTTATTATATATTATTTAATACTTTAAATTATATTTATATTATTATTATATTAGATATATTATATATATTATATGATTTAATTAATTTAATATAATTAAATTTGATTAT : 218
BC-i1-c13-F : TTTTATTATATATTATTTAATACTTTAAATTATATTTATATTATTATTATATTAGATATATTATATATATTATATGATTTAATTAATTTAATATAATTAAATTTGATTAT : 219
BC-i1-c13-R : TTTTATTATATATTATTTAATACTTTAAATTATATTTATATTATTATTATATTAGATATATTATATATATTATATGATTTAATTAATTTAATATAATTAAATTTGATTAT : 219
BC-i1-c18-F : TTTTATTATATATTATTTAATACTTTAAATTATATTTATATTATTATTATATTAGATATATTATATATATTATATGATTTAATTAATTTAATATAATTAAATTTGATTAT : 219
BC-i1-c18-R : TTTTATTATATATTATTTAATACTTTAAATTATATTTATATTATTATTATATTAGATATATTATATATATTATATGATTTAATTAATTTAATATAATTAAATTTGATTAT : 219
BC-i1-c19-F : TTTTATTATATATTATTTAATACTTTAAATTATATTTATATTATTATTATATTAGATATATTATATATATTATATGATTTAATTAATTTAATATAATTAAATTTGATTAT : 219
BC-i1-c19-R : TTTTATTATATATTATTTAATACTTTAAATTATATTTATATTATTATTATATTAGATATATTATATATATTATATGATTTAATTAATTTAATATAATTAAATTTGATTAT : 219
BC-i1-c20-F : TTTTATTATATATTATTTAATACTTTAAATTATATTTATATTATTATTATATTAGATATATTATATATATTATATGATTTAATTAATTTAATATAATTAAATTTGATTAT : 218
BC-i1-c20-R : TTTTATTATATATTATTTAATACTTTAAATTATATTTATATTATTATTATATTAGATATATTATATATATTATATGATTTAATTAATTTAATATAATTAAATTTGATTAT : 218
BC-i5-c1-F  : TTTTATTATATATTATTTAATACTTTAAATTATATTTATATTATTATTATATTAGATATATTATATATATTATATGATTTAATTAATTTAATATAATTAAATTTGATTAT : 220
BC-i5-c1-R  : TTTTATTATATATTATTTAATACTTTAAATTATATTTATATTATTATTATATTAGATATATTATATATATTATATGATTTAATTAATTTAATATAATTAAATTTGATTAT : 220
BC-i5-c3-F  : TTTTATTATATATTATTTAATACTTTAAATTATATTTATATTATTATTATATTAGATATATTATATATATTATATGATTTAATTAATTTAATATAATTAAATTTGATTAT : 220
BC-i5-c3-R  : TTTTATTATATATTATTTAATACTTTAAATTATATTTATATTATTATTATATTAGATATATTATATATATTATATGATTTAATTAATTTAATATAATTAAATTTGATTAT : 220
BC-i5-c4-F  : TTTTATTATATATTATTTAATACTTTAAATTATATTTATATTATTATTATATTAGATATATTATATATATTATATGATTTAATTAATTTAATATAATTAAATTTGATTAT : 220
BC-i5-c4-R  : TTTTATTATATATTATTTAATACTTTAAATTATATTTATATTATTATTATATTAGATATATTATATATATTATATGATTTAATTAATTTAATATAATTAAATTTGATTAT : 220
BC-i5-c5-F  : TTTTATTATATATTATTTAATACTTTAAATTATATTTATATTATTATTATATTAGATATATTATATATATTATATGATTTAATTAATTTAATATAATTAAATTTGATTAT : 220
```

```
BC-i5-c5-R   : TTTTATTATATATTATTTAATACTTTAAATTATATTTATATTATTATTATATTAGATATATTATATATATTATATGATTTAATTAATTTAATATAATTAAATTTGATTAT : 220
BC-i5-c6-F   : TTTTATTATATATTATTTAATACTTTAAATTATATTTATATTATTATTATATTAGATATATTATATATATTATATGATTTAATTAATTTAATATAATTAAATTTGATTAT : 220
BC-i5-c6-R   : TTTTATTATATATTATTTAATACTTTAAATTATATTTATATTATTATTATATTAGATATATTATATATATTATATGATTTAATTAATTTAATATAATTAAATTTGATTAT : 220
BC-i5-c7-F   : TTTTATTATATATTATTTAATACTTTAAATTATATTTATATTATTATTATATTAGATATATTATATATATTATATGATTTAATTAATTTAATATAATTAAATTTGATTAT : 219
BC-i5-c7-R   : TTTTATTATATATTATTTAATACTTTAAATTATATTTATATTATTATTATATTAGATATATTATATATATTATATGATTTAATTAATTTAATATAATTAAATTTGATTAT : 219
BC-i5-c8-F   : TTTTATTATATATTATTTAATACTTTAAATTATATTTATATTATTATTATATTAGATATATTATATATATTATATGATTTAATTAATTTAATATAATTAAATTTGATTAT : 220
BC-i5-c8-R   : TTTTATTATATATTATTTAATACTTTAAATTATATTTATATTATTATTATATTAGATATATTATATATATTATATGATTTAATTAATTTAATATAATTAAATTTGATTAT : 220
BC-i5-c9-F   : TTTTATTATATATTATTTAATACTTTAAATTATATTTATATTATTATTATATTAGATATATTATATATATTATATGATTTAATTAATTTAATATAATTAAATTTGATTAT : 219
BC-i5-c9-R   : TTTTATTATATATTATTTAATACTTTAAATTATATTTATATTATTATTATATTAGATATATTATATATATTATATGATTTAATTAATTTAATATAATTAAATTTGATTAT : 219
BC-i5-c10-F  : TTTTATTATATATTATTTAATACTTTAAATTATATTTATATTATTATTATATTAGATATATTATATATATTATATGATTTAATTAATTTAATATAATTAAATTTGATTAT : 220
BC-i5-c10-R  : TTTTATTATATATTATTTAATACTTTAAATTATATTTATATTATTATTATATTAGATATATTATATATATTATATGATTTAATTAATTTAATATAATTAAATTTGATTAT : 220
BC-i6-c11-F  : TTTTATTATATATTATTTAATACTTTAAATTATATTTATATTATTATTATATTAGATATATTATATATATTATATGATTTAATTAATTTAATATAATTAAATTTGATTAT : 220
BC-i6-c11-R  : TTTTATTATATATTATTTAATACTTTAAATTATATTTATATTATTATTATATTAGATATATTATATATATTATATGATTTAATTAATTTAATATAATTAAATTTGATTAT : 220
BC-i6-c12-F  : TTTTATTATATATTATTTAATACTTTAAATTATATTTATATTATTATTATATTAGATATATTATATATATTATATGATTTAATTAATTTAATATAATTAAATTTGATTAT : 219
BC-i6-c12-R  : TTTTATTATATATTATTTAATACTTTAAATTATATTTATATTATTATTATATTAGATATATTATATATATTATATGATTTAATTAATTTAATATAATTAAATTTGATTAT : 219
BC-i6-c13-F  : TTTTATTATATATTATTTAATACTTTAAATTATATTTATATTATTATTATATTAGATATATTATATATATTATATGATTTAATTAATTTAATATAATTAAATTTGATTAT : 220
BC-i6-c13-R  : TTTTATTATATATTATTTAATACTTTAAATTATATTTATATTATTATTATATTAGATATATTATATATATTATATGATTTAATTAATTTAATATAATTAAATTTGATTAT : 220
BC-i6-c14-F  : TTTTATTATATATTATTTAATACTTTAAATTATATTTATATTATTATTATATTAGATATATTATATATATTATATGATTTAATTAATTTAATATAATTAAATTTGATTAT : 219
BC-i6-c14-R  : TTTTATTATATATTATTTAATACTTTAAATTATATTTATATTATTATTATATTAGATATATTATATATATTATATGATTTAATTAATTTAATATAATTAAATTTGATTAT : 219
BC-i6-c16-F  : TTTTATTATATATTATTTAATACTTTAAATTATATTTATATTATTATTATATTAGATATATTATATATATTATATGATTTAATTAATTTAATATAATTAAATTTGATTAT : 220
BC-i6-c16-R  : TTTTATTATATATTATTTAATACTTTAAATTATATTTATATTATTATTATATTAGATATATTATATATATTATATGATTTAATTAATTTAATATAATTAAATTTGATTAT : 220
BC-i6-c17-F  : TTTTATTATATATTATTTAATACTTTAAATTATATTTATATTATTATTATATTAGATATATTATATATATTATATGATTTAATTAATTTAATATAATTAAATTTGATTAT : 219
BC-i6-c17-R  : TTTTATTATATATTATTTAATACTTTAAATTATATTTATATTATTATTATATTAGATATATTATATATATTATATGATTTAATTAATTTAATATAATTAAATTTGATTAT : 219
BC-i6-c19-F  : TTTTATTATATATTATTTAATACTTTAAATTATATTTATATTATTATTATATTAGATATATTATATATATTATATGATTTAATTAATTTAATATAATTAAATTTGATTAT : 219
BC-i6-c19-R  : TTTTATTATATATTATTTAATACTTTAAATTATATTTATATTATTATTATATTAGATATATTATATATATTATATGATTTAATTAATTTAATATAATTAAATTTGATTAT : 219
BC-i7-c1-F   : TTTTATTATATATTATTTAATACTTTAAATTATATTTATATTATTATTATATTAGATATATTATATATATTATATGATTTAATTAATTTAATATAATTAAATTTGATTAT : 220
BC-i7-c1-R   : TTTTATTATATATTATTTAATACTTTAAATTATATTTATATTATTATTATATTAGATATATTATATATATTATATGATTTAATTAATTTAATATAATTAAATTTGATTAT : 218
BC-i7-c2-F   : TTTTATTATATATTATTTAATACTTTAAATTATATTTATATTATTATTATATTAGATATATTATATATATTATATGATTTAATTAATTTAATATAATTAAATTTGATTAT : 220
BC-i7-c2-R   : TTTTATTATATATTATTTAATACTTTAAATTATATTTATATTATTATTATATTAGATATATTATATATATTATATGATTTAATTAATTTAATATAATTAAATTTGATTAT : 220
BC-i7-c3-F   : TTTTATTATATATTATTTAATACTTTAAATTATATTTATATTATTATTATATTAGATATATTATATATATTATATGATTTAATTAATTTAATATAATTAAATTTGATTAT : 220
BC-i7-c3-R   : TTTTATTATATATTATTTAATACTTTAAATTATATTTATATTATTATTATATTAGATATATTATATATATTATATGATTTAATTAATTTAATATAATTAAATTTGATTAT : 220
BC-i7-c4-F   : TTTTATTATATATTATTTAATACTTTAAATTATATTTATATTATTATTATATTAGATATATTATATATATTATATGATTTAATTAATTTAATATAATTAAATTTGATTAT : 219
BC-i7-c4-R   : TTTTATTATATATTATTTAATACTTTAAATTATATTTATATTATTATTATATTAGATATATTATATATATTATATGATTTAATTAATTTAATATAATTAAATTTGATTAT : 219
BC-i7-c5-F   : TTTTATTATATATTATTTAATACTTTAAATTATATTTATATTATTATTATATTAGATATATTATATATATTATATGATTTAATTAATTTAATATAATTAAATTTGATTAT : 219
BC-i7-c5-R   : TTTTATTATATATTATTTAATACTTTAAATTATATTTATATTATTATTATATTAGATATATTATATATATTATATGATTTAATTAATTTAATATAATTAAATTTGATTAT : 219
BC-i7-c6-F   : TTTTATTATATATTATTTAATACTTTAAATTATATTTATATTATTATTATATTAGATATATTATATATATTATATGATTTAATTAATTTAATATAATTAAATTTGATTAT : 220
BC-i7-c6-R   : TTTTATTATATATTATTTAATACTTTAAATTATATTTATATTATTATTATATTAGATATATTATATATATTATATGATTTAATTAATTTAATATAATTAAATTTGATTAT : 220
BC-i7-c7-F   : TTTTATTATATATTATTTAATACTTTAAATTATATTTATATTATTATTATATTAGATATATTATATATATTATATGATTTAATTAATTTAATATAATTAAATTTGATTAT : 220
BC-i7-c7-R   : TTTTATTATATATTATTTAATACTTTAAATTATATTTATATTATTATTATATTAGATATATTATATATATTATATGATTTAATTAATTTAATATAATTAAATTTGATTAT : 220
BC-i7-c8-F   : TTTTATTATATATTATTTAATACTTTAAATTATATTTATATTATTATTATATTAGATATATTATATATATTATATGATTTAATTAATTTAATATAATTAAATTTGATTAT : 219
BC-i7-c8-R   : TTTTATTATATATTATTTAATACTTTAAATTATATTTATATTATTATTATATTAGATATATTATATATATTATATGATTTAATTAATTTAATATAATTAAATTTGATTAT : 219
BC-i7-c9-F   : TTTTATTATATATTATTTAATACTTTAAATTATATTTATATTATTATTATATTAGATATATTATATATATTATATGATTTAATTAATTTAATATAATTAAATTTGATTAT : 219
BC-i7-c9-R   : TTTTATTATATATTATTTAATACTTTAAATTATATTTATATTATTATTATATTAGATATATTATATATATTATATGATTTAATTAATTTAATATAATTAAATTTGATTAT : 219
BC-i7-c10-F  : TTTTATTATATATTATTTAATACTTTAAATTATATTTATATTATTATTATATTAGATATATTATATATATTATATGATTTAATTAATTTAATATAATTAAATTTGATTAT : 220
BC-i7-c10-R  : TTTTATTATATATTATTTAATACTTTAAATTATATTTATATTATTATTATATTAGATATATTATATATATTATATGATTTAATTAATTTAATATAATTAAATTTGATTAT : 218
               TTTTATTATATATTATTTAATACTTTAAATTATATTTATATTATTATTATATTAGATATATTATATATATTATATGATTTAATTAATTTAATATAATTAAATTTGATTAT
```

```
                *       240         *       260         *       280         *       300         *       320         *
BC           : ACAAGTAGATTAAGAATTCAATATTAAAATAAAAAATTATATTATAAAGAAAGAATATATGAAATATATATATAGATTAATATTTATATTCTATATCTTTCTATTCTATA : 329
BC-i1-c11-F : ACAAGTAGATTAAGAATTCAATATTAAAATAAAAAATTATATTATAAAGAAAGAATATATGAAATATATATATAGATTAATATTTATATTCTATATCTTTCTATTCTATA : 329
BC-i1-c11-R : ACAAGTAGATTAAGAATTCAATATTAAAATAAAAAATTATATTATAAAGAAAGAATATATGAAATATATATATAGATTAATATTTATATTCTATATCTTTCTATTCTATA : 329
BC-i1-c12-F : ACAAGTAGATTAAGAATTCAATATTAAAATAAAAAATTATATTATAAAGAAAGAATATATGAAATATATATATAGATTAATATTTATATTCTATATCTTTCTATTCTATA : 328
BC-i1-c12-R : ACAAGTAGATTAAGAATTCAATATTAAAATAAAAAATTATATTATAAAGAAAGAATATATGAAATATATATATAGATTAATATTTATATTCTATATCTTTCTATTCTATA : 328
BC-i1-c13-F : ACAAGTAGATTAAGAATTCAATATTAAAATAAAAAATTATATTATAAAGAAAGAATATATGAAATATATATATAGATTAATATTTATATTCTATATCTTTCTATTCTATA : 329
BC-i1-c13-R : ACAAGTAGATTAAGAATTCAATATTAAAATAAAAAATTATATTATAAAGAAAGAATATATGAAATATATATATAGATTAATATTTATATTCTATATCTTTCTATTCTATA : 329
BC-i1-c18-F : ACAAGTAGATTAAGAATTCAATATTAAAATAAAAAATTATATTATAAAGAAAGAATATATGAAATATATATATAGATTAATATTTATATTCTATATCTTTCTATTCTATA : 329
BC-i1-c18-R : ACAAGTAGATTAAGAATTCAATATTAAAATAAAAAATTATATTATAAAGAAAGAATATATGAAATATATATATAGATTAATATTTATATTCTATATCTTTCTATTCTATA : 329
BC-i1-c19-F : ACAAGTAGATTAAGAATTCAATATTAAAATAAAAAATTATATTATAAAGAAAGAATATATGAAATATATATATAGATTAATATTTATATTCTATATCTTTCTATTCTATA : 329
BC-i1-c19-R : ACAAGTAGATTAAGAATTCAATATTAAAATAAAAAATTATATTATAAAGAAAGAATATATGAAATATATATATAGATTAATATTTATATTCTATATCTTTCTATTCTATA : 329
BC-i1-c20-F : ACAAGTAGATTAAGAATTCAATATTAAAATAAAAAATTATATTATAAAGAAAGAATATATGAAATATATATATAGATTAATATTTATATTCTATATCTTTCTATTCTATA : 328
BC-i1-c20-R : ACAAGTAGATTAAGAATTCAATATTAAAATAAAAAATTATATTATAAAGAAAGAATATATGAAATATATATATAGATTAATATTTATATTCTATATCTTTCTATTCTATA : 328
BC-i5-c1-F  : ACAAGTAGATTAAGAATTCAATATTAAAATAAAAAATTATATTATAAAGAAAGAATATATGAAATATATATATAGATTAATATTTATATTCTATATCTTTCTATTCTATA : 330
BC-i5-c1-R  : ACAAGTAGATTAAGAATTCAATATTAAAATAAAAAATTATATTATAAAGAAAGAATATATGAAATATATATATAGATTAATATTTATATTCTATATCTTTCTATTCTATA : 330
BC-i5-c3-F  : ACAAGTAGATTAAGAATTCAATATTAAAATAAAAAATTATATTATAAAGAAAGAATATATGAAATATATATATAGATTAATATTTATATTCTATATCTTTCTATTCTATA : 330
BC-i5-c3-R  : ACAAGTAGATTAAGAATTCAATATTAAAATAAAAAATTATATTATAAAGAAAGAATATATGAAATATATATATAGATTAATATTTATATTCTATATCTTTCTATTCTATA : 330
BC-i5-c4-F  : ACAAGTAGATTAAGAATTCAATATTAAAATAAAAAATTATATTATAAAGAAAGAATATATGAAATATATATATAGATTAATATTTATATTCTATATCTTTCTATTCTATA : 330
BC-i5-c4-R  : ACAAGTAGATTAAGAATTCAATATTAAAATAAAAAATTATATTATAAAGAAAGAATATATGAAATATATATATAGATTAATATTTATATTCTATATCTTTCTATTCTATA : 330
BC-i5-c5-F  : ACAAGTAGATTAAGAATTCAATATTAAAATAAAAAATTATATTATAAAGAAAGAATATATGAAATATATATATAGATTAATATTTATATTCTATATCTTTCTATTCTATA : 330
BC-i5-c5-R  : ACAAGTAGATTAAGAATTCAATATTAAAATAAAAAATTATATTATAAAGAAAGAATATATGAAATATATATATAGATTAATATTTATATTCTATATCTTTCTATTCTATA : 330
BC-i5-c6-F  : ACAAGTAGATTAAGAATTCAATATTAAAATAAAAAATTATATTATAAAGAAAGAATATATGAAATATATATATAGATTAATATTTATATTCTATATCTTTCTATTCTATA : 330
BC-i5-c6-R  : ACAAGTAGATTAAGAATTCAATATTAAAATAAAAAATTATATTATAAAGAAAGAATATATGAAATATATATATAGATTAATATTTATATTCTATATCTTTCTATTCTATA : 330
BC-i5-c7-F  : ACAAGTAGATTAAGAATTCAATATTAAAATAAAAAATTATATTATAAAGAAAGAATATATGAAATATATATATAGATTAATATTTATATTCTATATCTTTCTATTCTATA : 329
BC-i5-c7-R  : ACAAGTAGATTAAGAATTCAATATTAAAATAAAAAATTATATTATAAAGAAAGAATATATGAAATATATATATAGATTAATATTTATATTCTATATCTTTCTATTCTATA : 329
BC-i5-c8-F  : ACAAGTAGATTAAGAATTCAATATTAAAATAAAAAATTATATTATAAAGAAAGAATATATGAAATATATATATAGATTAATATTTATATTCTATATCTTTCTATTCTATA : 330
BC-i5-c8-R  : ACAAGTAGATTAAGAATTCAATATTAAAATAAAAAATTATATTATAAAGAAAGAATATATGAAATATATATATAGATTAATATTTATATTCTATATCTTTCTATTCTATA : 330
BC-i5-c9-F  : ACAAGTAGATTAAGAATTCAATATTAAAATAAAAAATTATATTATAAAGAAAGAATATATGAAATATATATATAGATTAATATTTATATTCTATATCTTTCTATTCTATA : 329
BC-i5-c9-R  : ACAAGTAGATTAAGAATTCAATATTAAAATAAAAAATTATATTATAAAGAAAGAATATATGAAATATATATATAGATTAATATTTATATTCTATATCTTTCTATTCTATA : 329
BC-i5-c10-F : ACAAGTAGATTAAGAATTCAATATTAAAATAAAAAATTATATTATAAAGAAAGAATATATGAAATATATATATAGATTAATATTTATATTCTATATCTTTCTATTCTATA : 330
BC-i5-c10-R : ACAAGTAGATTAAGAATTCAATATTAAAATAAAAAATTATATTATAAAGAAAGAATATATGAAATATATATATAGATTAATATTTATATTCTATATCTTTCTATTCTATA : 330
BC-i6-c11-F : ACAAGTAGATTAAGAATTCAATATTAAAATAAAAAATTATATTATAAAGAAAGAATATATGAAATATATATATAGATTAATATTTATATTCTATATCTTTCTATTCTATA : 330
BC-i6-c11-R : ACAAGTAGATTAAGAATTCAATATTAAAATAAAAAATTATATTATAAAGAAAGAATATATGAAATATATATATAGATTAATATTTATATTCTATATCTTTCTATTCTATA : 330
BC-i6-c12-F : ACAAGTAGATTAAGAATTCAATATTAAAATAAAAAATTATATTATAAAGAAAGAATATATGAAATATATATATAGATTAATATTTATATTCTATATCTTTCTATTCTATA : 329
BC-i6-c12-R : ACAAGTAGATTAAGAATTCAATATTAAAATAAAAAATTATATTATAAAGAAAGAATATATGAAATATATATATAGATTAATATTTATATTCTATATCTTTCTATTCTATA : 329
BC-i6-c13-F : ACAAGTAGATTAAGAATTCAATATTAAAATAAAAAATTATATTATAAAGAAAGAATATATGAAATATATATATAGATTAATATTTATATTCTATATCTTTCTATTCTATA : 330
BC-i6-c13-R : ACAAGTAGATTAAGAATTCAATATTAAAATAAAAAATTATATTATAAAGAAAGAATATATGAAATATATATATAGATTAATATTTATATTCTATATCTTTCTATTCTATA : 330
BC-i6-c14-F : ACAAGTAGATTAAGAATTCAATATTAAAATAAAAAATTATATTATAAAGAAAGAATATATGAAATATATATATAGATTAATATTTATATTCTATATCTTTCTATTCTATA : 329
BC-i6-c14-R : ACAAGTAGATTAAGAATTCAATATTAAAATAAAAAATTATATTATAAAGAAAGAATATATGAAATATATATATAGATTAATATTTATATTCTATATCTTTCTATTCTATA : 329
BC-i6-c16-F : ACAAGTAGATTAAGAATTCAATATTAAAATAAAAAATTATATTATAAAGAAAGAATATATGAAATATATATATAGATTAATATTTATATTCTATATCTTTCTATTCTATA : 330
BC-i6-c16-R : ACAAGTAGATTAAGAATTCAATATTAAAATAAAAAATTATATTATAAAGAAAGAATATATGAAATATATATATAGATTAATATTTATATTCTATATCTTTCTATTCTATA : 330
BC-i6-c17-F : ACAAGTAGATTAAGAATTCAATATTAAAATAAAAAATTATATTATAAAGAAAGAATATATGAAATATATATATAGATTAATATTTATATTCTATATCTTTCTATTCTATA : 329
BC-i6-c17-R : ACAAGTAGATTAAGAATTCAATATTAAAATAAAAAATTATATTATAAAGAAAGAATATATGAAATATATATATAGATTAATATTTATATTCTATATCTTTCTATTCTATA : 329
BC-i6-c19-F : ACAAGTAGATTAAGAATTCAATATTAAAATAAAAAATTATATTATAAAGAAAGAATATATGAAATATATATATAGATTAATATTTATATTCTATATCTTTCTATTCTATA : 329
```

```
BC-i6-c19-R : ACAAGTAGATTAAGAATTCAATATTAAAATAAAAAATTATATTATAAAGAAAGAATATATGAAATATATATATAGATTAATATTTATATTCTATATCTTTCTATTCTATA : 329
BC-i7-c1-F  : ACAAGTAGATTAAGAATTCAATATTAAAATAAAAAATTATATTATAAAGAAAGAATATATGAAATATATATATAGATTAATATTTATATTCTATATCTTTCTATTCTATA : 330
BC-i7-c1-R  : ACAAGTACATTAAGAATTCAATATTAAAATAAAAAATTATATTATAAAGAAAGAATATATGAAATATATATATAGATTAATATTTATATTCTATATCTTTCTATTCTATA : 328
BC-i7-c2-F  : ACAAGTAGATTAAGAATTCAATATTAAAATAAAAAATTATATTATAAAGAAAGAATATATGAAATATATATATAGATTAATATTTATATTCTATATCTTTCTATTCTATA : 330
BC-i7-c2-R  : ACAAGTAGATTAAGAATTCAATATTAAAATAAAAAATTATATTATAAAGAAAGAATATATGAAATATATATATAGATTAATATTTATATTCTATATCTTTCTATTCTATA : 330
BC-i7-c3-F  : ACAAGTAGATTAAGAATTCAATATTAAAATAAAAAATTATATTATAAAGAAAGAATATATGAAATATATATATAGATTAATATTTATATTCTATATCTTTCTATTCTATA : 330
BC-i7-c3-R  : ACAAGTAGATTAAGAATTCAATATTAAAATAAAAAATTATATTATAAAGAAAGAATATATGAAATATATATATAGATTAATATTTATATTCTATATCTTTCTATTCTATA : 330
BC-i7-c4-F  : ACAAGTAGATTAAGAATTCAATATTAAAATAAAAAATTATATTATAAAGAAAGAATATATGAAATATATATATAGATTAATATTTATATTCTATATCTTTCTATTCTATA : 329
BC-i7-c4-R  : ACAAGTAGATTAAGAATTCAATATTAAAATAAAAAATTATATTATAAAGAAAGAATATATGAAATATATATATAGATTAATATTTATATTCTATATCTTTCTATTCTATA : 329
BC-i7-c5-F  : ACAAGTAGATTAAGAATTCAATATTAAAATAAAAAATTATATTATAAAGAAAGAATATATGAAATATATATATAGATTAATATTTATATTCTATATCTTTCTATTCTATA : 329
BC-i7-c5-R  : ACAAGTAGATTAAGAATTCAATATTAAAATAAAAAATTATATTATAAAGAAAGAATATATGAAATATATATATAGATTAATATTTATATTCTATATCTTTCTATTCTATA : 329
BC-i7-c6-F  : ACAAGTAGATTAAGAATTCAATATTAAAATAAAAAATTATATTATAAAGAAAGAATATATGAAATATATATATAGATTAATATTTATATTCTATATCTTTCTATTCTATA : 330
BC-i7-c6-R  : ACAAGTAGATTAAGAATTCAATATTAAAATAAAAAATTATATTATAAAGAAAGAATATATGAAATATATATATAGATTAATATTTATATTCTATATCTTTCTATTCTATA : 330
BC-i7-c7-F  : ACAAGTAGATTAAGAATTCAATATTAAAATAAAAAATTATATTATAAAGAAAGAATATATGAAATATATATATAGATTAATATTTATATTCTATATCTTTCTATTCTATA : 330
BC-i7-c7-R  : ACAAGTAGATTAAGAATTCAATATTAAAATAAAAAATTATATTATAAAGAAAGAATATATGAAATATATATATAGATTAATATTTATATTCTATATCTTTCTATTCTATA : 330
BC-i7-c8-F  : ACAAGTAGATTAAGAATTCAATATTAAAATAAAAAATTATATTATAAAGAAAGAATATATGAAATATATATATAGATTAATATTTATATTCTATATCTTTCTATTCTATA : 329
BC-i7-c8-R  : ACAAGTAGATTAAGAATTCAATATTAAAATAAAAAATTATATTATAAAGAAAGAATATATGAAATATATATATAGATTAATATTTATATTCTATATCTTTCTATTCTATA : 329
BC-i7-c9-F  : ACAAGTAGATTAAGAATTCAATATTAAAATAAAAAATTATATTATAAAGAAAGAATATATGAAATATATATATAGATTAATATTTATATTCTATATCTTTCTATTCTATA : 329
BC-i7-c9-R  : ACAAGTAGATTAAGAATTCAATATTAAAATAAAAAATTATATTATAAAGAAAGAATATATGAAATATATATATAGATTAATATTTATATTCTATATCTTTCTATTCTATA : 329
BC-i7-c10-F : ACAAGTAGATTAAGAATTCAATATTAAAATAAAAAATTATATTATAAAGAAAGAATATATGAAATATATATATAGATTAATATTTATATTCTATATCTTTCTATTCTATA : 330
BC-i7-c10-R : ACAAGTAGATTAAGAATTCAATATTAAAATAAAAAATTATATTATAAAGAAAGAATATATGAAATATATATATAGATTAATATTTATATTCTATATCTTTCTATTCTATA : 328
              ACAAGTAgATTAAGAATTCAATATTAAAATAAAAAATTATATTATAAAGAAAGAATATATGAAATATATATATAGATTAATATTTATATTCTATATCTTTCTATTCTATA

                         340         *        360         *        380         *        400         *        420         *        440
BC          : TAATAATAAAATAGAAAATAATTTTCAAAATGGATAATATAATAATTGAAATAGAAAGAATTCAAATATGGATATTCAATTCATATTTCTTATAAATTAATATTTATGAA : 439
BC-i1-c11-F : TAATAATAAAATAGAAAATAATTTTCAAAATGGATAATATAATAATTGAAATAGAAAGAATTCAAATATGGATATTCAATTCATATTTCTTATAAATTAATATTTATGAA : 439
BC-i1-c11-R : TAATAATAAAATAGAAAATAATTTTCAAAATGGATAATATAATAATTGAAATAGAAAGAATTCAAATATGGATATTCAATTCATATTTCTTATAAATTAATATTTATGAA : 439
BC-i1-c12-F : TAATAATAAAATAGAAAATAATTTTCAAAATGGATAATATAATAATTGAAATAGAAAGAATTCAAATATGGATATTCAATTCATATTTCTTATAAATTAATATTTATGAA : 438
BC-i1-c12-R : TAATAATAAAATAGAAAATAATTTTCAAAATGGATAATATAATAATTGAAATAGAAAGAATTCAAATATGGATATTCAATTCATATTTCTTATAAATTAATATTTATGAA : 438
BC-i1-c13-F : TAATAATAAAATAGAAAATAATTTTCAAAATGGATAATATAATAATTGAAATAGAAAGAATTCAAATATGGATATTCAATTCATATTTCTTATAAATTAATATTTATGAA : 439
BC-i1-c13-R : TAATAATAAAATAGAAAATAATTTTCAAAATGGATAATATAATAATTGAAATAGAAAGAATTCAAATATGGATATTCAATTCATATTTCTTATAAATTAATATTTATGAA : 439
BC-i1-c18-F : TAATAATAAAATAGAAAATAATTTTCAAAATGGATAATATAATAATTGAAATAGAAAGAATTCAAATATGGATATTCAATTCATATTTCTTATAAATTAATATTTATGAA : 439
BC-i1-c18-R : TAATAATAAAATAGAAAATAATTTTCAAAATGGATAATATAATAATTGAAATAGAAAGAATTCAAATATGGATATTCAATTCATATTTCTTATAAATTAATATTTATGAA : 439
BC-i1-c19-F : TAATAATAAAATAGAAAATAATTTTCAAAATGGATAATATAATAATTGAAATAGAAAGAATTCAAATATGGATATTCAATTCATATTTCTTATAAATTAATATTTATGAA : 439
BC-i1-c19-R : TAATAATAAAATAGAAAATAATTTTCAAAATGGATAATATAATAATTGAAATAGAAAGAATTCAAATATGGATATTCAATTCATATTTCTTATAAATTAATATTTATGAA : 439
BC-i1-c20-F : TAATAATAAAATAGAAAATAATTTTCAAAATGGATAATATAATAATTGAAATAGAAAGAATTCAAATATGGATATTCAATTCATATTTCTTATAAATTAATATTTATGAA : 438
BC-i1-c20-R : TAATAATAAAATAGAAAATAATTTTCAAAATGGATAATATAATAATTGAAATAGAAAGAATTCAAATATGGATATTCAATTCATATTTCTTATAAATTAATATTTATGAA : 438
BC-i5-c1-F  : TAATAATAAAATAGAAAATAATTTTCAAAATGGATAATATAATAATTGAAATAGAAAGAATTCAAATATGGATATTCAATTCATATTTCTTATAAATTAATATTTATGAA : 440
BC-i5-c1-R  : TAATAATAAAATAGAAAATAATTTTCAAAATGGATAATATAATAATTGAAATAGAAAGAATTCAAATATGGATATTCAATTCATATTTCTTATAAATTAATATTTATGAA : 440
BC-i5-c3-F  : TAATAATAAAATAGAAAATAATTTTCAAAATGGATAATATAATAATTGAAATAGAAAGAATTCAAATATGGATATTCAATTCATATTTCTTATAAATTAATATTTATGAA : 440
BC-i5-c3-R  : TAATAATAAAATAGAAAATAATTTTCAAAATGGATAATATAATAATTGAAATAGAAAGAATTCAAATATGGATATTCAATTCATATTTCTTATAAATTAATATTTATGAA : 440
BC-i5-c4-F  : TAATAATAAAATAGAAAATAATTTTCAAAATGGATAATATAATAATTGAAATAGAAAGAATTCAAATATGGATATTCAATTCATATTTCTTATAAATTAATATTTATGAA : 440
BC-i5-c4-R  : TAATAATAAAATAGAAAATAATTTTCAAAATGGATAATATAATAATTGAAATAGAAAGAATTCAAATATGGATATTCAATTCATATTTCTTATAAATTAATATTTATGAA : 440
BC-i5-c5-F  : TAATAATAAAATAGAAAATAATTTTCAAAATGGATAATATAATAATTGAAATAGAAAGAATTCAAATATGGATATTCAATTCATATTTCTTATAAATTAATATTTATGAA : 440
BC-i5-c5-R  : TAATAATAAAATAGAAAATAATTTTCAAAATGGATAATATAATAATTGAAATAGAAAGAATTCAAATATGGATATTCAATTCATATTTCTTATAAATTAATATTTATGAA : 440
BC-i5-c6-F  : TAATAATAAAATAGAAAATAATTTTCAAAATGGATAATATAATAATTGAAATAGAAAGAATTCAAATATGGATATTCAATTCATATTTCTTATAAATTAATATTTATGAA : 440
```

```
BC-i5-c6-R  : TAATAATAAAATAGAAAATAATTTTCAAAATGGATAATATAATAATTGAAATAGAAAGAATTCAAATATGGATATTCAATTCATATTTCTTATAAATTAATATTTATGAA : 440
BC-i5-c7-F  : TAATAATAAAATAGAAAATAATTTTCAAAATGGATAATATAATAATTGAAATAGAAAGAATTCAAATATGGATATTCAATTCATATTTCTTATAAATTAATATTTATGAA : 439
BC-i5-c7-R  : TAATAATAAAATAGAAAATAATTTTCAAAATGGATAATATAATAATTGAAATAGAAAGAATTCAAATATGGATATTCAATTCATATTTCTTATAAATTAATATTTATGAA : 439
BC-i5-c8-F  : TAATAATAAAATAGAAAATAATTTTCAAAATGGATAATATAATAATTGAAATAGAAAGAATTCAAATATGGATATTCAATTCATATTTCTTATAAATTAATATTTATGAA : 440
BC-i5-c8-R  : TAATAATAAAATAGAAAATAATTTTCAAAATGGATAATATAATAATTGAAATAGAAAGAATTCAAATATGGATATTCAATTCATATTTCTTATAAATTAATATTTATGAA : 440
BC-i5-c9-F  : TAATAATAAAATAGAAAATAATTTTCAAAATGGATAATATAATAATTGAAATAGAAAGAATTCAAATATGGATATTCAATTCATATTTCTTATAAATTAATATTTATGAA : 439
BC-i5-c9-R  : TAATAATAAAATAGAAAATAATTTTCAAAATGGATAATATAATAATTGAAATAGAAAGAATTCAAATATGGATATTCAATTCATATTTCTTATAAATTAATATTTATGAA : 439
BC-i5-c10-F : TAATAATAAAATAGAAAATAATTTTCAAAATGGATAATATAATAATTGAAATAGAAAGAATTCAAATATGGATATTCAATTCATATTTCTTATAAATTAATATTTATGAA : 440
BC-i5-c10-R : TAATAATAAAATAGAAAATAATTTTCAAAATGGATAATATAATAATTGAAATAGAAAGAATTCAAATATGGATATTCAATTCATATTTCTTATAAATTAATATTTATGAA : 440
BC-i6-c11-F : TAATAATAAAATAGAAAATAATTTTCAAAATGGATAATATAATAATTGAAATAGAAAGAATTCAAATATGGATATTCAATTCATATTTCTTATAAATTAATATTTATGAA : 440
BC-i6-c11-R : TAATAATAAAATAGAAAATAATTTTCAAAATGGATAATATAATAATTGAAATAGAAAGAATTCAAATATGGATATTCAATTCATATTTCTTATAAATTAATATTTATGAA : 440
BC-i6-c12-F : TAATAATAAAATAGAAAATAATTTTCAAAATGGATAATATAATAATTGAAATAGAAAGAATTCAAATATGGATATTCAATTCATATTTCTTATAAATTAATATTTATGAA : 439
BC-i6-c12-R : TAATAATAAAATAGAAAATAATTTTCAAAATGGATAATATAATAATTGAAATAGAAAGAATTCAAATATGGATATTCAATTCATATTTCTTATAAATTAATATTTATGAA : 439
BC-i6-c13-F : TAATAATAAAATAGAAAATAATTTTCAAAATGGATAATATAATAATTGAAATAGAAAGAATTCAAATATGGATATTCAATTCATATTTCTTATAAATTAATATTTATGAA : 440
BC-i6-c13-R : TAATAATAAAATAGAAAATAATTTTCAAAATGGATAATATAATAATTGAAATAGAAAGAATTCAAATATGGATATTCAATTCATATTTCTTATAAATTAATATTTATGAA : 440
BC-i6-c14-F : TAATAATAAAATAGAAAATAATTTTCAAAATGGATAATATAATAATTGAAATAGAAAGAATTCAAATATGGATATTCAATTCATATTTCTTATAAATTAATATTTATGAA : 439
BC-i6-c14-R : TAATAATAAAATAGAAAATAATTTTCAAAATGGATAATATAATAATTGAAATAGAAAGAATTCAAATATGGATATTCAATTCATATTTCTTATAAATTAATATTTATGAA : 439
BC-i6-c16-F : TAATAATAAAATAGAAAATAATTTTCAAAATGGATAATATAATAATTGAAATAGAAAGAATTCAAATATGGATATTCAATTCATATTTCTTATAAATTAATATTTATGAA : 440
BC-i6-c16-R : TAATAATAAAATAGAAAATAATTTTCAAAATGGATAATATAATAATTGAAATAGAAAGAATTCAAATATGGATATTCAATTCATATTTCTTATAAATTAATATTTATGAA : 440
BC-i6-c17-F : TAATAATAAAATAGAAAATAATTTTCAAAATGGATAATATAATAATTGAAATAGAAAGAATTCAAATATGGATATTCAATTCATATTTCTTATAAATTAATATTTATGAA : 439
BC-i6-c17-R : TAATAATAAAATAGAAAATAATTTTCAAAATGGATAATATAATAATTGAAATAGAAAGAATTCAAATATGGATATTCAATTCATATTTCTTATAAATTAATATTTATGAA : 439
BC-i6-c19-F : TAATAATAAAATAGAAAATAATTTTCAAAATGGATAATATAATAATTGAAATAGAAAGAATTCAAATATGGATATTCAATTCATATTTCTTATAAATTAATATTTATGAA : 439
BC-i6-c19-R : TAATAATAAAATAGAAAATAATTTTCAAAATGGATAATATAATAATTGAAATAAAAAGAATTCAAATATGGATATTCAATTCATATTTCTTATAAATTAATATTTATGAA : 439
BC-i7-c1-F  : TAATAATAAAATAGAAAATAATTTTCAAAATGGATAATATAATAATTGAAATAGAAAGAATTCAAATATGGATATTCAATTCATATTTCTTATAAATTAATATTTATGAA : 440
BC-i7-c1-R  : TAATAATAAAATAGAAAATAATTTTCAAAATGGATAATATAATAATTGAAATAGAAAGAATTCAAATATGGATATTCAATTCATATTTCTTATAAATTAATATTTATGAA : 438
BC-i7-c2-F  : TAATAATAAAATAGAAAATAATTTTCAAAATGGATAATATAATAATTGAAATAGAAAGAATTCAAATATGGATATTCAATTCATATTTCTTATAAATTAATATTTATGAA : 440
BC-i7-c2-R  : TAATAATAAAATAGAAAATAATTTTCAAAATGGATAATATAATAATTGAAATAGAAAGAATTCAAATATGGATATTCAATTCATATTTCTTATAAATTAATATTTATGAA : 440
BC-i7-c3-F  : TAATAATAAAATAGAAAATAATTTTCAAAATGGATAATATAATAATTGAAATAGAAAGAATTCAAATATGGATATTCAATTCATATTTCTTATAAATTAATATTTATGAA : 440
BC-i7-c3-R  : TAATAATAAAATAGAAAATAATTTTCAAAATGGATAATATAATAATTGAAATAGAAAGAATTCAAATATGGATATTCAATTCATATTTCTTATAAATTAATATTTATGAA : 440
BC-i7-c4-F  : TAATAATAAAATAGAAAATAATTTTCAAAATGGATAATATAATAATTGAAATAGAAAGAATTCAAATATGGATATTCAATTCATATTTCTTATAAATTAATATTTATGAA : 439
BC-i7-c4-R  : TAATAATAAAATAGAAAATAATTTTCAAAATGGATAATATAATAATTGAAATAGAAAGAATTCAAATATGGATATTCAATTCATATTTCTTATAAATTAATATTTATGAA : 439
BC-i7-c5-F  : TAATAATAAAATAGAAAATAATTTTCAAAATGGATAATATAATAATTGAAATAGAAAGAATTCAAATATGGATATTCAATTCATATTTCTTATAAATTAATATTTATGAA : 439
BC-i7-c5-R  : TAATAATAAAATAGAAAATAATTTTCAAAATGGATAATATAATAATTGAAATAGAAAGAATTCAAATATGGATATTCAATTCATATTTCTTATAAATTAATATTTATGAA : 439
BC-i7-c6-F  : TAATAATAAAATAGAAAATAATTTTCAAAATGGATAATATAATAATTGAAATAGAAAGAATTCAAATATGGATATTCAATTCATATTTCTTATAAATTAATATTTATGAA : 440
BC-i7-c6-R  : TAATAATAAAATAGAAAATAATTTTCAAAATGGATAATATAATAATTGAAATAGAAAGAATTCAAATATGGATATTCAATTCATATTTCTTATAAATTAATATTTATGAA : 440
BC-i7-c7-F  : TAATAATAAAATAGAAAATAATTTTCAAAATGGATAATATAATAATTGAAATAGAAAGAATTCAAATATGGATATTCAATTCATATTTCTTATAAATTAATATTTATGAA : 440
BC-i7-c7-R  : TAATAATAAAATAGAAAATAATTTTCAAAATGGATAATATAATAATTGAAATAGAAAGAATTCAAATATGGATATTCAATTCATATTTCTTATAAATTAATATTTATGAA : 440
BC-i7-c8-F  : TAATAATAAAATAGAAAATAATTTTCAAAATGGATAATATAATAATTGAAATAGAAAGAATTCAAATATGGATATTCAATTCATATTTCTTATAAATTAATATTTATGAA : 439
BC-i7-c8-R  : TAATAATAAAATAGAAAATAATTTTCAAAATGGATAATATAATAATTGAAATAGAAAGAATTCAAATATGGATATTCAATTCATATTTCTTATAAATTAATATTTATGAA : 439
BC-i7-c9-F  : TAATAATAAAATAGAAAATAATTTTCAAAATGGATAATATAATAATTGAAATAGAAAGAATTCAAATATGGATATTCAATTCATATTTCTTATAAATTAATATTTATGAA : 439
BC-i7-c9-R  : TAATAATAAAATAGAAAATAATTTTCAAAATGGATAATATAATAATTGAAATAGAAAGAATTCAAATATGGATATTCAATTCATATTTCTTATAAATTAATATTTATGAA : 439
BC-i7-c10-F : TAATAATAAAATAGAAAATAATTTTCAAAATGGATAATATAATAATTGAAATAGAAAGAATTCAAATATGGATATTCAATTCATATTTCTTATAAATTAATATTTATGAA : 440
BC-i7-c10-R : TAATAATAAAATAGAAAATAATTTTCAAAATGGATAATATAATAATTGAAATAGAAAGAATTCAAATATGGATATTCAATTCATATTTCTTATAAATTAATATTTATGAA : 438
              TAATAATAAAATAGAAAATAATTTTCAAAATGGATAATATAATAATTGAAATAgAAAGAATTCAAATATGGATATTCAATTCATATTTCTTATAAATTAATATTTATGAA

                    *       460         *       480         *       500         *       520         *       540         *
```

```
BC          : TATTCATAAATGAATAACTGAGTAATAAAACTAAAAAAAAATGAATAAAATAAGATCAGATTATTCTATAGAATAACCTTAGACTTGGAATTGACGAACAACCAATAATAG : 549
BC-i1-c11-F : TATTCATAAATGAATAACTGAGTAATAAAACTAAAAAAAAATGAATAAAATAAGATCAGATTATTCTATAGAATAACCTTAGACTTGGAATTGACGAACAACCAATAATAG : 549
BC-i1-c11-R : TATTCATAAATGAATAACTGAGTAATAAAACTAAAAAAAAATGAATAAAATAAGATCAGATTATTCTATAGAATAACCTTAGACTTGGAATTGACGAACAACCAATAATAG : 549
BC-i1-c12-F : TATTCATAAATGAATAACTGAGTAATAAAACTAAAAAAAAATGAATAAAATAAGATCAGATTATTCTATAGAATAACCTTAGACTTGGAATTGACGAACAACCAATAATAG : 548
BC-i1-c12-R : TATTCATAAATGAATAACTGAGTAATAAAACTAAAAAAAAATGAATAAAATAAGATCAGATTATTCTATAGAATAACCTTAGACTTGGAATTGACGAACAACCAATAATAG : 548
BC-i1-c13-F : TATTCATAAATGAATAACTGAGTAATAAAACTAAAAAAAAATGAATAAAATAAGATCAGATTATTCTATAGAATAACCTTAGACTTGGAATTGACGAACAACCAATAATAG : 549
BC-i1-c13-R : TATTCATAAATGAATAACTGAGTAATAAAACTAAAAAAAAATGAATAAAATAAGATCAGATTATTCTATAGAATAACCTTAGACTTGGAATTGACGAACAACCAATAATAG : 549
BC-i1-c18-F : TATTCATAAATGAATAACTGAGTAATAAAACTAAAAAAAAATGAATAAAATAAGATCAGATTATTCTATAGAATAACCTTAGACTTGGAATTGACGAACAACCAATAATAG : 549
BC-i1-c18-R : TATTCATAAATGAATAACTGAGTAATAAAACTAAAAAAAAATGAATAAAATAAGATCAGATTATTCTATAGAATAACCTTAGACTTGGAATTGACGAACAACCAATAATAG : 549
BC-i1-c19-F : TATTCATAAATGAATAACTGAGTAATAAAACTAAAAAAAAATGAATAAAATAAGATCAGATTATTCTATAGAATAACCTTAGACTTGGAATTGACGAACAACCAATAATAG : 549
BC-i1-c19-R : TATTCATAAATGAATAACTGAGTAATAAAACTAAAAAAAAATGAATAAAATAAGATCAGATTATTCTATAGAATAACCTTAGACTTGGAATTGACGAACAACCAATAATAG : 549
BC-i1-c20-F : TATTCATAAATGAATAACTGAGTAATAAAACTAAAAAAAAATGAATAAAATAAGATCAGATTATTCTATAGAATAACCTTAGACTTGGAATTGACGAACAACCAATAATAG : 548
BC-i1-c20-R : TATTCATAAATGAATAACTGAGTAATAAAACTAAAAAAAAATGAATAAAATAAGATCAGATTATTCTATAGAATAACCTTAGACTTGGAATTGACGAACAACCAATAATAG : 548
BC-i5-c1-F  : TATTCATAAATGAATAACTGAGTAATAAAACTAAAAAAAAATGAATAAAATAAGATCAGATTATTCTATAGAATAACCTTAGACTTGGAATTGACGAACAACCAATAATAG : 550
BC-i5-c1-R  : TATTCATAAATGAATAACTGAGTAATAAAACTAAAAAAAAATGAATAAAATAAGATCAGATTATTCTATAGAATAACCTTAGACTTGGAATTGACGAACAACCAATAATAG : 550
BC-i5-c3-F  : TATTCATAAATGAATAACTGAGTAATAAAACTAAAAAAAAATGAATAAAATAAGATCAGATTATTCTATAGAATAACCTTAGACTTGGAATTGACGAACAACCAATAATAG : 550
BC-i5-c3-R  : TATTCATAAATGAATAACTGAGTAATAAAACTAAAAAAAAATGAATAAAATAAGATCAGATTATTCTATAGAATAACCTTAGACTTGGAATTGACGAACAACCAATAATAG : 550
BC-i5-c4-F  : TATTCATAAATGAATAACTGAGTAATAAAACTAAAAAAAAATGAATAAAATAAGATCAGATTATTCTATAGAATAACCTTAGACTTGGAATTGACGAACAACCAATAATAG : 550
BC-i5-c4-R  : TATTCATAAATGAATAACTGAGTAATAAAACTAAAAAAAAATGAATAAAATAAGATCAGATTATTCTATAGAATAACCTTAGACTTGGAATTGACGAACAACCAATAATAG : 550
BC-i5-c5-F  : TATTCATAAATGAATAACTGAGTAATAAAACTAAAAAAAAATGAATAAAATAAGATCAGATTATTCTATAGAATAACCTTAGACTTGGAATTGACGAACAACCAATAATAG : 550
BC-i5-c5-R  : TATTCATAAATGAATAACTGAGTAATAAAACTAAAAAAAAATGAATAAAATAAGATCAGATTATTCTATAGAATAACCTTAGACTTGGAATTGACGAACAACCAATAATAG : 550
BC-i5-c6-F  : TATTCATAAATGAATAACTGAGTAATAAAACTAAAAAAAAATGAATAAAATAAGATCAGATTATTCTATAGAATAACCTTAGACTTGGAATTGACGAACAACCAATAATAG : 550
BC-i5-c6-R  : TATTCATAAATGAATAACTGAGTAATAAAACTAAAAAAAAATGAATAAAATAAGATCAGATTATTCTATAGAATAACCTTAGACTTGGAATTGACGAACAACCAATAATAG : 550
BC-i5-c7-F  : TATTCATAAATGAATAACTGAGTAATAAAACTAAAAAAAAATGAATAAAATAAGATCAGATTATTCTATAGAATAACCTTAGACTTGGAATTGACGAACAACCAATAATAG : 549
BC-i5-c7-R  : TATTCATAAATGAATAACTGAGTAATAAAACTAAAAAAAAATGAATAAAATAAGATCAGATTATTCTATAGAATAACCTTAGACTTGGAATTGACGAACAACCAATAATAG : 549
BC-i5-c8-F  : TATTCATAAATGAATAACTGAGTAATAAAACTAAAAAAAAATGAATAAAATAAGATCAGATTATTCTATAGAATAACCTTAGACTTGGAATTGACGAACAACCAATAATAG : 550
BC-i5-c8-R  : TATTCATAAATGAATAACTGAGTAATAAAACTAAAAAAAAATGAATAAAATAAGATCAGATTATTCTATAGAATAACCTTAGACTTGGAATTGACGAACAACCAATAATAG : 550
BC-i5-c9-F  : TATTCATAAATGAATAACTGAGTAATAAAACTAAAAAAAAATGAATAAAATAAGATCAGATTATTCTATAGAATAACCTTAGACTTGGAATTGACGAACAACCAATAATAG : 549
BC-i5-c9-R  : TATTCATAAATGAATAACTGAGTAATAAAACTAAAAAAAAATGAATAAAATAAGATCAGATTATTCTATAGAATAACCTTAGACTTGGAATTGACGAACAACCAATAATAG : 549
BC-i5-c10-F : TATTCATAAATGAATAACTGAGTAATAAAACTAAAAAAAAATGAATAAAATAAGATCAGATTATTCTATAGAATAACCTTAGACTTGGAATTGACGAACAACCAATAATAG : 550
BC-i5-c10-R : TATTCATAAATGAATAACTGAGTAATAAAACTAAAAAAAAATGAATAAAATAAGATCAGATTATTCTATAGAATAACCTTAGACTTGGAATTGACGAACAACCAATAATAG : 550
BC-i6-c11-F : TATTCATAAATGAATAACTGAGTAATAAAACTAAAAAAAAATGAATAAAATAAGATCAGATTATTCTATAGAATAACCTTAGACTTGGAATTGACGAACAACCAATAATAG : 550
BC-i6-c11-R : TATTCATAAATGAATAACTGAGTAATAAAACTAAAAAAAAATGAATAAAATAAGATCAGATTATTCTATAGAATAACCTTAGACTTGGAATTGACGAACAACCAATAATAG : 550
BC-i6-c12-F : TATTCATAAATGAATAACTGAGTAATAAAACTAAAAAAAAATGAATAAAATAAGATCAGATTATTCTATAGAATAACCTTAGACTTGGAATTGACGAACAACCAATAATAG : 549
BC-i6-c12-R : TATTCATAAATGAATAACTGAGTAATAAAACTAAAAAAAAATGAATAAAATAAGATCAGATTATTCTATAGAATAACCTTAGACTTGGAATTGACGAACAACCAATAATAG : 549
BC-i6-c13-F : TATTCATAAATGAATAACTGAGTAATAAAACTAAAAAAAAATGAATAAAATAAGATCAGATTATTCTATAGAATAACCTTAGACTTGGAATTGACGAACAACCAATAATAG : 550
BC-i6-c13-R : TATTCATAAATGAATAACTGAGTAATAAAACTAAAAAAAAATGAATAAAATAAGATCAGATTATTCTATAGAATAACCTTAGACTTGGAATTGACGAACAACCAATAATAG : 550
BC-i6-c14-F : TATTCATAAATGAATAACTGAGTAATAAAACTAAAAAAAAATGAATAAAATAAGATCAGATTATTCTATAGAATAACCTTAGACTTGGAATTGACGAACAACCAATAATAG : 549
BC-i6-c14-R : TATTCATAAATGAATAACTGAGTAATAAAACTAAAAAAAAATGAATAAAATAAGATCAGATTATTCTATAGAATAACCTTAGACTTGGAATTGACGAACAACCAATAATAG : 549
BC-i6-c16-F : TATTCATAAATGAATAACTGAGTAATAAAACTAAAAAAAAATGAATAAAATAAGATCAGATTATTCTATAGAATAACCTTAGACTTGGAATTGACGAACAACCAATAATAG : 550
BC-i6-c16-R : TATTCATAAATGAATAACTGAGTAATAAAACTAAAAAAAAATGAATAAAATAAGATCAGATTATTCTATAGAATAACCTTAGACTTGGAATTGACGAACAACCAATAATAG : 550
BC-i6-c17-F : TATTCATAAATGAATAACTGAGTAATAAAACTAAAAAAAAATGAATAAAATAAGATCAGATTATTCTATAGAATAACCTTAGACTTGGAATTGACGAACAACCAATAATAG : 549
BC-i6-c17-R : TATTCATAAATGAATAACTGAGTAATAAAACTAAAAAAAAATGAATAAAATAAGATCAGATTATTCTATAGAATAACCTTAGACTTGGAATTGACGAACAACCAATAATAG : 549
BC-i6-c19-F : TATTCATAAATGAATAACTGAGTAATAAAACTAAAAAAAAATGAATAAAATAAGATCAGATTATTCTATAGAATAACCTTAGACTTGGAATTGACGAACAACCAATAATAG : 549
BC-i6-c19-R : TATTCATAAATGAATAACTGAGTAATAAAACTAAAAAAAAATGAATAAAATAAGATCAGATTATTCTATAGAATAACCTTAAACTTGGAATTGACGAACAACCAATAATAG : 549
BC-i7-c1-F  : TATTCATAAATGAATAACTGAGTAATAAAACTAAAAAAAAATGAATAAAATAAGATCAGATTATTCTATAGAATAACCTTAGACTTGGAATTGACGAACAACCAATAATAG : 550
```

```
BC-i7-c1-R  : TATTCATAAATGAATAACTGAGTAATAAAACTAAAAAAAAATGAATAAAATAAGATCAGATTATTCTATAGAATAACCTTAGACTTGGAATTGACGAACAACCAATAATAG : 548
BC-i7-c2-F  : TATTCATAAATGAATAACTGAGTAATAAAACTAAAAAAAAATGAATAAAATAAGATCAGATTATTCTATAGAATAACCTTAGACTTGGAATTGACGAACAACCAATAATAG : 550
BC-i7-c2-R  : TATTCATAAATGAATAACTGAGTAATAAAACTAAAAAAAAATGAATAAAATAAGATCAGATTATTCTATAGAATAACCTTAGACTTGGAATTGACGAACAACCAATAATAG : 550
BC-i7-c3-F  : TATTCATAAATGAATAACTGAGTAATAAAACTAAAAAAAAATGAATAAAATAAGATCAGATTATTCTATAGAATAACCTTAGACTTGGAATTGACGAACAACCAATAATAG : 550
BC-i7-c3-R  : TATTCATAAATGAATAACTGAGTAATAAAACTAAAAAAAAATGAATAAAATAAGATCAGATTATTCTATAGAATAACCTTAGACTTGGAATTGACGAACAACCAATAATAG : 550
BC-i7-c4-F  : TATTCATAAATGAATAACTGAGTAATAAAACTAAAAAAAAATGAATAAAATAAGATCAGATTATTCTATAGAATAACCTTAGACTTGGAATTGACGAACAACCAATAATAG : 549
BC-i7-c4-R  : TATTCATAAATGAATAACTGAGTAATAAAACTAAAAAAAAATGAATAAAATAAGATCAGATTATTCTATAGAATAACCTTAGACTTGGAATTGACGAACAACCAATAATAG : 549
BC-i7-c5-F  : TATTCATAAATGAATAACTGAGTAATAAAACTAAAAAAAAATGAATAAAATAAGATCAGATTATTCTATAGAATAACCTTAGACTTGGAATTGACGAACAACCAATAATAG : 549
BC-i7-c5-R  : TATTCATAAATGAATAACTGAGTAATAAAACTAAAAAAAAATGAATAAAATAAGATCAGATTATTCTATAGAATAACCTTAGACTTGGAATTGACGAACAACCAATAATAG : 549
BC-i7-c6-F  : TATTCATAAATGAATAACTGAGTAATAAAACTAAAAAAAAATGAATAAAATAAGATCAGATTATTCTATAGAATAACCTTAGACTTGGAATTGACGAACAACCAATAATAG : 550
BC-i7-c6-R  : TATTCATAAATGAATAACTGAGTAATAAAACTAAAAAAAAATGAATAAAATAAGATCAGATTATTCTATAGAATAACCTTAGACTTGGAATTGACGAACAACCAATAATAG : 550
BC-i7-c7-F  : TATTCATAAATGAATAACTGAGTAATAAAACTAAAAAAAAATGAATAAAATAAGATCAGATTATTCTATAGAATAACCTTAGACTTGGAATTGACGAACAACCAATAATAG : 550
BC-i7-c7-R  : TATTCATAAATGAATAACTGAGTAATAAAACTAAAAAAAAATGAATAAAATAAGATCAGATTATTCTATAGAATAACCTTAGACTTGGAATTGACGAACAACCAATAATAG : 550
BC-i7-c8-F  : TATTCATAAATGAATAACTGAGTAATAAAACTAAAAAAAAATGAATAAAATAAGATCAGATTATTCTATAGAATAACCTTAGACTTGGAATTGACGAACAACCAATAATAG : 549
BC-i7-c8-R  : TATTCATAAATGAATAACTGAGTAATAAAACTAAAAAAAAATGAATAAAATAAGATCAGATTATTCTATAGAATAACCTTAGACTTGGAATTGACGAACAACCAATAATAG : 549
BC-i7-c9-F  : TATTCATAAATGAATAACTGAGTAATAAAACTAAAAAAAAATGAATAAAATAAGATCAGATTATTCTATAGAATAACCTTAGACTTGGAATTGACGAACAACCAATAATAG : 549
BC-i7-c9-R  : TATTCATAAATGAATAACTGAGTAATAAAACTAAAAAAAAATGAATAAAATAAGATCAGATTATTCTATAGAATAACCTTAGACTTGGAATTGACGAACAACCAATAATAG : 549
BC-i7-c10-F : TATTCATAAATGAATAACTGAGTAATAAAACTAAAAAAAAATGAATAAAATAAGATCAGATTATTCTATAGAATAACCTTAGACTTGGAATTGACGAACAACCAATAATAG : 550
BC-i7-c10-R : TATTCATAAATGAATAACTGAGTAATAAAACTAAAAAAAAATGAATAAAATAAGATCAGATTATTCTATAGAATAACCTTAGACTTGGAATTGACGAACAACCAATAATAG : 548
              TATTCATAAATGAATAACTGAGTAATAAAACTAAAAAAAAATGAATAAAATAAGATCAGATTATTCTATAGAATAACCTTAgACTTGGAATTGACGAACAACCAATAATAG

                    560         *        580         *        600
BC          : TATTTATTAGTGTCGAATCGAAAATGGGGCGTGGCCAAGCGGTAAGGCAACGGGTTT- : 606
BC-i1-c11-F : TATTTATTAGTGTCGAATCGAAAATGGGGCGTGGCCAAGCGGTAAGGCAACGGGTTT- : 606
BC-i1-c11-R : TATTTATTAGTGTCGAATCGAAAATGGGGCGTGGCCAAGCGGTAAGGCAACGGGTTT- : 606
BC-i1-c12-F : TATTTATTAGTGTCGAATCGAAAATGGGGCGTGGCCAAGCGGTAAGGCAACGGGTT-- : 604
BC-i1-c12-R : TATTTATTAGTGTCGAATCGAAAATGGGGCGTGGCCAAGCGGTAAGGCAACGGGTT-- : 604
BC-i1-c13-F : TATTTATTAGTGTCGAATCGAAAATGGGGCGTGGCCAAGCGGTAAGGCAACGGGTTT- : 606
BC-i1-c13-R : TATTTATTAGTGTCGAATCGAAAATGGGGCGTGGCCAAGCGGTAAGGCAACGGGTTT- : 606
BC-i1-c18-F : TATTTATTAGTGTCGAATCGAAAATGGGGCGTGGCCAAGCGGTAAGGCAACGGGTTT- : 606
BC-i1-c18-R : TATTTATTAGTGTCGAATCGAAAATGGGGCGTGGCCAAGCGGTAAGGCAACGGGTTT- : 606
BC-i1-c19-F : TATTTATTAGTGTCGAATCGAAAATGGGGCGTGGCCAAGCGGTAAGGCAACGGGTTT- : 606
BC-i1-c19-R : TATTTATTAGTGTCGAATCGAAAATGGGGCGTGGCCAAGCGGTAAGGCAACGGGTTT- : 606
BC-i1-c20-F : TATTTATTAGTGTCGAATCGAAAATGGGGCGTGGCCAAGCGGTAAGGCAACGGGTTT- : 605
BC-i1-c20-R : TATTTATTAGTGTCGAATCGAAAATGGGGCGTGGCCAAGCGGTAAGGCAACGGGTTT- : 605
BC-i5-c1-F  : TATTTATTAGTGTCGAATCGAAAATGGGGCGTGGCCAAGCGGTAAGGCAACGG----- : 603
BC-i5-c1-R  : TATTTATTAGTGTCGAATCGAAAATGGGGCGTGGCCAAGCGGTAAGGCAACGGGTTT- : 607
BC-i5-c3-F  : TATTTATTAGTGTCGAATCGAAAATGGGGCGTGGCCAAGCGGTAAGGCAACGG----- : 603
BC-i5-c3-R  : TATTTATTAGTGTCGAATCGAAAATGGGGCGTGGCCAAGCGGTAAGGCAACGGGTTT- : 607
BC-i5-c4-F  : TATTTATTAGTGTCGAATCGAAAATGGGGCGTGGCCAAGCGGTAAGGCAACGG----- : 603
BC-i5-c4-R  : TATTTATTAGTGTCGAATCGAAAATGGGGCGTGGCCAAGCGGTAAGGCAACGGGTTT- : 607
BC-i5-c5-F  : TATTTATTAGTGTCGAATCGAAAATGGGGCGTGGCCAAGCGGTAAGGCAACGG----- : 603
BC-i5-c5-R  : TATTTATTAGTGTCGAATCGAAAATGGGGCGTGGCCAAGCGGTAAGGCAACGGGTTT- : 607
BC-i5-c6-F  : TATTTATTAGTGTCGAATCGAAAATGGGGCGTGGCCAAGCGGTAAGGCAACGG----- : 603
BC-i5-c6-R  : TATTTATTAGTGTCGAATCGAAAATGGGGCGTGGCCAAGCGGTAAGGCAACGGGTTT- : 607
BC-i5-c7-F  : TATTTATTAGTGTCGAATCGAAAATGGGGCGTGGCCAAGCGGTAAGGCAACGG----- : 602
```

```
BC-i5-c7-R  : TATTTATTAGTGTCGAATCGAAAATGGGGCGTGGCCAAGCGGTAAGGCAACGGGTTT- : 606
BC-i5-c8-F  : TATTTATTAGTGTCGAATCGAAAATGGGGCGTGGCCAAGCGGTAAGGCAACGG----- : 603
BC-i5-c8-R  : TATTTATTAGTGTCGAATCGAAAATGGGGCGTGGCCAAGCGGTAAGGCAACGGGTTT- : 607
BC-i5-c9-F  : TATTTATTAGTGTCGAATCGAAAATGGGGCGTGGCCAAGCGGTAAGGCAACGG----- : 602
BC-i5-c9-R  : TATTTATTAGTGTCGAATCGAAAATGGGGCGTGGCCAAGCGGTAAGGCAACGGGTTT- : 606
BC-i5-c10-F : TATTTATTAGTGTCGAATCGAAAATGGGGCGTGGCCAAGCGGTAAGGCAACGG----- : 603
BC-i5-c10-R : TATTTATTAGTGTCGAATCGAAAATGGGGCGTGGCCAAGCGGTAAGGCAACGGTTT-- : 606
BC-i6-c11-F : TATTTATTAGTGTCGAATCGAAAATGGGGCGTGGCCAAGCGGTAAGGCAACGGGTTT- : 607
BC-i6-c11-R : TATTTATTAGTGTCGAATCGAAAATGGGGCGTGGCCAAGCGGTAAGGCAACGGGTTT- : 607
BC-i6-c12-F : TATTTATTAGTGTCGAATCGAAAATGGGGCGTGGCCAAGCGGTAAGGCAACGGGTTT- : 606
BC-i6-c12-R : TATTTATTAGTGTCGAATCGAAAATGGGGCGTGGCCAAGCGGTAAGGCAACGGGTTT- : 606
BC-i6-c13-F : TATTTATTAGTGTCGAATCGAAAATGGGGCGTGGCCAAGCGGTAAGGCAACGGGTTT- : 607
BC-i6-c13-R : TATTTATTAGTGTCGAATCGAAAATGGGGCGTGGCCAAGCGGTAAGGCAACGGGTTT- : 607
BC-i6-c14-F : TATTTATTAGTGTCGAATCGAAAATGGGGCGTGGCCAAGCGGTAAGGCAACGGGTTT- : 606
BC-i6-c14-R : TATTTATTAGTGTCGAATCGAAAATGGGGCGTGGCCAAGCGGTAAGGCAACGGGTTT- : 606
BC-i6-c16-F : TATTTATTAGTGTCGAATCGAAAATGGGGCGTGGCCAAGCGGTAAGGCAACGGGTTT- : 607
BC-i6-c16-R : TATTTATTAGTGTCGAATCGAAAATGGGGCGTGGCCAAGCGGTAAGGCAACGGGTTT- : 607
BC-i6-c17-F : TATTTATTAGTGTCGAATCGAAAATGGGGCGTGGCCAAGCGGTAAGGCAACGGGTT-- : 605
BC-i6-c17-R : TATTTATTAGTGTCGAATCGAAAATGGGGCGTGGCCAAGCGGTAAGGCAACGGGTT-- : 605
BC-i6-c19-F : TATTTATTAGTGTCGAATCGAAAATGGGGCGTGGCCAAGCGGTAAGGCAACGGGTTT- : 606
BC-i6-c19-R : TATTTATTAGTGTCGAATCCAAAATGGGGCGTGGCCAAGCGGTAAGGCAACGGGTTT- : 606
BC-i7-c1-F  : TATTTATTAGTGTCGAATCGAAAATGGGGCGTGGCCAAGCGGTAAGGCAACGGGTTT- : 607
BC-i7-c1-R  : TATTTATTATTGTCGAATCGAAAATGGGGCGTGGCCGAGCGGTAAGGCAACGGGTTT- : 605
BC-i7-c2-F  : TATTTATTAGTGTCGAATCGAAAATGGGGCGTGGCCAAGCGGTAAGGCAACGGGTT-- : 606
BC-i7-c2-R  : TATTTATTAGTGTCGAATCGAAAATGGGGCGTGGCCAAGCGGTAAGGCAACGGGTTAT : 608
BC-i7-c3-F  : TATTTATTAGTGTCGAATCGAAAATGGGGCGTGGCCAAGCGGTAAGGCAACGGGTTT- : 607
BC-i7-c3-R  : TATTTATTAGTGTCGAATCGAAAATGGGGCGTGGCCAAGCGGTAAGGCAACGGGTTT- : 607
BC-i7-c4-F  : TATTTATTAGTGTCGAATCGAAAATGGGGCGTGGCCAAGCGGTAAGGCAACGGGTTT- : 606
BC-i7-c4-R  : TATTTATTAGTGTCGAATCGAAAATGGGGCGTGGCCAAGCGGTAAGGCAACGGGTTT- : 606
BC-i7-c5-F  : TATTTATTAGTGTCGAATCGAAAATGGGGCGTGGCCAAGCGGTAAGGCAACGGGTTT- : 606
BC-i7-c5-R  : TATTTATTAGTGTCGAATCGAAAATGGGGCGTGGCCAAGCGGTAAGGCAACGGGTTT- : 606
BC-i7-c6-F  : TATTTATTAGTGTCGAATCGAAAATGGGGCGTGGCCAAGCGGTAAGGCAACGGGTTT- : 607
BC-i7-c6-R  : TATTTATTAGTGTCGAATCGAAAATGGGGCGTGGCCAAGCGGTAAGGCAACGGGTTT- : 607
BC-i7-c7-F  : TATTTATTAGTGTCGAATCGAAAATGGGGCGTGGCCAAGCGGTAAGGCAACGGGTTT- : 607
BC-i7-c7-R  : TATTTATTAGTGTCGAATCGAAAATGGGGCGTGGCCAAGCGGTAAGGCAACGGGTTT- : 607
BC-i7-c8-F  : TATTTATTAGTGTCGAATCGAAAATGGGGCGTGGCCAAGCGGTAAGGCAACGGGTTT- : 606
BC-i7-c8-R  : TATTTATTAGTGTCGAATCGAAAATGGGGCGTGGCCAAGCGGTAAGGCAACGGGTTAT : 607
BC-i7-c9-F  : TATTTATTAGTGTCGAATCGAAAATGGGGCGTGGCCAAGCGGTAAGGCAACGGGTTT- : 606
BC-i7-c9-R  : TATTTATTAGTGTCGAATCGAAAATGGGGCGTGGCCAAGCGGTAAGGCAACGGGTTT- : 606
BC-i7-c10-F : TATTTATTAGTGTCGAATCGAAAATGGGGCGTGGCCAAGCGGTAAGGCAACGGGTTT- : 607
BC-i7-c10-R : TATTTATTAGTGTCGAATCGAAAATGGGGCGTGGCCAAGCGGTAAGGCAACGGGTTT- : 605
              TATTTATTAgTGTCGAATCgAAAATGGGGCGTGGCCaAGCGGTAAGGCAACGGgtt
```

(C) Region DE

```
                   *         20          *         40          *         60          *         80          *        100          *
DE           : ACGCAGAGATAAAGAAATAGAGAAATTGGATCGCTTGTGTGTTACCCTTACAGATGAAAAATAATCTTT-CAGATAGAAGATATATTTTGAAAAAAAAAAAATTATATTT : 109
DE-i1-c11-F  : ACGCAGAGATAAAGAAATAGAGAAATTGGATCGCTTGTGTGTTACCCTTACAGATGAAAAATAATCTTT-CAGATAGAAGATATATTTTGAAAAAAAAAAAATTATATTT : 109
DE-i1-c11-R  : ACGCAGAGATAAAGAAATAGAGAAATTGGATCGCTTGTGTGTTACCCTTACAGATGAAAAATAATCTTT-CAGATAGAAGATATATTTTGAAAAAAAAAAAATTATATTT : 109
DE-i1-c13-F  : ACGCAGAGATAAAGAAATAGAGAAATTGGATCGCTTGTGTGTTACCCTTACAGATGAAAAATAATCTTT-CAGATAGAAGATATATTTTGAAAAAAAAAAAATTATATTT : 109
DE-i1-c13-R  : ACGCAGAGATAAAGAAATAGAGAAATTGGATCGCTTGTGTGTTACCCTTACAGATGAAAAATAATCTTT-CAGATAGAAGATATATTTTGAAAAAAAAAAAATTATATTT : 109
DE-i1-c14-F  : ACGCAGAGATAAAGAAATAGAGAAATTGGATCGCTTGTGTGTTACCCTTACAGATGAAAAATAATCTTT-CAGATAGAAGATATATTTTGAAAAAAAAAAAATTATATTT : 109
DE-i1-c14-R  : ACGCAGAGATAAAGAAATAGAGAAATTGGATCGCTTGTGTGTTACCCTTACAGATGAAAAATAATCTTT-CAGATAGAAGATATATTTTGAAAAAAAAAAAATTATATTT : 109
DE-i1-c15-F  : ACGCAGAGATAAAGAAATAGAGAAATTGGATCGCTTGTGTGTTACCCTTACAGATGAAAAATAATCTTT-CAGATAGAAGATATATTTTGAAAAAAAAAAAATTATATTT : 109
DE-i1-c15-R  : ACGCAGAGATAAAGAAATAGAGAAATTGGATCGCTTGTGTGTTACCCTTACAGATGAAAAATAATCTTT-CAGATAGAAGATATATTTTGAAAAAAAAAAAATTATATTT : 109
DE-i1-c16-F  : ACGCAGAGATAAAGAAATAGAGAAATTGGATCGCTTGTGTGTTACCCTTACAGATGAAAAATAATCTTT-CAGATAGAAGATATATTTTGAAAAAAAAAAAATTATATTT : 109
DE-i1-c16-R  : ACGCAGAGATAAAGAAATAGAGAAATTGGATCGCTTGTGTGTTACCCTTACAGATGAAAAATAATCTTT-CAGATAGAAGATATATTTTGAAAAAAAAAAAATTATATTT : 109
DE-i1-c17-F  : ACGCAGAGATAAAGAAATAGAGAAATTGGATCGCTTGTGTGTTACCCTTACAGATGAAAAATAATCTTT-CAGATAGAAGATATATTTTGAAAAAAAAAAAATTATATTT : 109
DE-i1-c17-R  : ACGCAGAGATAAAGAAATAGAGAAATTGGATCGCTTGTGTGTTACCCTTACAGATGAAAAATAATCTTT-CAGATAGAAGATATATTTTGAAAAAAAAAAAATTATATTT : 109
DE-i1-c18-F  : ACGCAGAGATAAAGAAATAGAGAAATTGGATCGCTTGTGTGTTACCCTTACAGATGAAAAATAATCTTT-CAGATAGAAGATATATTTTGAAAAAAAAAAAATTATATTT : 109
DE-i1-c18-R  : ACGCAGAGATAAAGAAATAGAGAAATTGGATCGCTTGTGTGTTACCCTTACAGATGAAAAATAATCTTT-CAGATAGAAGATATATTTTGAAAAAAAAAAAATTATATTT : 109
DE-i5-c11-F  : ACGCAGAGATAAAGAAATAGAGAAATTGGATCGCTTGTGTGTTACCCTTACAGATGAAAAATAATCTTT-CAGATAGAAGATATATTTTGAAAAAAAAAAAATTATATTT : 109
DE-i5-c11-R  : ACGCAGAGATAAAGAAATAGAGAAATTGGATCGCTTGTGTGTTACCCTTACAGATGAAAAATAATCTTT-CAGATAGAAGATATATTTTGAAAAAAAAAAAATTATATTT : 109
DE-i5-c12-F  : ACGCAGAGATAAAGAAATAGAGAAATTGGATCGCTTGTGTGTTACCCTTACAGATGAAAAATAATCTTT-CAGATAGAAGATATATTTTGAAAAAAAAAAAATTATATTT : 109
DE-i5-c12-R  : ACGCAGAGATAAAGAAATAGAGAAATTGGATCGCTTGTGTGTTACCCTTACAGATGAAAAATAATCTTT-CAGATAGAAGATATATTTTGAAAAAAAAAAAATTATATTT : 109
DE-i5-c13-F  : ACGCAGAGATAAAGAAATAGAGAAATTGGATCGCTTGTGTGTTACCCTTACAGATGAAAAATAATCTTT-CAGATAGAAGATATATTTTGAAAAAAAAAAAATTATATTT : 109
DE-i5-c13-R  : ACGCAGAGATAAAGAAATAGAGAAATTGGATCGCTTGTGTGTTACCCTTACAGATGAAAAATAATCTTT-CAGATAGAAGATATATTTTGAAAAAAAAAAAATTATATTT : 109
DE-i5-c14-F  : ACGCAGAGATAAAGAAATAGAGAAATTGGATCGCTTGTGTGTTACCCTTACAGATGAAAAATAATCTTT-CAGATAGAAGATATATTTTGAAAAAAAAAAAATTATATTT : 109
DE-i5-c14-R  : ACGCAGAGATAAAGAAATAGAGAAATTGGATCGCTTGTGTGTTACCCTTACAGATGAAAAATAATCTTT-CAGATAGAAGATATATTTTGAAAAAAAAAAAATTATATTT : 109
DE-i5-c15-F  : ACGCAGAGATAAAGAAATAGAGAAATTGGATCGCTTGTGTGTTACCCTTACAGATGAAAAATAATCTTT-CAGATAGAAGATATATTTTGAAAAAAAAAAAATTATATTT : 109
DE-i5-c15-R  : ACGCAGAGATAAAGAAATAGAGAAATTGGATCGCTTGTGTGTTACCCTTACAGATGAAAAATAATCTTT-CAGATAGAAGATATATTTTGAAAAAAAAAAAATTATATTT : 109
DE-i5-c16-F  : ACGCAGAGATAAAGAAATAGAGAAATTGGATCGCTTGTGTGTTACCCTTACAGATGAAAAATAATCTTT-CAGATAGAAGATATATTTTGAAAAAAAAAAAATTATATTT : 109
DE-i5-c16-R  : ACGCAGAGATAAAGAAATAGAGAAATTGGATCGCTTGTGTGTTACCCTTACAGATGAAAAATAATCTTT-CAGATAGAAGATATATTTTGAAAAAAAAAAAATTATATTT : 109
DE-i5-c17-F  : ACGCAGAGATAAAGAAATAGAGAAATTGGATCGCTTGTGTGTTACCCTTACAGATGAAAAATAATCTTT-CAGATAGAAGATATATTTTGAAAAAAAAAAAATTATATTT : 109
DE-i5-c17-R  : ACGCAGAGATAAAGAAATAGAGAAATTGGATCGCTTGTGTGTTACCCTTACAGATGAAAAATAATCTTT-CAGATAGAAGATATATTTTGAAAAAAAAAAAATTATATTT : 109
DE-i5-c18-F  : ACGCAGAGATAAAGAAATAGAGAAATTGGATCGCTTGTGTGTTACCCTTACAGATGAAAAATAATCTTT-CAGATAGAAGATATATTTTGAAAAAAAAAAAATTATATTT : 109
DE-i5-c18-R  : ACGCAGAGATAAAGAAATAGAGAAATTGGATCGCTTGTGTGTTACCCTTACAGATGAAAAATAATCTTT-CAGATAGAAGATATATTTTGAAAAAAAAAAAATTATATTT : 109
DE-i5-c19-F  : ACGCAGAGATAAAGAAATAGAGAAATTGGATCGCTTGTGTGTTACCCTTACAGATGAAAAATAATCTTT-CAGATAGAAGATATATTTTGAAAAAAAAAAAATTATATTT : 109
DE-i5-c19-R  : ACGCAGAGATAAAGAAATAGAGAAATTGGATCGCTTGTGTGTTACCCTTACAGATGAAAAATAATCTTT-CAGATAGAAGATATATTTTGAAAAAAAAAAAATTATATTT : 109
DE-i5-c20-F  : ACGCAGAGATAAAGAAATAGAGAAATTGGATCGCTTGTGTGTTACCCTTACAGATGAAAAATAATCTTT-CAGATAGAAGATATATTTTGAAAAAAAAAAAATTATATTT : 109
DE-i5-c20-R  : ACGCAGAGATAAAGAAATAGAGAAATTGGATCGCTTGTGTGTTACCCTAACAGATGAAAAATAATCTTT-CAGATAGAAGATATATTTTGAAAAAAAAAAAATTATATTT : 109
DE-i6-c1-F   : ACGCAGAGATAAAGAAATAGAGAAATTGGATCGCTTGTGTGTTACCCTTACAGATGAAAAATAATCTTT-CAGATAGAAGATATATTTTGAAAAAAAAAAAATTATATTT : 109
DE-i6-c1-R   : ACGCAGAGATAAAGAAATAGAGAAATTGGATCGCTTGTGTGTTACCCTTACAGATGAAAAATAATCTTT-CAGATAGAAGATATATTTTGAAAAAAAAAAAATTATATTT : 109
DE-i6-c3-F   : ACGCAGAGATAAAGAAATAGAGAAATTGGATCGCTTGTGTGTTACCCTTACAGATGAAAAATAATCTTT-CAGATAGAAGATATATTTTGAAAAAAAAAAAATTATATTT : 109
DE-i6-c3-R   : ACGCAGAGATAAAGAAATAGAGAAATTGGATCGCTTGTGTGTTACCCTTACAGATGAAAAATAATCTTT-CAGATAGAAGATATATTTTGAAAAAAAAAAAATTATATTT : 109
DE-i6-c4-F   : ACGCAGAGATAAAGAAATAGAGAAATTGGATCGCTTGTGTGTTACCCTTACAGATGAAAAATAATCTTT-CAGATAGAAGATATATTTTGAAAAAAAAAAAATTATATTT : 109
DE-i6-c4-R   : ACGCAGAGATAAAGAAATAGAGAAATTGGATCGCTTGTGTGTTACCCTTACAGATGAAAAATAATCTTT-CAGATAGAAGATATATTTTGAAAAAAAAAAAATTATATTT : 109
DE-i6-c5-F   : ACGCAGAGATAAAGAAATAGAGAAATTGGATCGCTTGTGTGTTACCCTTACAGATGAAAAATAATCTTT-CAGATAGAAGATATATTTTGAAAAAAAAAAAATTATATTT : 109
```

```
DE-i6-c5-R  : ACGCAGAGATAAAGAAATAGAGAAATTGGATCGCTTGTGTGTTACCCTTACAGATGAAAAATAATCTTT-CAGATAGAAGATATATTTTGAAAAAAAAAAAATTATATTT : 109
DE-i6-c6-F  : ACGCAGAGATAAAGAAATAGAGAAATTGGATCGCTTGTGTGTTACCCTTACAGATGAAAAATAATCTTT-CAGATAGAAGATATATTTTGAAAAAAAAAAAATTATATTT : 109
DE-i6-c6-R  : ACGCAGAGATAAAGAAATAGAGAAATTGGATCGCTTGTGTGTTACCCTTACAGATGAAAAATAATCTTT-CAGATAGAAGATATATTTTGAAAAAAAAAAAATTATATTT : 109
DE-i6-c7-F  : ACGCAGAGATAAAGAAATAGAGAAATTGGATCGCTTGTGTGTTACCCTTACAGATGAAAAATAATCTTT-CAGATAGAAGATATATTTTGAAAAAAAAAAAATTATATTT : 109
DE-i6-c7-R  : ACGCAGAGATAAAGAAATAGAGAAATTGGATCGCTTGTGTGTTACCCTTACAGATGAAAAATAATCTTT-CAGATAGAAGATATATTTTGAAAAAAAAAAAATTATATTT : 109
DE-i6-c8-F  : ACGCAGAGATAAAGAAATAGAGAAATTGGATCGCTTGTGTGTTACCCTTACAGATGAAAAATAATCTTT-CAGATAGAAGATATATTTTGAAAAAAAAAAAATTATATTT : 109
DE-i6-c8-R  : ACGCAGAGATAAAGAAATAGAGAAATTGGATCGCTTGTGTGTTACCCTTACAGATGAAAAATAATCTTT-CAGATAGAAGATATATTTTGAAAAAAAAAAAATTATATTT : 109
DE-i6-c9-F  : ACGCAGAGATAAAGAAATAGAGAAATTGGATCGCTTGTGTGTTACCCTTACAGATGAAAAATAATCTTT-CAGATAGAAGATATATTTTGAAAAAAAAAAAATTATATTT : 109
DE-i6-c9-R  : ACGCAGAGATAAAGAAATAGAGAAATTGGATCGCTTGTGTGTTACCCTTACAGATGAAAAATAATCTTT-CAGATAGAAGATATATTTTGAAAAAAAAAAAATTATATTT : 109
DE-i6-c10-F : ACGCAGAGATAAAGAAATAGAGAAATTGGATCGCTTGTGTGTTACCCTTACAGATGAAAAATAATCTTT-CAGATAGAAGATATATTTTGAAAAAAAAAAAATTATATTT : 109
DE-i6-c10-R : ACGCAGAGATAAAGAAATAGAGAAATTGGATCGCTTGTGTGTTACCCTTACAGATGAAAAATAATCTTT-CAGATAGAAGATATATTTTGAAAAAAAAAAAATTATATTT : 109
DE-i7-c11-F : ACGCAGAGATAAAGAAATAGAGAAATTGGATCGCTTGTGTGTTACCCTTACAGATGAAAAATAATCTTTACAGATAGAAGATATATTTTGAAAAAAAAAAAATTATATTT : 110
DE-i7-c11-R : ACGCAGAGATAAAGAAATAGAGAAATTGGATCGCTTGTGTGTTACCCTTACAGATGAAAAATAATCTTTACAGATAGAAGATATATTTTGAAAAAAAAAAAATTATATTT : 110
DE-i7-c12-F : ACGCAGAGATAAAGAAATAGAGAAATTGGATCGCTTGTGTGTTACCCTTACAGATGAAAAATAATCTTTACAGATAGAAGATATATTTTGAAAAAAAAAAAATTATATTT : 110
DE-i7-c12-R : ACGCAGAGATAAAGAAATAGAGAAATTGGATCGCTTGTGTGTTACCCTTACAGATGAAAAATAATCTTTACAGATAGAAGATATATTTTGAAAAAAAAAAAATTATATTT : 110
DE-i7-c13-F : ACGCAGAGATAAAGAAATAGAGAAATTGGATCGCTTGTGTGTTACCCTTACAGATGAAAAATAATCTTTACAGATAGAAGATATATTTTGAAAAAAAAAAAATTATATTT : 110
DE-i7-c13-R : ACGCAGAGATAAAGAAATAGAGAAATTGGATCGCTTGTGTGTTACCCTTACAGATGAAAAATAATCTTTACAGATAGAAGATATATTTTGAAAAAAAAAAAATTATATTT : 110
DE-i7-c14-F : ACGCAGAGATAAAGAAATAGAGAAATTGGATCGCTTGTGTGTTACCCTTACAGATGAAAAATAATCTTTACAGATAGAAGATATATTTTGAAAAAAAAAAAATTATATTT : 110
DE-i7-c14-R : ACGCAGAGATAAAGAAATAGAGAAATTGGATCGCTTGTGTGTTACCCTTACAGATGAAAAATAATCTTTACAGATAGAAGATATATTTTGAAAAAAAAAAAATTATATTT : 110
DE-i7-c17-F : ACGCAGAGATAAAGAAATAGAGAAATTGGATCGCTTGTGTGTTACCCTTACAGATGAAAAATAATCTTTACAGATAGAAGATATATTTTGAAAAAAAAAAAATTATATTT : 110
DE-i7-c17-R : ACGCAGAGATAAAGAAATAGAGAAATTGGATCGCTTGTGTGTTACCCTTACAGATGAAAAATAATCTTTACAGATAGAAGATATATTTTGAAAAAAAAAAAATTATATTT : 110
DE-i7-c20-F : ACGCAGAGATAAAGAAATAGAGAAATTGGATCGCTTGTGTGTTACCCTTACAGATGAAAAATAATCTTTACAGATAGAAGATATATTTTGAAAAAAAAAAAATTATATTT : 110
DE-i7-c20-R : ACGCAGAGATAAAGAAATAGAGAAATTGGATCGCTTGTGTGTTACCCTTACAGATGAAAAATAATCTTTACAGATAGAAGATATATTTTGAAAAAAAAAAAATTATATTT : 110
              ACGCAGAGATAAAGAAATAGAGAAATTGGATCGCTTGTGTGTTACCCTtACAGATGAAAAATAATCTTT CAGATAGAAGATATATTTTGAAAAAAAAAAAATTATATTT

                    120         *       140         *       160         *       180         *       200         *       220
DE          : GAAATTTTAATTCAATTAGAAATTAATATAATTTATATAATAGAACAACATTATTTATATTATATATATTATTTATATTATATATATTATTTATATTATATATATTAT-- : 217
DE-i1-c11-F : GAAATTTTAATTCAATTAGAAATTAATATAATTTATATAATAGAACAACATTATTTATATTATATATATTATTTATATTATATATATTATTTATATTATATATATTATTT : 219
DE-i1-c11-R : GAAATTTTAATTCAATTAGAAATTAATATAATTTATATAATAGAACAACATTATTTATATTATATATATTATTTATATTATATATATTATTTATATTATATATATTATTT : 219
DE-i1-c13-F : GAAATTTTAATTCAATTAGAAATTAATATAATTTATATAATAGAACAACATTATTTATATTATATATATTATTTATATTATATATATTATTTATATTATATATATTATTT : 219
DE-i1-c13-R : GAAATTTTAATTCAATTAGAAATTAATATAATTTATATAATAGAACAACATTATTTATATTATATATATTATTTATATTATATATATTATTTATATTATATATATTATTT : 219
DE-i1-c14-F : GAAATTTTAATTCAATTAGAAATTAATATAATTTATATAATAGAACAACATTATTTATATTATATATATTATTTATATTATATATATTATTTATATTATATATATTATTT : 219
DE-i1-c14-R : GAAATTTTAATTCAATTAGAAATTAATATAATTTATATAATAGAACAACATTATTTATATTATATATATTATTTATATTATATATATTATTTATATTATATATATTATTT : 219
DE-i1-c15-F : GAAATTTTAATTCAATTAGAAATTAATATAATTTATATAATAGAACAACATTATTTATATTATATATATTATTTATATTATATATATTATTTATATTATATATATTATTT : 219
DE-i1-c15-R : GAAATTTTAATTCAATTAGAAATTAATATAATTTATATAATAGAACAACATTATTTATATTATATATATTATTTATATTATATATATTATTTATATTATATATATTATTT : 219
DE-i1-c16-F : GAAATTTTAATTCAATTAGAAATTAATATAATTTATATAATAGAACAACATTATTTATATTATATATATTATTTATATTATATATATTATTTATATTATATATATTATTT : 219
DE-i1-c16-R : GAAATTTTAATTCAATTAGAAATTAATATAATTTATATAATAGAACAACATTATTTATATTATATATATTATTTATATTATATATATTATTTATATTATATATATTATTT : 219
DE-i1-c17-F : GAAATTTTAATTCAATTAGAAATTAATATAATTTATATAATAGAACAACATTATTTATATTATATATATTATTTATATTATATATATTATTTATATTATATATATTATTT : 219
DE-i1-c17-R : GAAATTTTAATTCAATTAGAAATTAATATAATTTATATAATAGAACAACATTATTTATATTATATATATTATTTATATTATATATATTATTTATATTATATATATTATTT : 219
DE-i1-c18-F : GAAATTTTAATTCAATTAGAAATTAATATAATTTATATAATAGAACAACATTATTTATATTATATATATTATTTATATTATATATATTATTTATATTATATATATTATTT : 219
DE-i1-c18-R : GAAATTTTAATTCAATTAGAAATTAATATAATTTATATAATAGAACAACATTATTTATATTATATATATTATTTATATTATATATATTATTTATATTATATATATTATTT : 219
DE-i5-c11-F : GAAATTTTAATTCAATTAGAAATTAATATAATTTATATAATAGAACAACATTATTTATATTATATATATTATTTATATTATATATATTATTTATATTATATATATTATTT : 219
DE-i5-c11-R : GAAATTTTAATTCAATTAGAAATTAATATAATTTATATAATAGAACAACATTATTTATATTATATATATTATTTATATTATATATATTATTTATATTATATATATTATTT : 219
DE-i5-c12-F : GAAATTTTAATTCAATTAGAAATTAATATAATTTATATAATAGAACAACATTATTTATATTATATATATTATTTATATTATATATATTATTTATATTATATATATTATTT : 219
DE-i5-c12-R : GAAATTTTAATTCAATTAGAAATTAATATAATTTATATAATAGAACAACATTATTTATATTATATATATTATTTATATTATATATATTATTTATATTATATATATTATTT : 219
DE-i5-c13-F : GAAATTTTAATTCAATTAGAAATTAATATAATTTATATAATAGAACAACATTATTTATATTATATATATTATTTATATTATATATATTATTTATATTATATATATTATTT : 219
```

```
DE-i5-c13-R : GAAATTTTAATTCAATTAGAAATTAATATAATTTATATAATAGAACAACATTATTTATATTATATATATTATTTATATTATATATATTATTTATATTATATATATTATTT : 219
DE-i5-c14-F : GAAATTTTAATTCAATTAGAAATTAATATAATTTATATAATAGAACAACATTATTTATATTATATATATTATTTATATTATATATATTATTTATATTATATATATTATTT : 219
DE-i5-c14-R : GAAATTTTAATTCAATTAGAAATTAATATAATTTATATAATAGAACAACATTATTTATATTATATATATTATTTATATTATATATATTATTTATATTATATATATTATTT : 219
DE-i5-c15-F : GAAATTTTAATTCAATTAGAAATTAATATAATTTATATAATAGAACAACATTATTTATATTATATATATTATTTATATTATATATATTATTTATATTATATATATTATTT : 219
DE-i5-c15-R : GAAATTTTAATTCAATTAGAAATTAATATAATTTATATAATAGAACAACATTATTTATATTATATATATTATTTATATTATATATATTATTTATATTATATATATTATTT : 219
DE-i5-c16-F : GAAATTTTAATTCAATTAGAAATTAATATAATTTATATAATAGAACAACATTATTTATATTATATATATTATTTATATTATATATATTATTTATATTATATATATTATTT : 219
DE-i5-c16-R : GAAATTTTAATTCAATTAGAAATTAATATAATTTATATAATAGAACAACATTATTTATATTATATATATTATTTATATTATATATATTATTTATATTATATATATTATTT : 219
DE-i5-c17-F : GAAATTTTAATTCAATTAGAAATTAATATAATTTATATAATAGAACAACATTATTTATATTATATATATTATTTATATTATATATATTATTTATATTATATATATTATTT : 219
DE-i5-c17-R : GAAATTTTAATTCAATTAGAAATTAATATAATTTATATAATAGAACAACATTATTTATATTATATATATTATTTATATTATATATATTATTTATATTATATATATTATTT : 219
DE-i5-c18-F : GAAATTTTAATTCAATTAGAAATTAATATAATTTATATAATAGAACAACATTATTTATATTATATATATTATTTATATTATATATATTATTTATATTATATATATTATTT : 219
DE-i5-c18-R : GAAATTTTAATTCAATTAGAAATTAATATAATTTATATAATAGAACAACATTATTTATATTATATATATTATTTATATTATATATATTATTTATATTATATATATTATTT : 219
DE-i5-c19-F : GAAATTTTAATTCAATTAGAAATTAATATAATTTATATAATAGAACAACATTATTTATATTATATATATTATTTATATTATATATATTATTTATATTATATATATTATTT : 219
DE-i5-c19-R : GAAATTTTAATTCAATTAGAAATTAATATAATTTATATAATAGAACAACATTATTTATATTATATATATTATTTATATTATATATATTATTTATATTATATATATTATTT : 219
DE-i5-c20-F : GAAATTTTAATTCAATTAGAAATTAATATAATTTATATAATAGAACAACATTATTTATATTATATATATTATTTATATTATATATATTATTTATATTATATATATTATTT : 219
DE-i5-c20-R : GAAATTTTAATTCAATTAGAAATTAATATAATTTATATAATAGAACAACATTATTTATATTATATATATTATTTATATTATATATATTATTTATATTATATATATTATTT : 219
DE-i6-c1-F  : GAAATTTTAATTCAATTAGAAATTAATATAATTTATATAATAGAACAACATTATTTATATTATATATATTATTTATATTATATATATTATTTATATTATATATATTATTT : 219
DE-i6-c1-R  : GAAATTTTAATTCAATTAGAAATTAATATAATTTATATAATAGAACAACATTATTTATATTATATATATTATTTATATTATATATATTATTTATATTATATATATTATTT : 219
DE-i6-c3-F  : GAAATTTTAATTCAATTAGAAATTAATATAATTTATATAATAGAACAACATTATTTATATTATATATATTATTTATATTATATATATTATTTATATTATATATATTATTT : 219
DE-i6-c3-R  : GAAATTTTAATTCAATTAGAAATTAATATAATTTATATAATAGAACAACATTATTTATATTATATATATTATTTATATTATATATATTATTTATATTATATATATTATTT : 219
DE-i6-c4-F  : GAAATTTTAATTCAATTAGAAATTAATATAATTTATATAATAGAACAACATTATTTATATTATATATATTATTTATATTATATATATTATTTATATTATATATATTATTT : 219
DE-i6-c4-R  : GAAATTTTAATTCAATTAGAAATTAATATAATTTATATAATAGAACAACATTATTTATATTATATATATTATTTATATTATATATATTATTTATATTATATATATTATTT : 219
DE-i6-c5-F  : GAAATTTTAATTCAATTAGAAATTAATATAATTTATATAATAGAACAACATTATTTATATTATATATATTATTTATATTATATATATTATTTATATTATATATATTATTT : 219
DE-i6-c5-R  : GAAATTTTAATTCAATTAGAAATTAATATAATTTATATAATAGAACAACATTATTTATATTATATATATTATTTATATTATATATATTATTTATATTATATATATTATTT : 219
DE-i6-c6-F  : GAAATTTTAATTCAATTAGAAATTAATATAATTTATATAATAGAACAACATTATTTATATTATATATATTATTTATATTATATATATTATTTATATTATATATATTATTT : 219
DE-i6-c6-R  : GAAATTTTAATTCAATTAGAAATTAATATAATTTATATAATAGAACAACATTATTTATATTATATATATTATTTATATTATATATATTATTTATATTATATATATTATTT : 219
DE-i6-c7-F  : GAAATTTTAATTCAATTAGAAATTAATATAATTTATATAATAGAACAACATTATTTATATTATATATATTATTTATATTATATATATTATTTATATTATATATATTATTT : 219
DE-i6-c7-R  : GAAATTTTAATTCAATTAGAAATTAATATAATTTATATAATAGAACAACATTATTTATATTATATATATTATTTATATTATATATATTATTTATATTATATATATTATTT : 219
DE-i6-c8-F  : GAAATTTTAATTCAATTAGAAATTAATATAATTTATATAATAGAACAACATTATTTATATTATATATATTATTTATATTATATATATTATTTATATTATATATATTATTT : 219
DE-i6-c8-R  : GAAATTTTAATTCAATTAGAAATTAATATAATTTATATAATAGAACAACATTATTTATATTATATATATTATTTATATTATATATATTATTTATATTATATATATTATTT : 219
DE-i6-c9-F  : GAAATTTTAATTCAATTAGAAATTAATATAATTTATATAATAGAACAACATTATTTATATTATATATATTATTTATATTATATATATTATTTATATTATATATATTATTT : 219
DE-i6-c9-R  : GAAATTTTAATTCAATTAGAAATTAATATAATTTATATAATAGAACAACATTATTTATATTATATATATTATTTATATTATATATATTATTTATATTATATATATTATTT : 219
DE-i6-c10-F : GAAATTTTAATTCAATTAGAAATTAATATAATTTATATAATAGAACAACATTATTTATATTATATATATTATTTATATTATATATATTATTTATATTATATATATTATTT : 219
DE-i6-c10-R : GAAATTTTAATTCAATTAGAAATTAATATAATTTATATAATAGAACAACATTATTTATATTATATATATTATTTATATTATATATATTATTTATATTATATATATTATTT : 219
DE-i7-c11-F : GAAATTTTAATTCAATTAGAAATTAATATAATTTATATAATAGAACAACATTATTTATATTATATATATTATTTATATTATATATATTATTTATATTATATATATTATTT : 220
DE-i7-c11-R : GAAATTTTAATTCAATTAGAAATTAATATAATTTATATAATAGAACAACATTATTTATATTATATATATTATTTATATTATATATATTATTTATATTATATATATTATTT : 220
DE-i7-c12-F : GAAATTTTAATTCAATTAGAAATTAATATAATTTATATAATAGAACAACATTATTTATATTATATATATTATTTATATTATATATATTATTTATATTATATATATTATTT : 220
DE-i7-c12-R : GAAATTTTAATTCAATTAGAAATTAATATAATTTATATAATAGAACAACATTATTTATATTATATATATTATTTATATTATATATATTATTTATATTATATATATTATTT : 220
DE-i7-c13-F : GAAATTTTAATTCAATTAGAAATTAATATAATTTATATAATAGAACAACATTATTTATATTATATATATTATTTATATTATATATATTATTTATATTATATATATTATTT : 220
DE-i7-c13-R : GAAATTTTAATTCAATTAGAAATTAATATAATTTATATAATAGAACAACATTATTTATATTATATATATTATTTATATTATATATATTATTTATATTATATATATTATTT : 220
DE-i7-c14-F : GAAATTTTAATTCAATTAGAAATTAATATAATTTATATAATAGAACAACATTATTTATATTATATATATTATTTATATTATATATATTATTTATATTATATATATTATTT : 220
DE-i7-c14-R : GAAATTTTAATTCAATTAGAAATTAATATAATTTATATAATAGAACAACATTATTTATATTATATATATTATTTATATTATATATATTATTTATATTATATATATTATTT : 220
DE-i7-c17-F : GAAATTTTAATTCAATTAGAAATTAATATAATTTATATAATAGAACAACATTATTTATATTATATATATTATTTATATTATATATATTATTTATATTATATATATTATTT : 220
DE-i7-c17-R : GAAATTTTAATTCAATTAGAAATTAATATAATTTATATAATAGAACAACATTATTTATATTATATATATTATTTATATTATATATATTATTTATATTATATATATTATTT : 220
DE-i7-c20-F : GAAATTTTAATTCAATTAGAAATTAATATAATTTATATAATAGAACAACATTATTTATATTATATATATTATTTATATTATATATATTATTTATATTATATATATTATTT : 220
DE-i7-c20-R : GAAATTTTAATTCAATTAGAAATTAATATAATTTATATAATAGAACAACATTATTTATATTATATATATTATTTATATTATATATATTATTTATATTATATATATTATTT : 220
              GAAATTTTAATTCAATTAGAAATTAATATAATTTATATAATAGAACAACATTATTTATATTATATATATTATTTATATTATATATATTATTTATATTATATATATTATtt
```

```
                   *      240       *      260        *      280       *      300        *     320        *
DE          : -----A-----------TTATATATATTATATAGCATATATATAACAAACATTAACAAACATATAGAAAAAAATAAGAATATAAATAAATTTTTATAAGAAATAGGATTT : 311
DE-i1-c11-F : ATATTATATATATTATATTATATATATTATATAGCATATATATAACAAACATTAACAAACATATAGAAAAAAATAAGAATATAAATAAATTTTTATAAGAAATAGGATTT : 329
DE-i1-c11-R : ATATTATATATATTATATTATATATATTATATAGCATATATATAACAAACATTAACAAACATATAGAAAAAAATAAGAATATAAATAAATTTTTATAAGAAATAGGATTT : 329
DE-i1-c13-F : ATATTATATATATTATATTATATATATTATATAGCATATATATAACAAACATTAACAAACATATAGAAAAAAATAAGAATATAAATAAATTTTTATAAGAAATAGGATTT : 329
DE-i1-c13-R : ATATTATATATATTATATTATATATATTATATAGCATATATATAACAAACATTAACAAACATATAGAAAAAAATAAGAATATAAATAAATTTTTATAAGAAATAGGATTT : 329
DE-i1-c14-F : ATATTATATATATTATATTATATATATTATATAGCATATATATAACAAACATTAACAAACATATAGAAAAAAATAAGAATATAAATAAATTTTTATAAGAAATAGGATTT : 329
DE-i1-c14-R : ATATTATATATATTATATTATATATATTATATAGCATATATATAACAAACATTAACAAACATATAGAAAAAAATAAGAATATAAATAAATTTTTATAAGAAATAGGATTT : 329
DE-i1-c15-F : ATATTATATATATTATATTATATATATTATATAGCATATATATAACAAACATTAACAAACATATAGAAAAAAATAAGAATATAAATAAATTTTTATAAGAAATAGGATTT : 329
DE-i1-c15-R : ATATTATATATATTATATTATATATATTATATAGCATATATATAACAAACATTAACAAACATATAGAAAAAAATAAGAATATAAATAAATTTTTATAAGAAATAGGATTT : 329
DE-i1-c16-F : ATATTATATATATTATATTATATATATTATATAGCATATATATAACAAACATTAACAAACATATAGAAAAAAATAAGAATATAAATAAATTTTTATAAGAAATAGGATTT : 329
DE-i1-c16-R : ATATTATATATATTATATTATATATATTATATAGCATATATATAACAAACATTAACAAACATATAGAAAAAAATAAGAATATAAATAAATTTTTATAAGAAATAGGATTT : 329
DE-i1-c17-F : ATATTATATATATTATATTATATATATTATATAGCATATATATAACAAACATTAACAAACATATAGAAAAAAATAAGAATATAAATAAATTTTTATAAGAAATAGGATTT : 329
DE-i1-c17-R : ATATTATATATATTATATTATATATATTATATAGCATATATATAACAAACATTAACAAACATATAGAAAAAAATAAGAATATAAATAAATTTTTATAAGAAATAGGATTT : 329
DE-i1-c18-F : ATATTATATATATTATATTATATATATTATATAGCATATATATAACAAACATTAACAAACATATAGAAAAAAATAAGAATATAAATAAATTTTTATAAGAAATAGGATTT : 329
DE-i1-c18-R : ATATTATATATATTATATTATATATATTATATAGCATATATATAACAAACATTAACAAACATATAGAAAAAAATAAGAATATAAATAAATTTTTATAAGAAATAGGATTT : 329
DE-i5-c11-F : ATATTATA---------TTATATATATTATATAGCATATATATAACAAACATTAACAAACATATAGAAAAAAATAAGAATATAAATAAATTTTTATAAGAAATAGGATTT : 320
DE-i5-c11-R : ATATTATA---------TTATATATATTATATAGCATATATATAACAAACATTAACAAACATATAGAAAAAAATAAGAATATAAATAAATTTTTATAAGAAATAGGATTT : 320
DE-i5-c12-F : ATATTATA---------TTATATATATTATATAGCATATATATAACAAACATTAACAAACATATAGAAAAAAATAAGAATATAAATAAATTTTTATAAGAAATAGGATTT : 320
DE-i5-c12-R : ATATTATA---------TTATATATATTATATAGCATATATATAACAAACATTAACAAACATATAGAAAAAAATAAGAATATAAATAAATTTTTATAAGAAATAGGATTT : 320
DE-i5-c13-F : ATATTATA---------TTATATATATTATATAGCATATATATAACAAACATTAACAAACATATAGAAAAAAATAAGAATATAAATAAATTTTTATAAGAAATAGGATTT : 320
DE-i5-c13-R : ATATTATA---------TTATATATATTATATAGCATATATATAACAAACATTAACAAACATATAGAAAAAAATAAGAATATAAATAAATTTTTATAAGAAATAGGATTT : 320
DE-i5-c14-F : ATATTATA---------TTATATATATTATATAGCATATATATAACAAACATTAACAAACATATAGAAAAAAATAAGAATATAAATAAATTTTTATAAGAAATAGGATTT : 320
DE-i5-c14-R : ATATTATA---------TTATATATATTATATAGCATATATATAACAAACATTAACAAACATATAGAAAAAAATAAGAATATAAATAAATTTTTATAAGAAATAGGATTT : 320
DE-i5-c15-F : ATATTATA---------TTATATATATTATATAGCATATATATAACAAACATTAACAAACATATAGAAAAAAATAAGAATATAAATAAATTTTTATAAGAAATAGGATTT : 320
DE-i5-c15-R : ATATTATA---------TTATATATATTATATAGCATATATATAACAAACATTAACAAACATATAGAAAAAAATAAGAATATAAATAAATTTTTATAAGAAATAGGATTT : 320
DE-i5-c16-F : ATATTATA---------TTATATATATTATATAGCATATATATAACAAACATTAACAAACATATAGAAAAAAATAAGAATATAAATAAATTTTTATAAGAAATAGGATTT : 320
DE-i5-c16-R : ATATTATA---------TTATATATATTATATAGCATATATATAACAAACATTAACAAACATATAGAAAAAAATAAGAATATAAATAAATTTTTATAAGAAATAGGATTT : 320
DE-i5-c17-F : ATATTATA---------TTATATATATTATATAGCATATATATAACAAACATTAACAAACATATAGAAAAAAATAAGAATATAAATAAATTTTTATAAGAAATAGGATTT : 320
DE-i5-c17-R : ATATTATA---------TTATATATATTATATAGCATATATATAACAAACATTAACAAACATATAGAAAAAAATAAGAATATAAATAAATTTTTATAAGAAATAGGATTT : 320
DE-i5-c18-F : ATATTATA---------TTATATATATTATATAGCATATATATAACAAACATTAACAAACATATAGAAAAAAATAAGAATATAAATAAATTTTTATAAGAAATAGGATTT : 320
DE-i5-c18-R : ATATTATA---------TTATATATATTATATAGCATATATATAACAAACATTAACAAACATATAGAAAAAAATAAGAATATAAATAAATTTTTATAAGAAATAGGATTT : 320
DE-i5-c19-F : ATATTATA---------TTATATATATTATATAGCATATATATAACAAACATTAACAAACATATAGAAAAAAATAAGAATATAAATAAATTTTTATAAGAAATAGGATTT : 320
DE-i5-c19-R : ATATTATA---------TTATATATATTATATAGCATATATATAACAAACATTAACAAACATATAGAAAAAAATAAGAATATAAATAAATTTTTATAAGAAATAGGATTT : 320
DE-i5-c20-F : ATATTATA---------TTATATATATTATATAGCATATATATAACAAACATTAACAAACATATAGAAAAAAATAAGAATATAAATAAATTTTTATAAGAAATAGGATTT : 320
DE-i5-c20-R : ATATTATA---------TTATATATATTATATAGCATATATATAACAAACATTAACAAACATATAGAAAAAAATAAGAATATAAATAAATTTTTATAAGAAATAGGATTT : 320
DE-i6-c1-F  : ATATTATATATATTATATTATATATATTATATAGCATATATATAACAAACATTAACAAACATATAGAAAAAAATAAGAATATAAATAAATTTTTATAAGAAATAGGATTT : 329
DE-i6-c1-R  : ATATTATATATATTATATTATATATATTATATAGCATATATATAACAAACATTAACAAACATATAGAAAAAAATAAGAATATAAATAAATTTTTATAAGAAATAGGATTT : 329
DE-i6-c3-F  : ATATTATATATATTATATTATATATATTATATAGCATATATATAACAAACATTAACAAACATATAGAAAAAAATAAGAATATAAATAAATTTTTATAAGAAATAGGATTT : 329
DE-i6-c3-R  : ATATTATATATATTATATTATATATATTATATAGCATATATATAACAAACATTAACAAACATATAGAAAAAAATAAGAATATAAATAAATTTTTATAAGAAATAGGATTT : 329
DE-i6-c4-F  : ATATTATATATATTATATTATATATATTATATAGCATATATATAACAAACATTAACAAACATATAGAAAAAAATAAGAATATAAATAAATTTTTATAAGAAATAGGATTT : 329
DE-i6-c4-R  : ATATTATATATATTATATTATATATATTATATAGCATATATATAACAAACATTAACAAACATATAGAAAAAAATAAGAATATAAATAAATTTTTATAAGAAATAGGATTT : 329
DE-i6-c5-F  : ATATTATATATATTATATTATATATATTATATAGCATATATATAACAAACATTAACAAACATATAGAAAAAAATAAGAATATAAATAAATTTTTATAAGAAATAGGATTT : 329
DE-i6-c5-R  : ATATTATATATATTATATTATATATATTATATAGCATATATATAACAAACATTAACAAACATATAGAAAAAAATAAGAATATAAATAAATTTTTATAAGAAATAGGATTT : 329
DE-i6-c6-F  : ATATTATATATATTATATTATATATATTATATAGCATATATATAACAAACATTAACAAACATATAGAAAAAAATAAGAATATAAATAAATTTTTATAAGAAATAGGATTT : 329
```

```
DE-i6-c6-R  : ATATTATATATATTATATTATATATATTATATAGCATATATATAACAAACATTAACAAACATATAGAAAAAAATAAGAATATAAATAAATTTTTATAAGAAATAGGATTT : 329
DE-i6-c7-F  : ATATTATATATATTATATTATATATATTATATAGCATATATATAACAAACATTAACAAACATATAGAAAAAAATAAGAATATAAATAAATTTTTATAAGAAATAGGATTT : 329
DE-i6-c7-R  : ATATTATATATATTATATTATATATATTATATAGCATATATATAACAAACATTAACAAACATATAGAAAAAAATAAGAATATAAATAAATTTTTATAAGAAATAGGATTT : 329
DE-i6-c8-F  : ATATTATATATATTATATTATATATATTATATAGCATATATATAACAAACATTAACAAACATATAGAAAAAAATAAGAATATAAATAAATTTTTATAAGAAATAGGATTT : 329
DE-i6-c8-R  : ATATTATATATATTATATTATATATATTATATAGCATATATATAACAAACATTAACAAACATATAGAAAAAAATAAGAATATAAATAAATTTTTATAAGAAATAGGATTT : 329
DE-i6-c9-F  : ATATTATATATATTATATTATATATATTATATAGCATATATATAACAAACATTAACAAACATATAGAAAAAAATAAGAATATAAATAAATTTTTATAAGAAATAGGATTT : 329
DE-i6-c9-R  : ATATTATATATATTATATTATATATATTATATAGCATATATATAACAAACATTAACAAACATATAGAAAAAAATAAGAATATAAATAAATTTTTATAAGAAATAGGATTT : 329
DE-i6-c10-F : ATATTATATATATTATATTATATATATTATATAGCATATATATAACAAACATTAACAAACATATAGAAAAAAATAAGAATATAAATAAATTTTTATAAGAAATAGGATTT : 329
DE-i6-c10-R : ATATTATATATATTATATTATATATATTATATAGCATATATATAACAAACATTAACAAACATATAGAAAAAAATAAGAATATAAATAAATTTTTATAAGAAATAGGATTT : 329
DE-i7-c11-F : ATATTATATATATTATATTATATATATTATATAGCATATATATAACAAACATTAACAAACATATAGAAAAAAATAAGAATATAAATAAATTTTTATAAGAAATAGGATTT : 330
DE-i7-c11-R : ATATTATATATATTATATTATATATATTATATAGCATATATATAACAAACATTAACAAACATATAGAAAAAAATAAGAATATAAATAAATTTTTATAAAAAATAGGATTT : 330
DE-i7-c12-F : ATATTATATATATTATATTATATATATTATATAGCATATATATAACAAACATTAACAAACATATAGAAAAAAATAAGAATATAAATAAATTTTTATAAGAAATAGGATTT : 330
DE-i7-c12-R : ATATTATATATATTATATTATATATATTATATAGCATATATATAACAAACATTAACAAACATATAGAAAAAAATAAGAATATAAATAAATTTTTATAAGAAATAGGATTT : 330
DE-i7-c13-F : ATATTATATATATTATATTATATATATTATATAGCATATATATAACAAACATTAACAAACATATAGAAAAAAATAAGAATATAAATAAATTTTTATAAGAAATAGGATTT : 330
DE-i7-c13-R : ATATTATATATATTATATTATATATATTATATAGCATATATATAACAAACATTAACAAACATATAGAAAAAAATAAGAATATAAATAAATTTTTATAAGAAATAGGATTT : 330
DE-i7-c14-F : ATATTATATATATTATATTATATATATTATATAGCATATATATAACAAACATTAACAAACATATAGAAAAAAATAAGAATATAAATAAATTTTTATAAGAAATAGGATTT : 330
DE-i7-c14-R : ATATTATATATATTATATTATATATATTATATAGCATATATATAACAAACATTAACAAACATATAGAAAAAAATAAGAATATAAATAAATTTTTATAAGAAATAGGATTT : 330
DE-i7-c17-F : ATATTATATATATTATATTATATATATTATATAGCATATATATAACAAACATTAACAAACATATAGAAAAAAATAAGAATATAAATAAATTTTTATAAGAAATAGGATTT : 330
DE-i7-c17-R : ATATTATATATATTATATTATATATATTATATAGCATATATATAACAAACATTAACAAACATATAGAAAAAAATAAGAATATAAATAAATTTTTATAAGAAATAGGATTT : 330
DE-i7-c20-F : ATATTATATATATTATATTATATATATTATATAGCATATATATAACAAACATTAACAAACATATAGAAAAAAATAAGAATATAAATAAATTTTTATAAGAAATAGGATTT : 330
DE-i7-c20-R : ATATTATATATATTATATTATATATATTATATAGCATATATATAACAAACATTAACAAACATATAGAAAAAAATAAGAATATAAATAAATTTTTATAAGAAATAGGATTT : 330
              atattAta         TTATATATATTATATAGCATATATATAACAAACATTAACAAACATATAGAAAAAAATAAGAATATAAATAAATTTTTATAAgAAATAGGATTT

                   340         *       360         *       380         *       400         *       420
DE          : TCGGTATAGGAATAAACAAACCATGGATAAATCTAAGCGACTCTTTCTTAAATCTAAGCAATCTTTTCGTAGGAGTTTGTCCCCGATCCA- : 401
DE-i1-c11-F : TCGGTATAGGAATAAACAAACCATGGATAAATCTAAGCGACTCTTTCTTAAATCTAAGCAATCTTTTCGTAGGAGTTTGTCCCCGATCCA- : 419
DE-i1-c11-R : TCGGTATAGGAATAAACAAACCATGGATAAATCTAAGCGACTCTTTCTTAAATCTAAGCAATCTTTTCGTAGGAGTTTGTCCCCGATCCA- : 419
DE-i1-c13-F : TCGGTATAGGAATAAACAAACCATGGATAAATCTAAGCGACTCTTTCTTAAATCTAAGCAATCTTTTCGTAGGAGTTTGTCCCCGATCCA- : 419
DE-i1-c13-R : TCGGTATAGGAATAAACAAACCATGGATAAATCTAAGCGACTCTTTCTTAAATCTAAGCAATCTTTTCGTAGGAGTTTGTCCCCGATCCA- : 419
DE-i1-c14-F : TCGGTATAGGAATAAACAAACCATGGATAAATCTAAGCGACTCTTTCTTAAATCTAAGCAATCTTTTCGTAGGAGTTTGTCCCCGATCCA- : 419
DE-i1-c14-R : TCGGTATAGGAATAAACAAACCATGGATAAATCTAAGCGACTCTTTCTTAAATCTAAGCAATCTTTTCGTAGGAGTTTGTCCCCGATCCA- : 419
DE-i1-c15-F : TCGGTATAGGAATAAACAAACCATGGATAAATCTAAGCGACTCTTTCTTAAATCTAAGCAATCTTTTCGTAGGAGTTTGTCCCCGATCCA- : 419
DE-i1-c15-R : TCGGTATAGGAATAAACAAACCATGGATAAATCTAAGCGACTCTTTCTTAAATCTAAGCAATCTTTTCGTAGGAGTTTGTCCCCGATCCA- : 419
DE-i1-c16-F : TCGGTATAGGAATAAACAAACCATGGATAAATCTAAGCGACTCTTTCTTAAATCTAAGCAATCTTTTCGTAGGAGTTTGTCCCCGATCCA- : 419
DE-i1-c16-R : TCGGTATAGGAATAAACAAACCATGGATAAATCTAAGCGACTCTTTCTTAAATCTAAGCAATCTTTTCGTAGGAGTTTGTCCCCGATCCA- : 419
DE-i1-c17-F : TCGGTATAGGAATAAACAAACCATGGATAAATCTAAGCGACTCTTTCTTAAATCTAAGCAATCTTTTCGTAGGAGTTTGTCCCCGATCCA- : 419
DE-i1-c17-R : TCGGTATAGGAATAAACAAACCATGGATAAATCTAAGCGACTCTTTCTTAAATCTAAGCAATCTTTTCGTAGGAGTTTGTCCCCGATCCA- : 419
DE-i1-c18-F : TCGGTATAGGAATAAACAAACCATGGATAAATCTAAGCGACTCTTTCTTAAATCTAAGCAATCTTTTCGTAGGAGTTTGTCCCCGATCCA- : 419
DE-i1-c18-R : TCGGTATAGGAATAAACAAACCATGGATAAATCTAAGCGACTCTTTCTTAAATCTAAGCAATCTTTTCGTAGGAGTTTGTCCCCGATCCA- : 419
DE-i5-c11-F : TCGGTATAGGAATAAACAAACCATGGATAAATCTAAGCGACTCTTTCTTAAATCTAAGCAATCTTTTCGTAGGAGTTTGTCCCCGATCCA- : 410
DE-i5-c11-R : TCGGTATAGGAATAAACAAACCATGGATAAATCTAAGCGACTCTTTCTTAAATCTAAGCAATCTTTTCGTAGGAGTTTGTCCCCGATCCA- : 410
DE-i5-c12-F : TCGGTATAGGAATAAACAAACCATGGATAAATCTAAGCGACTCTTTCTTAAATCTAAGCAATCTTTTCGTAGGAGTTTGTCCCCGATCCAA : 411
DE-i5-c12-R : TCGGTATAGGAATAAACAAACCATGGATAAATCTAAGCGACTCTTTCTTAAATCTAAGCAATCTTTTCGTAGGAGTTTGTCCCCGATCCAA : 411
DE-i5-c13-F : TCGGTATAGGAATAAACAAACCATGGATAAATCTAAGCGACTCTTTCTTAAATCTAAGCAATCTTTTCGTAGGAGTTTGTCCCCGATCCAA : 411
DE-i5-c13-R : TCGGTATAGGAATAAACAAACCATGGATAAATCTAAGCGACTCTTTCTTAAATCTAAGCAATCTTTTCGTAGGAGTTTGTCCCCGATCCAA : 411
DE-i5-c14-F : TCGGTATAGGAATAAACAAACCATGGATAAATCTAAGCGACTCTTTCTTAAATCTAAGCAATCTTTTCGTAGGAGTTTGTCCCCGATCCA- : 410
```

```
DE-i5-c14-R : TCGGTATAGGAATAAACAAACCATGGATAAATCTAAGCGACTCTTTCTTAAATCTAAGCAATCTTTTCGTAGGAGTTTGTCCCCGATCCA- : 410
DE-i5-c15-F : TCGGTATAGGAATAAACAAACCATGGATAAATCTAAGCGACTCTTTCTTAAATCTAAGCAATCTTTTCGTAGGAGTTTGTCCCCGATCCAA : 411
DE-i5-c15-R : TCGGTATAGGAATAAACAAACCATGGATAAATCTAAGCGACTCTTTCTTAAATCTAAGCAATCTTTTCGTAGGAGTTTGTCCCCGATCCAA : 411
DE-i5-c16-F : TCGGTATAGGAATAAACAAACCATGGATAAATCTAAGCGACTCTTTCTTAAATCTAAGCAATCTTTTCGTAGGAGTTTGTCCCCGATCCAA : 411
DE-i5-c16-R : TCGGTATAGGAATAAACAAACCATGGATAAATCTAAGCGACTCTTTCTTAAATCTAAGCAATCTTTTCGTAGGAGTTTGTCCCCGATCCAA : 411
DE-i5-c17-F : TCGGTATAGGAATAAACAAACCATGGATAAATCTAAGCGACTCTTTCTTAAATCTAAGCAATCTTTTCGTAGGAGTTTGTCCCCGATCCA- : 410
DE-i5-c17-R : TCGGTATAGGAATAAACAAACCATGGATAAATCTAAGCGACTCTTTCTTAAATCTAAGCAATCTTTTCGTAGGAGTTTGTCCCCGATCCA- : 410
DE-i5-c18-F : TCGGTATAGGAATAAACAAACCATGGATAAATCTAAGCGACTCTTTCTTAAATCTAAGCAATCTTTTCGTAGGAGTTTGTCCCCGATCCAA : 411
DE-i5-c18-R : TCGGTATAGGAATAAACAAACCATGGATAAATCTAAGCGACTCTTTCTTAAATCTAAGCAATCTTTTCGTAGGAGTTTGTCCCCGATCCAA : 411
DE-i5-c19-F : TCGGTATAGGAATAAACAAACCATGGATAAATCTAAGCGACTCTTTCTTAAATCTAAGCAATCTTTTCGTAGGAGTTTGTCCCCGATCCAA : 411
DE-i5-c19-R : TCGGTATAGGAATAAACAAACCATGGATAAATCTAAGCGACTCTTTCTTAAATCTAAGCAATCTTTTCGTAGGAGTTTGTCCCCGATCCAA : 411
DE-i5-c20-F : TCGGTATAGGAATAAACAAACCATGGATAAATCTAAGCGACTCTTTCTTAAATCTAAGCAATCTTTTCGTAGGAGTTTGTCCCCGATCCAA : 411
DE-i5-c20-R : TCGGTATAGGAATAAACAAACCATGGATAAATCTAAGCGACTCTTTCTTAAATCTAAGCAATCTTTTCGTAGGAGTTTGTCCCCGATCCAA : 411
DE-i6-c1-F  : TCGGTATAGGAATAAACAAACCATGGATAAATCTAAGCGACTCTTTCTTAAATCTAAGCAATCTTTTCGTAGGAGTTTGTCCCCGATCCA- : 419
DE-i6-c1-R  : TCGGTATAGGAATAAACAAACCATGGATAAATCTAAGCGACTCTTTCTTAAATCTAAGCAATCTTTTCGTAGGAGTTTGTCCCCGATCCA- : 419
DE-i6-c3-F  : TCGGTATAGGAATAAACAAACCATGGATAAATCTAAGCGACTCTTTCTTAAATCTAAGCAATCTTTTCGTAGGAGTTTGTCCCCGATCCA- : 419
DE-i6-c3-R  : TCGGTATAGGAATAAACAAACCATGGATAAATCTAAGCGACTCTTTCTTAAATCTAAGCAATCTTTTCGTAGGAGTTTGTCCCCGATCCA- : 419
DE-i6-c4-F  : TCGGTATAGGAATAAACAAACCATGGATAAATCTAAGCGACTCTTTCTTAAATCTAAGCAATCTTTTCGTAGGAGTTTGTCCCCGATCCA- : 419
DE-i6-c4-R  : TCGGTATAGGAATAAACAAACCATGGATAAATCTAAGCGACTCTTTCTTAAATCTAAGCAATCTTTTCGTAGGAGTTTGTCCCCGATCCA- : 419
DE-i6-c5-F  : TCGGTATAGGAATAAACAAACCATGGATAAATCTAAGCGACTCTTTCTTAAATCTAAGCAATCTTTTCGTAGGAGTTTGTCCCCGATCCA- : 419
DE-i6-c5-R  : TCGGTATAGGAATAAACAAACCATGGATAAATCTAAGCGACTCTTTCTTAAATCTAAGCAATCTTTTCGTAGGAGTTTGTCCCCGATCCA- : 419
DE-i6-c6-F  : TCGGTATAGGAATAAACAAACCATGGATAAATCTAAGCGACTCTTTCTTAAATCTAAGCAATCTTTTCGTAGGAGTTTGTCCCCGATCCA- : 419
DE-i6-c6-R  : TCGGTATAGGAATAAACAAACCATGGATAAATCTAAGCGACTCTTTCTTAAATCTAAGCAATCTTTTCGTAGGAGTTTGTCCCCGATCCA- : 419
DE-i6-c7-F  : TCGGTATAGGAATAAACAAACCATGGATAAATCTAAGCGACTCTTTCTTAAATCTAAGCAATCTTTTCGTAGGAGTTTGTCCCCGATCCA- : 419
DE-i6-c7-R  : TCGGTATAGGAATAAACAAACCATGGATAAATCTAAGCGACTCTTTCTTAAATCTAAGCAATCTTTTCGTAGGAGTTTGTCCCCGATCCA- : 419
DE-i6-c8-F  : TCGGTATAGGAATAAACAAACCATGGATAAATCTAAGCGACTCTTTCTTAAATCTAAGCAATCTTTTCGTAGGAGTTTGTCCCCGATCCA- : 419
DE-i6-c8-R  : TCGGTATAGGAATAAACAAACCATGGATAAATCTAAGCGACTCTTTCTTAAATCTAAGCAATCTTTTCGTAGGAGTTTGTCCCCGATCCA- : 419
DE-i6-c9-F  : TCGGTATAGGAATAAACAAACCATGGATAAATCTAAGCGACTCTTTCTTAAATCTAAGCAATCTTTTCGTAGGAGTTTGTCCCCGATCCA- : 419
DE-i6-c9-R  : TCGGTATAGGAATAAACAAACCATGGATAAATCTAAGCGACTCTTTCTTAAATCTAAGCAATCTTTTCGTAGGAGTTTGTCCCCGATCCA- : 419
DE-i6-c10-F : TCGGTATAGGAATAAACAAACCATGGATAAATCTAAGCGACTCTTTCTTAAATCTAAGCAATCTTTTCGTAGGAGTTTGTCCCCGATCCA- : 419
DE-i6-c10-R : TCGGTATAGGAATAAACAAACCATGGATAAATCTAAGCGACTCTTTCTTAAATCTAAGCAATCTTTTCGTAGGAGTTTGTCCCCGATCCA- : 419
DE-i7-c11-F : TCGGTATAGGAATAAACAAACCATGGATAAATCTAAGCGACTCTTTCTTAAATCTAAGCAATCTTTTCGTAGGAGTTTGTCCCCGATCCA- : 420
DE-i7-c11-R : TCGGTATAGGAATAAACAAACCATGGATAAATCTAAGCGACTCTTTCTTAAATCTAAGCAATCTTTTCGTAGGAGTTTGTCCCCGATCCA- : 420
DE-i7-c12-F : TCGGTATAGGAATAAACAAACCATGGATAAATCTAAGCGACTCTTTCTTAAATCTAAGCAATCTTTTCGTAGGAGTTTGTCCCCG-TCCA- : 419
DE-i7-c12-R : TCGGTATAGGAATAAACAAACCATGGATAAATCTAAGCGACTCTTTCTTAAATCTAAGCAATCTTTTCGTAGGAGTTTGTCCCCG-TCCA- : 419
DE-i7-c13-F : TCGGTATAGGAATAAACAAACCATGGATAAATCTAAGCGACTCTTTCTTAAATCTAAGCAATCTTTTCGTAGGAGTTTGTCCCCGATCCA- : 420
DE-i7-c13-R : TCGGTATAGGAATAAACAAACCATGGATAAATCTAAGCGACTCTTTCTTAAATCTAAGCAATCTTTTCGTAGGAGTTTGTCCCCGATCCA- : 420
DE-i7-c14-F : TCGGTATAGGAATAAACAAACCATGGATAAATCTAAGCGACTCTTTCTTAAATCTAAGCAATCTTTTCGTAGGAGTTTGTCCCCGATCCA- : 420
DE-i7-c14-R : TCGGTATAGGAATAAACAAACCATGGATAAATCTAAGCGACTCTTTCTTAAATCTAAGCAATCTTTTCGTAGGAGTTTGTCCCCGATCCA- : 420
DE-i7-c17-F : TCGGTATAGGAATAAACAAACCATGGATAAATCTAAGCGACTCTTTCTTAAATCTAAGCAATCTTTTCGTAGGAGTTTGTCCCCGATCCA- : 420
DE-i7-c17-R : TCGGTATAGGAATAAACAAACCATGGATAAATCTAAGCGACTCTTTCTTAAATCTAAGCAATCTTTTCGTAGGAGTTTGTCCCCGATCCA- : 420
DE-i7-c20-F : TCGGTATAGGAATAAACAAACCATGGATAAATCTAAGCGACTCTTTCTTAAATCTAAGCAATCTTTTCGTAGGAGTTTGTCCCCGATCCA- : 420
DE-i7-c20-R : TCGGTATAGGAATAAACAAACCATGGATAAATCTAAGCGACTCTTTCTTAAATCTAAGCAATCTTTTCGTAGGAGTTTGTCCCCGATCCA- : 420
              TCGGTATAGGAATAAACAAACCATGGATAAATCTAAGCGACTCTTTCTTAAATCTAAGCAATCTTTTCGTAGGAGTTTGTCCCCGaTCCA
```
